# Supplementary figures and images for: A proteomic-informed view of the changes induced by loss of cellular adherence: The example of mouse macrophages
Source: PLoS One. 2021 May 28;16(5):e0252450. doi: 10.1371/journal.pone.0252450 (PMC8162644; doi:10.1371/journal.pone.0252450)

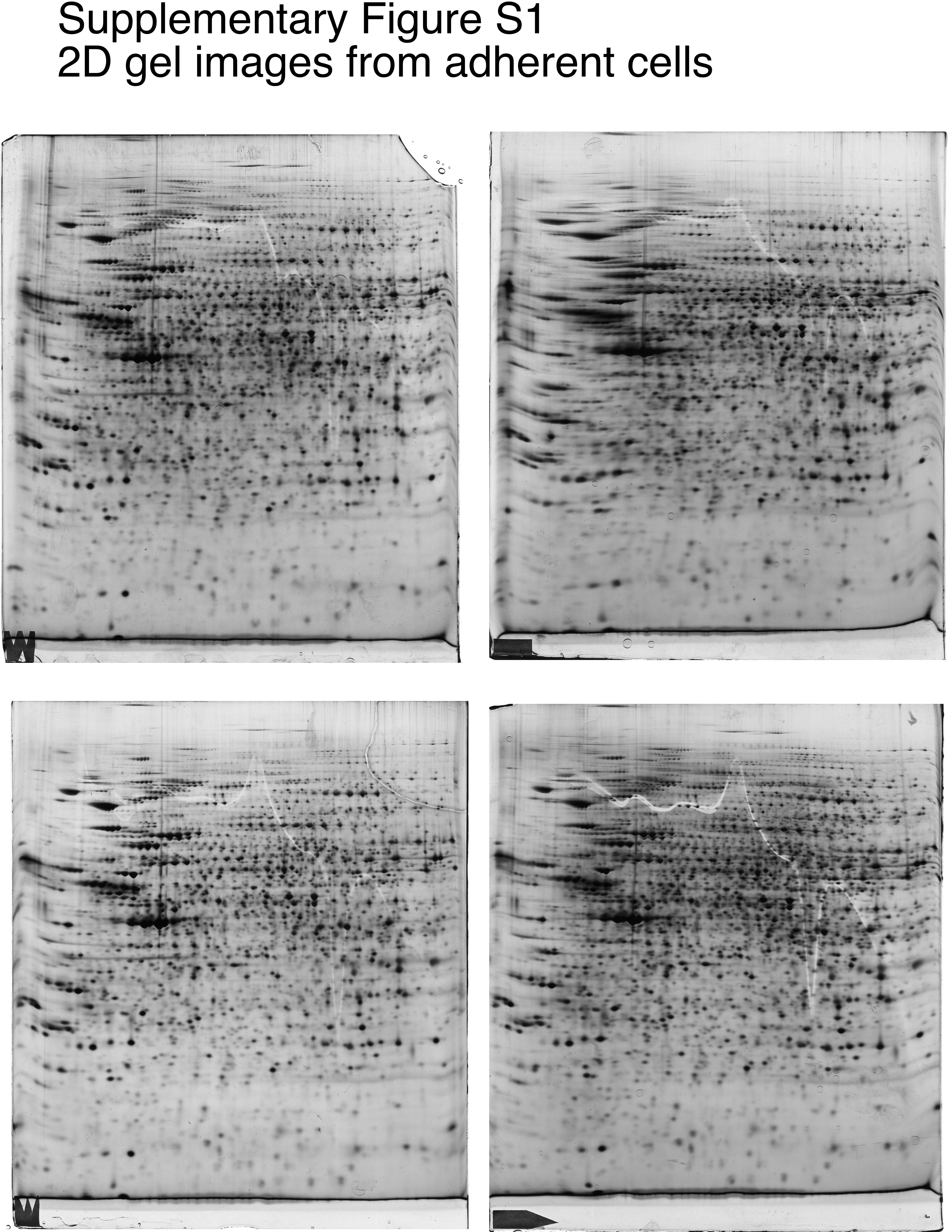

Supplement: S1 Fig — (JPG) [file pone.0252450.s001.jpg]

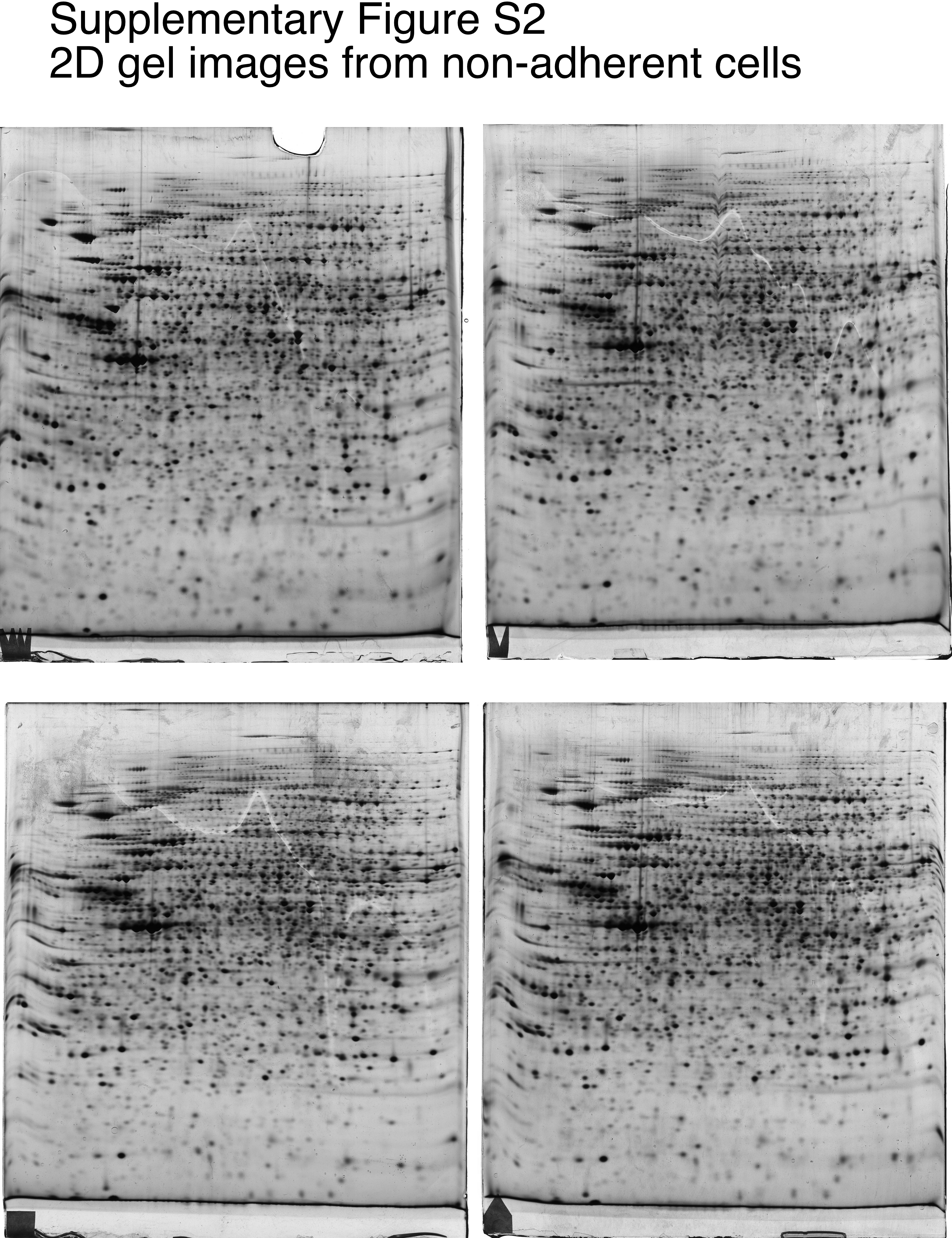

Supplement: S2 Fig — (JPG) [file pone.0252450.s002.jpg]

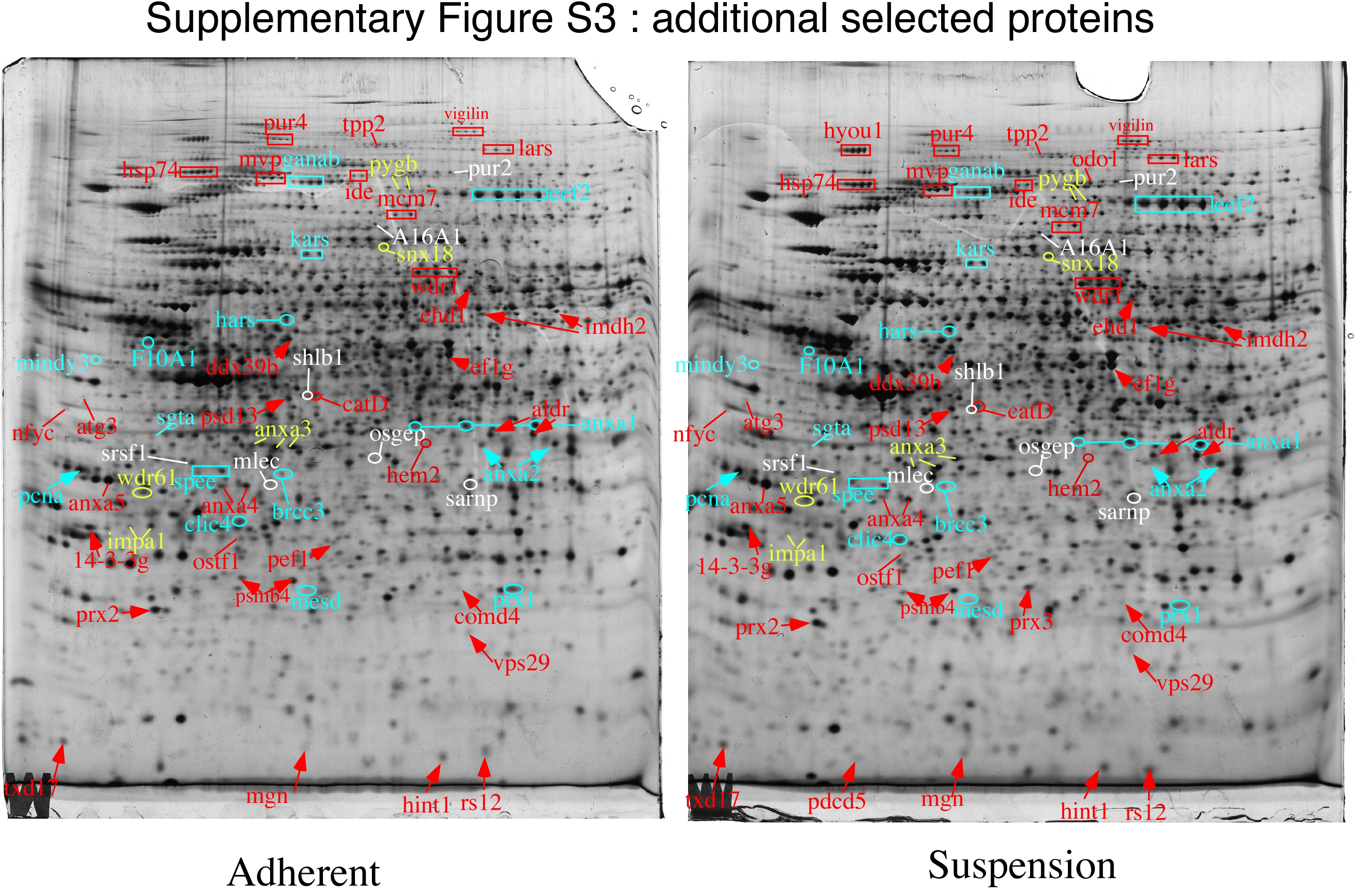

Supplement: S3 Fig — (JPG) [file pone.0252450.s003.jpg]
