# Supplementary material for: A proteomic-informed view of the changes induced by loss of cellular adherence: The example of mouse macrophages
Source: PLoS One. 2021 May 28;16(5):e0252450. doi: 10.1371/journal.pone.0252450 (PMC8162644; doi:10.1371/journal.pone.0252450)
Supplement: S1 Table — (PDF) [file pone.0252450.s004.pdf]

% Volume of Adh1	% Volume of Adh2	% Volume of Adh3	% Volume of Adh4	% Volume of NAD1	% Volume of NAD2	% Volume of NAD3	% Volume of NAD4
0.80341	0.80467	0.74876	0.93716	0.81616	0.79175	0.79434	0.92764
0.61204	0.68165	0.4997	0.60579	0.51614	0.47363	0.57694	0.58989
0.47856	0.52815	0.43155	0.5242	0.50058	0.45846	0.42656	0.47372
0.38219	0.28762	0.18046	0.48703	0.23861	0.29383	0.18957	0.32443
0.37145	0.23105	0.36807	0.37592	0.24704	0.44405	0.49148	0.36239
0.36757	0.45693	0.30007	0.30691	0.34725	0.32355	0.36871	0.31801
0.36276	0.33207	0.29134	0.36508	0.32231	0.28564	0.28444	0.31438
0.35161	0.3332	0.24477	0.404	0.25397	0.28778	0.25435	0.29429
0.33635	0.31424	0.3211	0.32582	0.31357	0.2649	0.26514	0.2576
0.33404	0.26919	0.32428	0.28203	0.32094	0.29848	0.31857	0.29732
0.33179	0.36681	0.28267	0.24567	0.32338	0.29389	0.24558	0.30095
0.32283	0.27602	0.31255	0.28658	0.30259	0.28411	0.26677	0.25465
0.30612	0.2836	0.24504	0.23502	0.25237	0.27728	0.27458	0.27074
0.2891	0.43651	0.30861	0.2888	0.31798	0.28594	0.34421	0.31619
0.28889	0.27105	0.25092	0.27269	0.22982	0.25509	0.25395	0.27742
0.28855	0.33973	0.25054	0.26572	0.18134	0.17134	0.20244	0.21907
0.28589	0.33328	0.30002	0.07116	0.26833	0.24205	0.20529	0.17253
0.28131	0.22872	0.3002	0.25121	0.25465	0.25255	0.24967	0.27402
0.26733	0.24508	0.21816	0.32394	0.24022	0.21306	0.2351	0.23563
0.26629	0.29554	0.19442	0.25695	0.23301	0.23507	0.26093	0.23896
0.25556	0.27824	0.2454	0.3667	0.25	0.23207	0.20897	0.15225
0.25057	0.21094	0.23433	0.24511	0.22355	0.20454	0.21756	0.23419
0.24891	0.2137	0.21603	0.26621	0.22286	0.19676	0.2	0.21488
0.24876	0.27848	0.1963	0.24237	0.21948	0.22002	0.26993	0.19915
0.24674	0.29342	0.21002	0.18802	0.20541	0.18986	0.21118	0.2072
0.2466	0.20621	0.22669	0.25229	0.20163	0.20699	0.17026	0.1961
0.24497	0.26349	0.20616	0.22564	0.18611	0.18932	0.18731	0.1654
0.24419	0.25842	0.24212	0.24587	0.21623	0.22426	0.25594	0.22375
0.24339	0.25387	0.18251	0.28155	0.19224	0.19081	0.21215	0.20684
0.2404	0.24981	0.14888	0.2271	0.17336	0.19818	0.20204	0.17914
0.23818	0.21887	0.2341	0.19695	0.20255	0.20522	0.22761	0.21215
0.23574	0.24616	0.17665	0.21849	0.18987	0.18999	0.18668	0.16321
0.23555	0.29578	0.21615	0.21804	0.21864	0.22164	0.28567	0.21564
0.22821	0.2064	0.22791	0.24781	0.20849	0.19056	0.21216	0.18152
0.22813	0.2868	0.20532	0.23709	0.19611	0.22852	0.23718	0.23143
0.22754	0.22632	0.21062	0.21678	0.18008	0.2049	0.2848	0.2285
0.22707	0.28266	0.18691	0.27096	0.19796	0.18754	0.27297	0.1441
0.22577	0.20116	0.18994	0.23241	0.17284	0.17591	0.17205	0.15936
0.22231	0.13856	0.20655	0.1455	0.14873	0.1176	0.10439	0.15785
0.22199	0.19505	0.22172	0.16679	0.20213	0.20355	0.24211	0.27239
0.22139	0.19047	0.18767	0.19234	0.18328	0.18471	0.19509	0.18987
0.21742	0.25621	0.20181	0.1727	0.17865	0.18373	0.21993	0.1731
0.21693	0.26178	0.20503	0.21137	0.2067	0.19036	0.23162	0.18025
0.21605	0.21821	0.20588	0.23411	0.19678	0.20495	0.21743	0.22701
0.21544	0.27789	0.21705	0.19192	0.23234	0.24211	0.24007	0.19565
0.20967	0.18384	0.18509	0.21736	0.17917	0.16577	0.17759	0.20738
0.20789	0.18052	0.19399	0.2338	0.17529	0.17451	0.1678	0.22041

0.20785	0.24814	0.17993	0.17899	0.16689	0.18898	0.2142	0.17473
0.20773	0.15991	0.17776	0.19638	0.16799	0.16389	0.14476	0.16931
0.20509	0.21399	0.12758	0.08733	0.0974	0.11527	0.11627	0.04874
0.19847	0.23986	0.1753	0.20522	0.19524	0.19132	0.25155	0.20127
0.19677	0.22744	0.18994	0.23683	0.17322	0.17748	0.19108	0.1795
0.19538	0.17509	0.15506	0.19594	0.16322	0.14071	0.13081	0.17943
0.19511	0.14432	0.17478	0.12787	0.18437	0.02818	0.21176	0.20121
0.19509	0.23883	0.19357	0.22454	0.16653	0.16246	0.20349	0.15404
0.19405	0.25833	0.18808	0.21398	0.2032	0.20909	0.25916	0.18524
0.1931	0.23298	0.15701	0.21417	0.16112	0.16578	0.15472	0.15366
0.19279	0.17578	0.16014	0.12906	0.15037	0.15518	0.15714	0.15174
0.18998	0.17815	0.1893	0.1589	0.1848	0.19863	0.23469	0.21158
0.18965	0.1907	0.23147	0.19521	0.17612	0.18702	0.1746	0.17296
0.18944	0.2042	0.16052	0.16567	0.15387	0.15695	0.20181	0.1785
0.1868	0.17216	0.18253	0.13047	0.16892	0.16821	0.16851	0.19006
0.18666	0.21917	0.18277	0.21823	0.24707	0.21347	0.19291	0.25596
0.18577	0.20732	0.16838	0.16266	0.20495	0.11916	0.15338	0.20145
0.18554	0.19612	0.15581	0.1767	0.13232	0.1371	0.18245	0.13976
0.18514	0.17793	0.16408	0.14217	0.15923	0.14942	0.16883	0.18299
0.18251	0.14042	0.14581	0.15938	0.16989	0.15407	0.10784	0.15787
0.18159	0.22048	0.17616	0.17424	0.17206	0.16821	0.18613	0.15301
0.18157	0.21952	0.21749	0.17563	0.18775	0.17465	0.23595	0.19743
0.18077	0.19263	0.15204	0.21195	0.18068	0.1793	0.17307	0.1912
0.17711	0.21014	0.15071	0.16227	0.15912	0.18652	0.20557	0.16178
0.17706	0.21631	0.21391	0.41181	0.25556	0.29187	0.25678	0.20141
0.17705	0.145	0.13727	0.2238	0.13799	0.14098	0.12909	0.14119
0.17677	0.17906	0.17221	0.17098	0.17246	0.18402	0.21503	0.17912
0.17639	0.12742	0.11479	0.17539	0.12289	0.11906	0.09878	0.12754
0.17572	0.20075	0.14404	0.13749	0.15008	0.14962	0.16494	0.14644
0.17365	0.15289	0.17368	0.13345	0.14	0.17115	0.17039	0.13413
0.17165	0.15977	0.16711	0.16693	0.15712	0.17303	0.21212	0.18236
0.17037	0.14956	0.13823	0.16811	0.16228	0.13042	0.11948	0.10941
0.1702	0.14866	0.15255	0.13709	0.14801	0.15369	0.16405	0.17145
0.17006	0.08	0.1295	0.01469	0.03537	0.09835	0.12507	0.03109
0.16938	0.19406	0.13373	0.15813	0.15951	0.14626	0.17357	0.13834
0.16839	0.13868	0.16479	0.16457	0.15584	0.14829	0.14487	0.17458
0.16757	0.22548	0.15567	0.12734	0.1675	0.16808	0.21299	0.14651
0.16752	0.1483	0.15226	0.17807	0.1409	0.13649	0.13427	0.13361
0.16742	0.15457	0.1168	0.16523	0.13544	0.12352	0.15305	0.14284
0.165	0.16386	0.19965	0.17773	0.16652	0.18958	0.17259	0.1814
0.16498	0.21228	0.13652	0.13097	0.16196	0.15867	0.14593	0.13518
0.1647	0.15216	0.10562	0.13814	0.10644	0.12525	0.12292	0.11154
0.16452	0.21355	0.18878	0.1428	0.19257	0.17814	0.18217	0.1975
0.16427	0.13488	0.15309	0.15315	0.14153	0.13225	0.12538	0.12407
0.16338	0.14642	0.17145	0.16188	0.1587	0.1529	0.14647	0.17827
0.16106	0.17227	0.16414	0.1823	0.17414	0.15557	0.16142	0.17099
0.15959	0.18676	0.17446	0.20907	0.15984	0.20018	0.17594	0.15905
0.15851	0.13406	0.11918	0.21448	0.09907	0.11927	0.14933	0.15043
0.15754	0.13082	0.15638	0.16637	0.16396	0.16624	0.15439	0.17939

0.15728	0.16646	0.13329	0.15465	0.13497	0.12439	0.13054	0.12494
0.15692	0.15215	0.17698	0.11226	0.19383	0.18134	0.20835	0.18413
0.15641	0.10825	0.13241	0.14636	0.12962	0.12536	0.1224	0.12486
0.15478	0.13875	0.13848	0.15179	0.1343	0.13003	0.15367	0.12868
0.15391	0.16653	0.2017	0.16361	0.17389	0.20098	0.28281	0.49054
0.15349	0.17024	0.13486	0.13792	0.16695	0.13884	0.14566	0.14878
0.15298	0.1348	0.12048	0.14755	0.13688	0.12388	0.13097	0.12129
0.15273	0.11123	0.09944	0.12968	0.10746	0.11557	0.10253	0.11429
0.15184	0.14137	0.08244	0.0864	0.08707	0.10568	0.09453	0.13734
0.15163	0.09069	0.10762	0.17703	0.09117	0.08645	0.10274	0.10267
0.1509	0.18733	0.11377	0.17319	0.11408	0.13649	0.14221	0.11671
0.14978	0.08218	0.16537	0.10071	0.13242	0.04583	0.13373	0.14927
0.14949	0.13974	0.09987	0.13832	0.10834	0.12954	0.03352	0.08801
0.149	0.14151	0.15531	0.13768	0.14191	0.15615	0.15806	0.14752
0.14749	0.11527	0.11455	0.14179	0.10118	0.10948	0.11183	0.10783
0.14718	0.17917	0.15671	0.15229	0.14802	0.14141	0.14865	0.12504
0.14691	0.13876	0.11425	0.11568	0.10917	0.09778	0.11612	0.1022
0.14673	0.15425	0.14472	0.13261	0.14728	0.14543	0.14606	0.15364
0.1458	0.18398	0.11395	0.15306	0.12362	0.01543	0.04179	0.01792
0.14563	0.13585	0.15328	0.13485	0.13243	0.14159	0.15677	0.15991
0.1446	0.15082	0.12327	0.12896	0.11666	0.13019	0.14882	0.1401
0.14446	0.11041	0.11024	0.16538	0.10637	0.09324	0.10615	0.13869
0.14445	0.14053	0.15093	0.12711	0.13348	0.13598	0.1085	0.14754
0.14379	0.14362	0.12186	0.14589	0.13616	0.11451	0.10276	0.13062
0.14358	0.16267	0.12124	0.12323	0.14543	0.14189	0.16817	0.14711
0.14356	0.14981	0.15696	0.12667	0.14813	0.14819	0.16189	0.13942
0.14353	0.11702	0.17066	0.17628	0.11619	0.1205	0.11315	0.11562
0.14339	0.15027	0.12561	0.12548	0.13502	0.13157	0.13992	0.13323
0.14264	0.1422	0.16731	0.1256	0.15215	0.16795	0.1583	0.1535
0.14135	0.14268	0.11634	0.11927	0.11856	0.11013	0.12265	0.11885
0.14132	0.17311	0.11477	0.13831	0.14188	0.14414	0.1721	0.13511
0.14083	0.12696	0.13791	0.11297	0.13235	0.13775	0.13269	0.14714
0.14007	0.13334	0.16881	0.16113	0.15382	0.17026	0.25127	0.33666
0.13941	0.11472	0.11247	0.1398	0.09142	0.09895	0.0955	0.09427
0.13937	0.13052	0.14117	0.18459	0.11029	0.13394	0.12176	0.13624
0.13872	0.07914	0.06661	0.03773	0.098	0.09448	0.07485	0.02914
0.13827	0.18731	0.15476	0.14116	0.15295	0.13907	0.16734	0.13179
0.13781	0.11929	0.12975	0.13163	0.13241	0.09987	0.12396	0.12104
0.13751	0.12688	0.1789	0.13922	0.13823	0.15703	0.15212	0.13887
0.13616	0.16547	0.16209	0.1957	0.15966	0.14346	0.11721	0.1342
0.13585	0.11925	0.10663	0.12793	0.11144	0.12031	0.09921	0.1563
0.13411	0.1521	0.09856	0.13331	0.12074	0.13394	0.13617	0.12095
0.13385	0.11389	0.10761	0.15102	0.11493	0.1226	0.09951	0.11246
0.1337	0.13421	0.14661	0.18921	0.15241	0.13057	0.09543	0.16041
0.13366	0.12486	0.11428	0.13738	0.12429	0.12539	0.12706	0.11149
0.13262	0.10657	0.10136	0.11429	0.09488	0.10096	0.09992	0.09162
0.13243	0.1387	0.09892	0.09993	0.15729	0.15245	0.22646	0.29053
0.13174	0.12321	0.10635	0.11226	0.09719	0.07989	0.11961	0.09808
0.1311	0.12055	0.10735	0.13934	0.10593	0.11363	0.11922	0.10379

0.13072	0.1161	0.13804	0.13918	0.16234	0.15212	0.19591	0.15587
0.13068	0.12186	0.12944	0.12579	0.10972	0.11835	0.13894	0.13242
0.13045	0.1543	0.11402	0.11323	0.12026	0.11685	0.09306	0.12011
0.1303	0.13613	0.11581	0.13706	0.08438	0.09486	0.08932	0.14915
0.13022	0.20189	0.035	0.16701	0.169	0.10027	0.0254	0.20654
0.12994	0.11037	0.10104	0.12151	0.08755	0.09954	0.11455	0.09191
0.12943	0.13124	0.11581	0.1116	0.10146	0.09015	0.11916	0.10294
0.12943	0.13284	0.11166	0.13762	0.10634	0.10732	0.10508	0.09512
0.1291	0.09302	0.07336	0.13269	0.09173	0.10463	0.10062	0.10033
0.12905	0.09409	0.10088	0.08046	0.0693	0.07463	0.04888	0.04279
0.12821	0.12352	0.12468	0.13846	0.11297	0.11039	0.09365	0.09675
0.1271	0.13319	0.14636	0.11111	0.13087	0.13242	0.14525	0.13794
0.12635	0.16595	0.15483	0.19396	0.16571	0.15984	0.13853	0.13839
0.12518	0.13327	0.15327	0.11209	0.12209	0.15151	0.14222	0.10963
0.12371	0.11783	0.10142	0.11318	0.10228	0.0999	0.1179	0.10599
0.12273	0.12186	0.11565	0.12177	0.11361	0.12889	0.11538	0.12641
0.1219	0.13158	0.14638	0.08023	0.16273	0.15544	0.18257	0.15719
0.12168	0.08261	0.1017	0.12878	0.08196	0.07586	0.06961	0.0769
0.12153	0.19498	0.14475	0.11457	0.1321	0.14309	0.14405	0.12695
0.12112	0.10077	0.10285	0.10526	0.09619	0.11175	0.09611	0.09408
0.12056	0.1462	0.12483	0.12881	0.16822	0.16182	0.23726	0.3799
0.12056	0.10301	0.10528	0.10794	0.10916	0.11023	0.09229	0.1026
0.1205	0.17126	0.14907	0.10809	0.14257	0.12536	0.15903	0.1373
0.12043	0.12216	0.0977	0.08507	0.11162	0.07335	0.12647	0.11033
0.11939	0.09283	0.11793	0.10456	0.07656	0.11021	0.07728	0.07759
0.11931	0.10067	0.11167	0.11909	0.10759	0.10465	0.09958	0.0949
0.11864	0.0981	0.08125	0.11094	0.06239	0.10982	0.07847	0.0893
0.11862	0.10176	0.11158	0.08803	0.09956	0.09519	0.0997	0.09407
0.1184	0.1273	0.09118	0.12512	0.09836	0.10602	0.13842	0.12469
0.11776	0.12022	0.12779	0.12087	0.11439	0.11807	0.10163	0.10887
0.11756	0.15117	0.12605	0.13735	0.12291	0.12001	0.11092	0.1083
0.11743	0.09131	0.10236	0.11842	0.08792	0.08721	0.10073	0.09696
0.11649	0.10948	0.10809	0.10534	0.1074	0.10223	0.10463	0.1031
0.11603	0.12934	0.11729	0.06793	0.11674	0.11993	0.11472	0.11805
0.11544	0.11137	0.099	0.10573	0.09815	0.08587	0.11167	0.09889
0.11531	0.11647	0.13359	0.13163	0.07643	0.11658	0.1123	0.17133
0.11452	0.05091	0.08987	0.09405	0.07948	0.04896	0.05989	0.09768
0.1144	0.1095	0.10002	0.09569	0.10109	0.11199	0.12671	0.11159
0.11398	0.07038	0.11393	0.13011	0.07796	0.07491	0.10592	0.09987
0.11354	0.13129	0.13725	0.12736	0.12535	0.12162	0.14898	0.13446
0.11353	0.09571	0.10013	0.11332	0.08752	0.09919	0.10304	0.11572
0.11324	0.11473	0.10144	0.08612	0.10569	0.10391	0.10518	0.10007
0.11225	0.10397	0.08672	0.1077	0.0951	0.08679	0.09319	0.09425
0.11211	0.12723	0.0926	0.10943	0.11999	0.11516	0.11191	0.07185
0.11198	0.09542	0.09665	0.10494	0.0982	0.09495	0.07892	0.08009
0.11195	0.11688	0.08993	0.1176	0.10808	0.09717	0.11003	0.09485
0.11184	0.09754	0.07576	0.10883	0.07717	0.08163	0.07791	0.09561
0.11157	0.07861	0.08865	0.10496	0.08631	0.09703	0.10344	0.10043
0.11125	0.1009	0.08272	0.12454	0.07863	0.07942	0.08428	0.08381

0.11072	0.09107	0.10176	0.09702	0.08637	0.08834	0.07934	0.09539
0.11068	0.10712	0.10251	0.11209	0.10187	0.10618	0.09079	0.07214
0.10991	0.15568	0.1271	0.13668	0.12505	0.12763	0.1428	0.12034
0.10974	0.10435	0.1054	0.09598	0.11091	0.10031	0.11326	0.10513
0.1082	0.06996	0.09136	0.0844	0.08636	0.06682	0.07562	0.04958
0.10819	0.19058	0.0237	0.14734	0.14284	0.13177	0.03083	0.18091
0.10768	0.11609	0.11952	0.09733	0.11097	0.13828	0.13672	0.13315
0.10763	0.08623	0.09212	0.09689	0.08219	0.10335	0.13408	0.11081
0.10707	0.10295	0.10201	0.09613	0.10117	0.09688	0.10454	0.09433
0.10678	0.0835	0.10368	0.117	0.0871	0.09747	0.08975	0.06891
0.10654	0.12969	0.10744	0.12147	0.11963	0.11441	0.1069	0.11698
0.10557	0.10416	0.08694	0.07069	0.10785	0.11098	0.09151	0.04722
0.10534	0.09462	0.09871	0.12972	0.09669	0.10151	0.13002	0.10452
0.10516	0.09722	0.11722	0.12501	0.10227	0.10411	0.09235	0.10407
0.10514	0.0837	0.07515	0.11956	0.08226	0.08201	0.07349	0.08894
0.10498	0.09714	0.0837	0.09884	0.0931	0.07717	0.10694	0.10043
0.10434	0.11558	0.08825	0.11105	0.10667	0.10171	0.13074	0.08687
0.10413	0.08697	0.08527	0.10036	0.10246	0.11218	0.09951	0.1156
0.10391	0.09546	0.09676	0.11902	0.07029	0.07748	0.05374	0.07855
0.10322	0.10414	0.10272	0.10571	0.09939	0.1026	0.11397	0.11154
0.10316	0.10215	0.14218	0.14524	0.12638	0.14742	0.13133	0.12414
0.10309	0.09376	0.09277	0.08323	0.095	0.09503	0.09025	0.07548
0.10283	0.06709	0.07023	0.11118	0.06914	0.06195	0.05959	0.06859
0.1024	0.09325	0.00831	0.20698	0.07872	0.12701	0.092	0.17659
0.10184	0.0977	0.08681	0.09416	0.08882	0.08923	0.10361	0.10238
0.10179	0.11214	0.14393	0.12816	0.11046	0.11469	0.12111	0.07543
0.10134	0.09092	0.0885	0.08986	0.08775	0.09553	0.10377	0.09108
0.10115	0.09694	0.09536	0.11646	0.09164	0.08896	0.09272	0.09128
0.1011	0.11252	0.06711	0.08942	0.07733	0.07578	0.07882	0.08037
0.10057	0.12224	0.10031	0.11484	0.10436	0.10725	0.11355	0.09108
0.10052	0.19766	0.10746	0.07353	0.10587	0.09897	0.12252	0.099
0.10038	0.10699	0.12076	0.06766	0.10164	0.09708	0.11296	0.08515
0.09922	0.09167	0.07964	0.09477	0.09673	0.08342	0.09134	0.08305
0.099	0.11803	0.08955	0.10249	0.08622	0.0846	0.0807	0.07968
0.09823	0.12083	0.11737	0.09391	0.12952	0.11314	0.12625	0.13588
0.09803	0.06923	0.07478	0.0947	0.07375	0.07076	0.05512	0.05123
0.09795	0.09964	0.11002	0.14131	0.11399	0.10342	0.10229	0.09068
0.09678	0.09242	0.1075	0.10624	0.09496	0.10398	0.11645	0.10625
0.09667	0.11593	0.09829	0.09597	0.10238	0.107	0.11452	0.09925
0.09664	0.07727	0.07643	0.10702	0.07923	0.10386	0.05938	0.05809
0.09655	0.09754	0.09441	0.08609	0.09847	0.09286	0.09727	0.08195
0.09644	0.10919	0.07941	0.07768	0.06853	0.07528	0.06309	0.06619
0.09637	0.07673	0.07797	0.1035	0.08326	0.07918	0.08367	0.07377
0.09604	0.10193	0.08759	0.07268	0.09199	0.08983	0.10586	0.09508
0.09584	0.1713	0.03487	0.27532	0.20503	0.14026	0.02793	0.19487
0.09502	0.07842	0.06385	0.10012	0.10769	0.11262	0.11719	0.08494
0.0944	0.08209	0.08555	0.10023	0.08781	0.09346	0.08719	0.08466
0.09408	0.09413	0.09162	0.08109	0.08959	0.089	0.09566	0.08768
0.09397	0.0319	0.05407	0.05392	0.05724	0.07104	0.04651	0.05656

0.09358	0.07941	0.06369	0.0836	0.07271	0.07376	0.08739	0.07866
0.09314	0.08905	0.08397	0.08919	0.08385	0.09265	0.09726	0.09881
0.09301	0.08388	0.09033	0.08052	0.08426	0.09194	0.09043	0.07975
0.09256	0.07947	0.09388	0.09786	0.09009	0.0876	0.05985	0.10541
0.09229	0.0918	0.09721	0.11069	0.09122	0.08706	0.1036	0.09988
0.09208	0.07237	0.06875	0.08383	0.06306	0.07201	0.05594	0.06091
0.09171	0.09632	0.08414	0.08593	0.09038	0.09287	0.08896	0.10145
0.09148	0.08924	0.0892	0.07197	0.08639	0.08316	0.09339	0.09588
0.09124	0.08418	0.07759	0.08478	0.08127	0.08122	0.07955	0.0949
0.09117	0.08924	0.08071	0.08896	0.0647	0.08726	0.08279	0.064
0.09113	0.07401	0.07748	0.09412	0.08033	0.09015	0.06212	0.07851
0.0908	0.08825	0.07645	0.08686	0.0834	0.07766	0.09982	0.10586
0.09023	0.09279	0.06321	0.05517	0.06686	0.07328	0.06052	0.10243
0.0898	0.1275	0.12807	0.17426	0.11116	0.09994	0.13299	0.30283
0.08909	0.08464	0.07647	0.08131	0.08158	0.07688	0.08808	0.08569
0.08905	0.07543	0.0836	0.07305	0.07291	0.06274	0.06835	0.092
0.08905	0.0746	0.08439	0.09704	0.07842	0.07921	0.07971	0.07394
0.08884	0.08702	0.05861	0.09853	0.06314	0.06624	0.11206	0.06225
0.0888	0.09774	0.07704	0.07425	0.10166	0.09199	0.07037	0.10336
0.08851	0.06773	0.06886	0.09009	0.06781	0.07506	0.08515	0.08228
0.0883	0.08374	0.08608	0.08223	0.09024	0.07147	0.06886	0.06962
0.08807	0.07806	0.08707	0.0834	0.08059	0.08963	0.14602	0.1611
0.0878	0.09769	0.12407	0.07938	0.11307	0.10226	0.11899	0.10087
0.08728	0.06309	0.06722	0.0872	0.05423	0.05936	0.07506	0.05233
0.08721	0.09913	0.08301	0.08819	0.09448	0.10023	0.11417	0.10958
0.08716	0.06859	0.05232	0.03883	0.07843	0.07573	0.08618	0.08199
0.08711	0.07805	0.08393	0.08811	0.07374	0.07786	0.08115	0.07717
0.08677	0.08827	0.08276	0.06486	0.08079	0.0819	0.10042	0.08991
0.08611	0.07763	0.08838	0.10491	0.08821	0.0919	0.07227	0.08765
0.08599	0.08699	0.08121	0.08126	0.06478	0.08924	0.10702	0.07232
0.08583	0.07718	0.08123	0.05875	0.07496	0.08484	0.07691	0.06992
0.08486	0.0784	0.09386	0.10495	0.08367	0.07449	0.05883	0.08082
0.08447	0.03406	0.08155	0.03458	0.07655	0.07829	0.07849	0.06311
0.08445	0.09456	0.0834	0.07341	0.08891	0.08849	0.11353	0.09184
0.08445	0.0834	0.09168	0.07482	0.0745	0.10196	0.08176	0.08448
0.08439	0.06449	0.08518	0.07882	0.07299	0.08271	0.07397	0.06563
0.0841	0.09762	0.11475	0.08187	0.1111	0.12791	0.12617	0.09283
0.08394	0.08906	0.1028	0.06982	0.08764	0.09066	0.09942	0.08402
0.08365	0.0926	0.07687	0.11274	0.10267	0.08926	0.07028	0.06939
0.08354	0.08034	0.07247	0.07948	0.07298	0.07473	0.08017	0.07773
0.08347	0.10195	0.07952	0.08381	0.08937	0.08806	0.09709	0.09204
0.08318	0.06163	0.07911	0.07474	0.08575	0.07154	0.05993	0.06311
0.08316	0.07725	0.05273	0.05547	0.05607	0.06127	0.05968	0.07276
0.08299	0.09774	0.07175	0.0714	0.07931	0.08504	0.09373	0.07715
0.08293	0.08434	0.06906	0.08833	0.06548	0.05358	0.07612	0.0788
0.08285	0.05983	0.04735	0.06634	0.0485	0.07629	0.02251	0.05792
0.08284	0.08534	0.09509	0.08247	0.09109	0.08947	0.07015	0.08577
0.0826	0.09806	0.14031	0.07913	0.11909	0.08742	0.09935	0.09503
0.08252	0.12363	0.09633	0.1265	0.12739	0.1104	0.09749	0.09061

0.08242	0.07033	0.06776	0.08383	0.05219	0.06833	0.06073	0.05743
0.08207	0.07567	0.07721	0.07015	0.07167	0.07546	0.08706	0.0761
0.08184	0.08901	0.08175	0.07498	0.0996	0.08923	0.11452	0.09682
0.08163	0.06383	0.06563	0.09611	0.06975	0.06188	0.07783	0.06608
0.08146	0.06746	0.09172	0.09333	0.09335	0.07507	0.07491	0.05487
0.08129	0.05809	0.06592	0.07756	0.06339	0.06722	0.05991	0.05758
0.08101	0.06911	0.07788	0.0845	0.08329	0.05813	0.07614	0.08083
0.08082	0.06316	0.07833	0.08254	0.07666	0.07272	0.07605	0.08168
0.08068	0.08706	0.11079	0.11111	0.09587	0.09764	0.07632	0.07666
0.08055	0.09683	0.08321	0.05752	0.06482	0.07746	0.07492	0.06199
0.08055	0.05128	0.09591	0.08555	0.10092	0.05242	0.07354	0.05492
0.08047	0.04272	0.07207	0.08486	0.05675	0.05297	0.05541	0.08628
0.0804	0.07471	0.07555	0.07355	0.06372	0.07298	0.06154	0.06319
0.08037	0.07188	0.08096	0.08092	0.07901	0.07775	0.06936	0.06953
0.08019	0.08091	0.07717	0.07386	0.08285	0.08631	0.078	0.08562
0.08017	0.07482	0.05346	0.08204	0.07116	0.09523	0.0503	0.07236
0.08014	0.10334	0.10649	0.07587	0.09827	0.09124	0.0869	0.07349
0.0799	0.07445	0.07163	0.07169	0.07422	0.07145	0.08786	0.0721
0.07987	0.06308	0.06548	0.06296	0.06708	0.06637	0.0824	0.08072
0.0798	0.0673	0.08082	0.09721	0.09493	0.06577	0.07241	0.07817
0.07977	0.0624	0.07331	0.09058	0.08502	0.08094	0.0848	0.07391
0.07972	0.06052	0.06919	0.08487	0.06495	0.07822	0.07404	0.06823
0.07966	0.08791	0.07266	0.06295	0.07101	0.07982	0.08307	0.08691
0.07966	0.05343	0.09187	0.08652	0.0482	0.05559	0.05766	0.07663
0.07954	0.07624	0.06945	0.08019	0.07568	0.07002	0.07637	0.07314
0.07944	0.07052	0.06065	0.07435	0.05791	0.05415	0.06225	0.05777
0.07936	0.06744	0.09318	0.06056	0.06618	0.06624	0.07322	0.05708
0.0793	0.07741	0.09482	0.10034	0.08906	0.08319	0.08338	0.07443
0.07911	0.07876	0.07587	0.09431	0.07761	0.06925	0.10068	0.08171
0.07885	0.09569	0.08798	0.0537	0.09409	0.08738	0.11254	0.08429
0.07881	0.06587	0.05922	0.05019	0.07239	0.07154	0.06215	0.05436
0.07876	0.04242	0.11204	0.02033	0.09819	0.08614	0.08805	0.05387
0.07828	0.09149	0.09919	0.07999	0.11228	0.10696	0.09961	0.11274
0.07819	0.09648	0.0784	0.05624	0.06912	0.09955	0.0664	0.04594
0.07815	0.06827	0.07753	0.07581	0.06596	0.07853	0.10015	0.0776
0.07787	0.06793	0.06784	0.08852	0.07261	0.06505	0.06957	0.07253
0.07784	0.07432	0.05668	0.11293	0.08633	0.09399	0.08044	0.12347
0.07748	0.07351	0.05369	0.07202	0.05573	0.06086	0.06215	0.06155
0.07718	0.06388	0.06988	0.06383	0.06905	0.06605	0.06156	0.07658
0.07696	0.10508	0.07322	0.08786	0.0879	0.08882	0.09207	0.0798
0.07695	0.0784	0.09984	0.07708	0.08408	0.08658	0.08063	0.077
0.07695	0.08994	0.07931	0.06201	0.09261	0.11824	0.09993	0.05927
0.07689	0.0682	0.0781	0.05915	0.0858	0.08119	0.08671	0.08444
0.07688	0.07962	0.08599	0.07848	0.07458	0.07484	0.08308	0.07676
0.07668	0.09903	0.07384	0.06119	0.07162	0.07841	0.09621	0.09132
0.07654	0.08853	0.10036	0.09487	0.09044	0.06658	0.14794	0.22219
0.07648	0.09845	0.03239	0.09473	0.06171	0.05937	0.01727	0.07043
0.07637	0.07623	0.06659	0.09824	0.06269	0.06608	0.06845	0.06866
0.07628	0.07417	0.08167	0.05094	0.07616	0.08339	0.08472	0.06791

0.07627	0.07918	0.06994	0.06847	0.07457	0.06846	0.0635	0.0683
0.07579	0.07233	0.08972	0.16532	0.14194	0.0848	0.0454	0.08098
0.07575	0.08488	0.08064	0.06597	0.06746	0.08462	0.05493	0.05393
0.0755	0.06695	0.07517	0.07526	0.07058	0.06569	0.06918	0.05653
0.07529	0.06765	0.07778	0.07122	0.07002	0.07197	0.06735	0.05919
0.07517	0.11847	0.09099	0.05602	0.08035	0.09064	0.06755	0.0731
0.07514	0.08759	0.09567	0.08148	0.08708	0.09118	0.09566	0.10329
0.0751	0.06851	0.05883	0.08509	0.05747	0.05928	0.04127	0.06176
0.07503	0.05784	0.05355	0.08496	0.04401	0.04326	0.05776	0.06255
0.07481	0.06723	0.06265	0.08076	0.05817	0.06301	0.04685	0.05888
0.07418	0.08268	0.08095	0.06837	0.07909	0.07262	0.07257	0.06607
0.07406	0.09862	0.08699	0.08317	0.09181	0.08242	0.11311	0.10545
0.07353	0.08126	0.05792	0.06585	0.06678	0.06475	0.07059	0.07542
0.07327	0.06326	0.07336	0.07871	0.08047	0.0728	0.06456	0.07429
0.0732	0.06316	0.06056	0.07186	0.05354	0.05761	0.04703	0.05272
0.07318	0.03867	0.05405	0.07354	0.06331	0.05871	0.07759	0.06948
0.07318	0.06862	0.06968	0.07521	0.06201	0.07431	0.07027	0.06614
0.07306	0.06144	0.05203	0.06103	0.0559	0.06056	0.05484	0.06461
0.07298	0.06589	0.07522	0.06698	0.06292	0.07309	0.0714	0.0623
0.07287	0.0742	0.07403	0.06564	0.07612	0.07889	0.0819	0.08122
0.07282	0.07397	0.06059	0.07084	0.06589	0.07434	0.0665	0.07578
0.07247	0.07287	0.05574	0.04173	0.06203	0.05113	0.07651	0.06848
0.07205	0.06132	0.07014	0.06363	0.06095	0.06684	0.07208	0.06554
0.07188	0.11081	0.01597	0.12903	0.1129	0.10127	0.02376	0.18184
0.0718	0.08857	0.0734	0.0633	0.06653	0.0645	0.06182	0.07123
0.07169	0.06449	0.06675	0.0617	0.06756	0.08373	0.06357	0.07152
0.0715	0.0627	0.05465	0.07422	0.04708	0.06523	0.07093	0.06343
0.07137	0.07216	0.06759	0.0721	0.06627	0.06447	0.06972	0.07391
0.07129	0.06788	0.08983	0.06941	0.07813	0.07915	0.07637	0.06981
0.07111	0.0496	0.07011	0.07655	0.0599	0.06603	0.08226	0.03814
0.07092	0.05545	0.07065	0.07582	0.07348	0.06725	0.06394	0.05522
0.07075	0.08666	0.07105	0.16008	0.09347	0.06974	0.04982	0.08339
0.07068	0.06796	0.07148	0.06943	0.06695	0.06444	0.06379	0.05842
0.07037	0.05991	0.05114	0.02362	0.04295	0.04906	0.03018	0.03182
0.0703	0.06071	0.05715	0.06004	0.05032	0.05869	0.04198	0.05624
0.07027	0.083	0.07805	0.04859	0.07405	0.06901	0.09387	0.07675
0.07005	0.06526	0.04717	0.07591	0.0467	0.04419	0.02963	0.0526
0.06992	0.08263	0.07269	0.06114	0.06245	0.0503	0.06854	0.06871
0.06987	0.07932	0.10518	0.09415	0.09368	0.10488	0.08551	0.08454
0.06964	0.09347	0.07364	0.06327	0.086	0.08882	0.07638	0.07963
0.06931	0.05534	0.04859	0.07399	0.04395	0.04706	0.04065	0.05444
0.06927	0.06691	0.0735	0.06756	0.06663	0.06908	0.06292	0.06113
0.06884	0.05864	0.05996	0.07998	0.07434	0.06652	0.0662	0.07788
0.06879	0.0597	0.06537	0.06939	0.06117	0.06969	0.04516	0.06436
0.06876	0.09238	0.06375	0.07387	0.0679	0.09036	0.12834	0.06898
0.06876	0.05873	0.06065	0.04793	0.0582	0.06163	0.05525	0.06195
0.06866	0.06522	0.06119	0.07832	0.04691	0.04	0.03844	0.03908
0.06865	0.14895	0.15678	0.06397	0.12949	0.13531	0.08958	0.13958
0.06858	0.08966	0.06961	0.08641	0.08519	0.10628	0.09358	0.09709

0.0684	0.04551	0.07161	0.11093	0.05957	0.05992	0.07077	0.0739
0.06834	0.04182	0.05008	0.05213	0.05836	0.0485	0.06179	0.03264
0.06826	0.0767	0.06177	0.06441	0.06407	0.06571	0.06819	0.07173
0.06823	0.05988	0.06222	0.05342	0.06261	0.06008	0.05598	0.05877
0.06794	0.06842	0.05488	0.06669	0.06117	0.06372	0.05337	0.06604
0.06773	0.05006	0.05047	0.06045	0.04119	0.04992	0.04118	0.04595
0.06747	0.02962	0.0574	0.04266	0.05728	0.06129	0.05174	0.05032
0.06745	0.05414	0.06238	0.05112	0.03928	0.06561	0.04512	0.05304
0.0674	0.05651	0.07721	0.05562	0.05323	0.06436	0.0558	0.07713
0.06738	0.04542	0.05847	0.06477	0.06169	0.05828	0.04577	0.05809
0.06737	0.04868	0.03598	0.05965	0.05895	0.05592	0.07621	0.06696
0.0673	0.04628	0.04486	0.07308	0.04126	0.0542	0.02991	0.04334
0.06723	0.07573	0.0784	0.07598	0.06756	0.08443	0.08072	0.0751
0.06721	0.04179	0.04668	0.06974	0.04078	0.04374	0.03072	0.0318
0.06719	0.06973	0.04562	0.06797	0.03652	0.04607	0.05544	0.04316
0.06704	0.07445	0.0718	0.06746	0.05728	0.06233	0.05654	0.05461
0.06672	0.07317	0.07744	0.06562	0.06959	0.07629	0.07153	0.08701
0.06595	0.06632	0.07578	0.07097	0.06447	0.06168	0.06798	0.07325
0.06592	0.05722	0.05165	0.05186	0.07532	0.07553	0.08819	0.07683
0.06564	0.07548	0.05353	0.06445	0.06461	0.06426	0.06202	0.06578
0.06561	0.06978	0.06487	0.06448	0.06749	0.06679	0.06962	0.07013
0.06535	0.05812	0.06962	0.05094	0.06907	0.06488	0.06275	0.06559
0.06516	0.06049	0.05458	0.0513	0.04823	0.05309	0.07242	0.05711
0.06491	0.05719	0.05924	0.06249	0.05635	0.05765	0.06044	0.06078
0.06478	0.05289	0.05693	0.07337	0.05553	0.06317	0.05891	0.02482
0.06475	0.06458	0.05585	0.07089	0.05552	0.0523	0.05559	0.05228
0.06466	0.05683	0.06496	0.05764	0.07082	0.06804	0.05749	0.06016
0.06465	0.04351	0.04586	0.06435	0.04344	0.04776	0.0384	0.04495
0.06464	0.09193	0.06889	0.05409	0.06228	0.05934	0.06908	0.07657
0.0646	0.06378	0.06301	0.05961	0.03999	0.05183	0.07865	0.05791
0.06459	0.05159	0.05707	0.0578	0.0626	0.0524	0.05436	0.05924
0.06449	0.0682	0.05853	0.08845	0.07594	0.06882	0.07499	0.08859
0.06436	0.05161	0.05601	0.07804	0.04903	0.0471	0.04165	0.03994
0.06423	0.06046	0.05952	0.06933	0.05752	0.06488	0.05202	0.04277
0.06419	0.04287	0.05423	0.07413	0.04306	0.04349	0.03654	0.04791
0.06418	0.04883	0.05155	0.06379	0.04024	0.04834	0.02249	0.03127
0.06385	0.04676	0.07093	0.06786	0.06655	0.05958	0.06237	0.06545
0.06356	0.06348	0.06589	0.06747	0.07321	0.0698	0.06567	0.06127
0.06355	0.05474	0.06143	0.05414	0.07868	0.06341	0.06158	0.06721
0.06351	0.063	0.06958	0.04811	0.06566	0.06907	0.07442	0.06924
0.06344	0.06397	0.06556	0.04584	0.05893	0.05682	0.07176	0.05876
0.0634	0.05927	0.03506	0.05551	0.03592	0.04326	0.03663	0.0397
0.06339	0.06165	0.05111	0.05811	0.03786	0.05744	0.07603	0.05784
0.06321	0.07046	0.08888	0.04938	0.07561	0.0654	0.06171	0.07697
0.06313	0.05783	0.0577	0.05587	0.08099	0.07028	0.07845	0.0677
0.06305	0.07858	0.1148	0.06528	0.06752	0.07996	0.09082	0.08338
0.06304	0.07385	0.05806	0.05276	0.06355	0.0611	0.07605	0.05786
0.06297	0.06032	0.05753	0.07921	0.05764	0.04835	0.04761	0.04855
0.06288	0.06182	0.05968	0.05038	0.06379	0.05725	0.04605	0.0402

0.06285	0.07056	0.06679	0.0748	0.07155	0.0658	0.0716	0.06179
0.06284	0.0665	0.05289	0.04341	0.05876	0.06085	0.06656	0.07115
0.0627	0.07959	0.08455	0.07037	0.07222	0.07679	0.10452	0.08686
0.06248	0.06461	0.02176	0.02174	0.03073	0.04291	0.05563	0.07673
0.06226	0.05346	0.03166	0.07872	0.04333	0.06156	0.08029	0.08286
0.06223	0.05901	0.06249	0.0506	0.05281	0.06323	0.05615	0.04831
0.06218	0.06643	0.06258	0.06364	0.06493	0.06272	0.03812	0.0342
0.06216	0.0733	0.07848	0.06091	0.07022	0.07849	0.06967	0.07776
0.06213	0.04909	0.04786	0.03023	0.05302	0.04943	0.05916	0.06288
0.06204	0.05572	0.06888	0.04789	0.01782	0.07725	0.04101	0.07525
0.06194	0.05108	0.04922	0.0602	0.01492	0.05433	0.04853	0.04509
0.06191	0.06248	0.07157	0.06137	0.06632	0.0754	0.08515	0.0599
0.06168	0.06029	0.05855	0.06448	0.05971	0.06032	0.05574	0.06234
0.06146	0.05561	0.06899	0.06321	0.07015	0.06717	0.06985	0.05967
0.0613	0.05098	0.05424	0.05158	0.05422	0.04331	0.05803	0.0531
0.06118	0.07001	0.08029	0.07411	0.06793	0.06542	0.06396	0.06331
0.06088	0.07123	0.06028	0.06354	0.06047	0.05915	0.06209	0.05578
0.06088	0.04632	0.05111	0.0531	0.0409	0.04433	0.05511	0.05544
0.06087	0.05223	0.05598	0.05415	0.05239	0.06243	0.0735	0.05869
0.06083	0.07665	0.05227	0.04129	0.05855	0.06133	0.06785	0.05742
0.0608	0.08473	0.08289	0.08556	0.06265	0.07421	0.08597	0.09628
0.06067	0.05662	0.04771	0.0651	0.0448	0.05377	0.06505	0.0426
0.06059	0.05827	0.04357	0.04064	0.06078	0.06386	0.07072	0.06446
0.06048	0.08401	0.08636	0.0746	0.07281	0.08674	0.09571	0.10726
0.06016	0.04712	0.04948	0.06591	0.04812	0.04422	0.03013	0.03395
0.06003	0.05062	0.05413	0.0544	0.05491	0.06079	0.06004	0.06116
0.05998	0.07265	0.08617	0.08121	0.06162	0.06646	0.07744	0.0517
0.05977	0.04833	0.05853	0.05076	0.05149	0.05336	0.05747	0.06791
0.05975	0.04983	0.05125	0.04477	0.04142	0.06228	0.0351	0.05816
0.05949	0.06134	0.04786	0.05684	0.04677	0.04412	0.04563	0.05403
0.05946	0.01607	0.09714	0.01416	0.09128	0.07896	0.08042	0.06495
0.05944	0.04986	0.0464	0.05076	0.0328	0.04314	0.03116	0.0389
0.05926	0.05359	0.06218	0.0509	0.05892	0.0515	0.06299	0.06337
0.05922	0.05567	0.05148	0.05059	0.04967	0.03799	0.04862	0.06091
0.0592	0.05257	0.05956	0.06117	0.04932	0.05142	0.0596	0.05643
0.05915	0.06892	0.0469	0.04624	0.05704	0.05775	0.07245	0.07636
0.05914	0.05428	0.04385	0.05106	0.04327	0.04806	0.04875	0.05914
0.05906	0.03987	0.03426	0.03212	0.03651	0.06393	0.06158	0.04957
0.05886	0.05221	0.01866	0.0544	0.04857	0.04195	0.05945	0.06955
0.05886	0.04349	0.05225	0.07364	0.04217	0.03912	0.02854	0.02352
0.05852	0.07451	0.0525	0.0437	0.0471	0.05697	0.04788	0.04827
0.05849	0.04606	0.06339	0.06395	0.05962	0.05636	0.0565	0.06337
0.05844	0.07476	0.08497	0.04556	0.09118	0.07446	0.11658	0.08786
0.05836	0.06405	0.05268	0.04878	0.04796	0.06537	0.08051	0.078
0.05822	0.05098	0.091	0.04469	0.06569	0.06405	0.06525	0.06189
0.05821	0.02274	0.04366	0.06849	0.03558	0.02017	0.02848	0.03475
0.05785	0.04018	0.0455	0.05528	0.05408	0.0543	0.04484	0.05161
0.0577	0.01967	0.01415	0.04334	0.0277	0.02101	0.02644	0.03223
0.05764	0.05761	0.06889	0.04385	0.05622	0.06458	0.06916	0.04917

0.05747	0.04511	0.04453	0.05988	0.04066	0.04479	0.03773	0.05431
0.05741	0.05365	0.0631	0.04858	0.05426	0.05622	0.06238	0.05152
0.05722	0.05697	0.07253	0.05078	0.07031	0.06533	0.0751	0.07152
0.05722	0.06278	0.06976	0.1167	0.07493	0.05321	0.04162	0.07005
0.05721	0.08213	0.06952	0.08725	0.08573	0.0791	0.07629	0.07282
0.05688	0.06392	0.05961	0.04165	0.055	0.05625	0.06858	0.0609
0.05684	0.0625	0.071	0.06734	0.05364	0.06572	0.05751	0.07445
0.05668	0.04153	0.03705	0.05914	0.04021	0.04137	0.03239	0.03695
0.05664	0.05915	0.06491	0.05558	0.04682	0.0526	0.05828	0.04364
0.05664	0.05738	0.06339	0.05898	0.06377	0.04727	0.05798	0.03695
0.05651	0.07443	0.05685	0.06863	0.04883	0.05497	0.07846	0.07173
0.05645	0.04254	0.04525	0.05094	0.03873	0.03859	0.03977	0.0481
0.05637	0.03368	0.03217	0.06598	0.03223	0.03439	0.0408	0.03854
0.05632	0.04856	0.05039	0.06166	0.0384	0.04066	0.03895	0.03533
0.05628	0.0615	0.05698	0.03176	0.0737	0.06882	0.0711	0.03586
0.05627	0.06513	0.05937	0.04454	0.05242	0.05028	0.08023	0.04664
0.05611	0.04376	0.05266	0.04525	0.0455	0.0457	0.04497	0.04852
0.05611	0.05314	0.06578	0.05558	0.07418	0.0645	0.05694	0.04161
0.05608	0.04343	0.04686	0.04393	0.05947	0.0577	0.06144	0.06334
0.05592	0.05862	0.0785	0.04323	0.06602	0.06068	0.05778	0.05744
0.05579	0.04943	0.03736	0.05479	0.0403	0.04542	0.04529	0.05919
0.05577	0.04805	0.05547	0.06217	0.04226	0.04951	0.04824	0.0653
0.0557	0.0624	0.05373	0.04996	0.06126	0.04908	0.04672	0.0543
0.05567	0.04525	0.03418	0.07306	0.04313	0.0509	0.04961	0.03332
0.05556	0.07215	0.05618	0.04843	0.0587	0.05304	0.05722	0.06051
0.05541	0.06028	0.06019	0.05052	0.05134	0.05941	0.07427	0.05625
0.0554	0.05205	0.0543	0.03995	0.05768	0.05834	0.05645	0.0627
0.05534	0.05529	0.04568	0.06162	0.03335	0.04586	0.06254	0.06598
0.05526	0.06987	0.05051	0.07714	0.06423	0.04897	0.0666	0.0481
0.05508	0.03296	0.03523	0.04476	0.03559	0.04256	0.03522	0.0378
0.05507	0.14178	0.01488	0.13281	0.12234	0.2145	0.01354	0.17305
0.05506	0.06745	0.06563	0.06486	0.06128	0.05761	0.04666	0.05789
0.05504	0.04007	0.04574	0.04934	0.03773	0.04855	0.05524	0.06675
0.05489	0.0543	0.05253	0.06665	0.04296	0.04573	0.0501	0.05272
0.05481	0.05569	0.06188	0.04449	0.05147	0.04711	0.0461	0.05346
0.05471	0.07068	0.09141	0.07083	0.09179	0.09186	0.08545	0.02674
0.05462	0.04001	0.04624	0.04957	0.04362	0.04493	0.03291	0.03754
0.05452	0.06145	0.05465	0.05315	0.06187	0.05944	0.06381	0.05547
0.05447	0.05376	0.0566	0.0565	0.0482	0.04267	0.03841	0.05728
0.05424	0.05205	0.05709	0.05889	0.05171	0.05427	0.05528	0.05844
0.05423	0.04496	0.02845	0.04368	0.05824	0.05643	0.07897	0.06897
0.05414	0.06415	0.07103	0.10186	0.06873	0.07982	0.05175	0.07256
0.0541	0.06301	0.04083	0.07812	0.03548	0.05024	0.05557	0.07368
0.05403	0.06883	0.08001	0.05418	0.07124	0.08013	0.06847	0.08705
0.05401	0.04634	0.04347	0.06088	0.03525	0.03306	0.0215	0.04152
0.05395	0.06194	0.08555	0.03919	0.05542	0.05987	0.07252	0.0589
0.05387	0.03848	0.05348	0.05033	0.04274	0.04632	0.03707	0.04637
0.05348	0.06048	0.05959	0.0442	0.05005	0.06427	0.05089	0.05324
0.05344	0.08153	0.05651	0.0629	0.07886	0.06644	0.04495	0.06962

0.0534	0.04603	0.05988	0.03961	0.0281	0.06921	0.03734	0.03078
0.05331	0.04203	0.04157	0.06533	0.0446	0.041	0.04376	0.04235
0.05329	0.04642	0.04871	0.05655	0.03807	0.04337	0.05512	0.0458
0.05321	0.05688	0.0656	0.05458	0.05144	0.05055	0.06435	0.05613
0.05314	0.05978	0.06797	0.04256	0.06321	0.06355	0.06475	0.04995
0.0531	0.04267	0.03067	0.04698	0.04276	0.0521	0.03691	0.0478
0.053	0.01366	0.05094	0.05503	0.02779	0.02738	0.01754	0.06634
0.05287	0.05936	0.05162	0.05155	0.05482	0.05097	0.05275	0.05107
0.05286	0.06115	0.068	0.05583	0.06437	0.06099	0.07226	0.05978
0.05285	0.06047	0.04724	0.05253	0.04669	0.04716	0.05361	0.05446
0.05271	0.04784	0.06123	0.03574	0.05844	0.04547	0.03321	0.06785
0.05266	0.04895	0.0606	0.11479	0.05637	0.04831	0.04582	0.04804
0.05244	0.04708	0.06373	0.04642	0.06566	0.06427	0.05881	0.06216
0.05239	0.03546	0.03258	0.04325	0.03587	0.03625	0.03672	0.03813
0.05212	0.05339	0.03028	0.01864	0.03915	0.03183	0.04416	0.01146
0.052	0.05677	0.05207	0.04953	0.05559	0.05354	0.05346	0.05545
0.05198	0.05368	0.0578	0.04749	0.05082	0.05081	0.05958	0.04949
0.05197	0.05603	0.06555	0.06643	0.05614	0.06647	0.05517	0.03274
0.05193	0.04736	0.05646	0.03669	0.06165	0.05875	0.05071	0.05938
0.05183	0.04751	0.06214	0.03853	0.06448	0.05648	0.04703	0.06506
0.05181	0.06759	0.07131	0.07241	0.05474	0.06274	0.05754	0.06021
0.05175	0.04461	0.03686	0.04716	0.0453	0.04405	0.03658	0.0545
0.0517	0.05656	0.07116	0.05017	0.05052	0.05107	0.05544	0.04938
0.05151	0.05595	0.05575	0.04198	0.05445	0.04282	0.04901	0.05185
0.05141	0.04061	0.04611	0.04368	0.03538	0.03893	0.03785	0.04663
0.05135	0.07831	0.05415	0.04542	0.05869	0.06878	0.0765	0.05758
0.05134	0.04127	0.0557	0.0649	0.04478	0.04415	0.03083	0.04833
0.05125	0.0554	0.05136	0.05601	0.05404	0.05043	0.05451	0.0472
0.0511	0.02551	0.04075	0.03195	0.04374	0.0504	0.03791	0.0463
0.05098	0.05018	0.04459	0.04625	0.04904	0.03687	0.07287	0.05212
0.05094	0.0459	0.05472	0.05656	0.04502	0.05025	0.03478	0.04576
0.0509	0.03011	0.04815	0.0486	0.0477	0.0631	0.05795	0.05242
0.0509	0.04644	0.05108	0.03866	0.0327	0.039	0.05571	0.03718
0.05086	0.04075	0.05525	0.0574	0.05494	0.05593	0.04572	0.06122
0.05081	0.02764	0.03371	0.05131	0.03156	0.03444	0.03125	0.03536
0.05079	0.0561	0.05245	0.03935	0.04851	0.05509	0.06416	0.05178
0.05072	0.05486	0.05035	0.04766	0.07028	0.06259	0.06033	0.06621
0.05072	0.05093	0.04038	0.04117	0.04235	0.0633	0.04807	0.03121
0.05067	0.04232	0.05065	0.04513	0.0467	0.05487	0.05636	0.04697
0.05067	0.05269	0.05146	0.04875	0.04719	0.05935	0.04493	0.03488
0.05056	0.04232	0.05656	0.04944	0.04747	0.04653	0.04069	0.04342
0.05055	0.04881	0.02926	0.05279	0.03966	0.04358	0.02548	0.04199
0.0505	0.03619	0.04318	0.05109	0.04927	0.04087	0.03946	0.0509
0.05041	0.05212	0.09096	0.05989	0.07236	0.08308	0.05271	0.08003
0.05037	0.07061	0.11088	0.06444	0.0657	0.07573	0.09205	0.06023
0.05036	0.04893	0.04942	0.02186	0.04466	0.04539	0.04483	0.04752
0.05035	0.04681	0.05595	0.04243	0.03094	0.04301	0.05207	0.04036
0.05032	0.06164	0.05186	0.05537	0.05487	0.06021	0.04498	0.04809
0.0503	0.03074	0.02849	0.05074	0.02594	0.03435	0.03981	0.03479

0.05029	0.03493	0.05564	0.06938	0.02446	0.04749	0.0321	0.03975
0.05022	0.06746	0.0699	0.05073	0.03138	0.03556	0.04462	0.04073
0.05011	0.0539	0.03891	0.02265	0.03493	0.04807	0.03015	0.08568
0.05011	0.04343	0.04717	0.04453	0.04792	0.06045	0.05484	0.05282
0.05006	0.0468	0.06263	0.04929	0.05053	0.05006	0.05229	0.03968
0.05002	0.04647	0.05288	0.05556	0.05659	0.0508	0.05755	0.04861
0.04994	0.04596	0.05018	0.03343	0.04956	0.03895	0.04012	0.03846
0.04985	0.03454	0.04999	0.03869	0.03215	0.04165	0.0258	0.02867
0.04985	0.03486	0.04886	0.05616	0.03507	0.0333	0.01931	0.0187
0.04981	0.03277	0.05381	0.0673	0.03521	0.03495	0.04594	0.04042
0.04978	0.04529	0.06329	0.03308	0.06094	0.06036	0.07164	0.05555
0.04971	0.04701	0.05474	0.06776	0.03961	0.04733	0.07209	0.08939
0.0497	0.05105	0.03983	0.06544	0.03555	0.05287	0.05931	0.03966
0.04958	0.06868	0.04259	0.04205	0.03589	0.04518	0.04025	0.04926
0.04952	0.03854	0.05922	0.05105	0.04821	0.04231	0.04152	0.02899
0.04948	0.03781	0.0428	0.04501	0.0382	0.03919	0.03149	0.03386
0.04947	0.06315	0.0504	0.06734	0.05214	0.05079	0.04936	0.04446
0.04941	0.05341	0.07043	0.04435	0.05287	0.05154	0.05296	0.03658
0.04936	0.04635	0.04292	0.04237	0.03944	0.03915	0.04493	0.03933
0.04933	0.04243	0.03946	0.05659	0.03911	0.04585	0.04152	0.03591
0.0493	0.05193	0.05003	0.03635	0.04298	0.04857	0.03927	0.03827
0.04929	0.05893	0.05466	0.03078	0.0532	0.05543	0.073	0.05012
0.04929	0.04286	0.04448	0.03912	0.04128	0.04514	0.04461	0.04285
0.04921	0.03899	0.03938	0.04914	0.04445	0.03929	0.03549	0.04491
0.04915	0.02188	0.06214	0.0433	0.05656	0.05825	0.05079	0.06205
0.04908	0.03773	0.04557	0.04038	0.0382	0.03682	0.02992	0.03863
0.04901	0.08057	0.07639	0.04644	0.0581	0.06238	0.05898	0.0647
0.04899	0.04735	0.0528	0.05104	0.04921	0.04933	0.04336	0.04524
0.04898	0.0632	0.03525	0.01026	0.04636	0.05561	0.04693	0.05022
0.04894	0.05291	0.06122	0.02826	0.05503	0.05107	0.03768	0.05147
0.04891	0.06821	0.07016	0.03102	0.07681	0.05975	0.07683	0.07632
0.04882	0.04567	0.06682	0.04024	0.08564	0.05957	0.06351	0.06608
0.04882	0.0504	0.08553	0.06034	0.05314	0.06455	0.04929	0.05463
0.0488	0.04584	0.05825	0.03734	0.05895	0.05441	0.04808	0.03447
0.04862	0.06349	0.10275	0.06592	0.0379	0.0669	0.05214	0.04451
0.04853	0.03988	0.04535	0.05858	0.03765	0.04519	0.04153	0.04499
0.04828	0.03619	0.01944	0.03613	0.03302	0.02962	0.03889	0.04635
0.04828	0.04663	0.03405	0.03726	0.03903	0.04365	0.05806	0.04224
0.0481	0.0392	0.04597	0.03988	0.04073	0.04338	0.03735	0.0438
0.04802	0.05358	0.05355	0.04132	0.0488	0.05697	0.05755	0.04845
0.04796	0.03975	0.04153	0.04056	0.04295	0.04577	0.047	0.02481
0.04789	0.03549	0.03585	0.0455	0.03716	0.04108	0.03364	0.04296
0.04787	0.04295	0.047	0.04874	0.04181	0.04185	0.05213	0.04325
0.04785	0.04318	0.04009	0.04704	0.03272	0.04056	0.04194	0.04815
0.04784	0.03315	0.03169	0.02398	0.02799	0.04583	0.01758	0.0306
0.04783	0.04016	0.04335	0.04076	0.04035	0.04082	0.04193	0.0478
0.04779	0.05988	0.06679	0.04866	0.06002	0.06276	0.05604	0.05642
0.04764	0.03902	0.04348	0.04754	0.04017	0.04161	0.04383	0.04693
0.04763	0.04298	0.03578	0.03679	0.04904	0.04351	0.05806	0.05125

0.04753	0.05717	0.0565	0.06319	0.06651	0.05583	0.05836	0.06269
0.04751	0.05358	0.0541	0.04523	0.05007	0.04489	0.08142	0.04678
0.04739	0.05808	0.04706	0.03823	0.01911	0.0333	0.01469	0.02772
0.04728	0.04778	0.0535	0.06182	0.04969	0.05051	0.03412	0.05217
0.04724	0.06422	0.0479	0.03745	0.04757	0.05233	0.06315	0.04458
0.04704	0.04719	0.06216	0.04582	0.04343	0.05298	0.04532	0.04087
0.04686	0.04353	0.04719	0.05157	0.0412	0.03862	0.03935	0.03259
0.04683	0.04145	0.04533	0.05433	0.04288	0.04713	0.05455	0.05256
0.04669	0.04065	0.04804	0.04883	0.04022	0.03463	0.0296	0.03947
0.04658	0.05692	0.07449	0.04401	0.01983	0.02617	0.02739	0.04711
0.04658	0.0358	0.03996	0.03535	0.04098	0.04083	0.02946	0.02942
0.04652	0.0319	0.02177	0.03938	0.03199	0.03708	0.04375	0.02087
0.04646	0.04723	0.03799	0.0404	0.03606	0.03558	0.05196	0.03257
0.0463	0.0467	0.05736	0.03543	0.04725	0.05597	0.04658	0.05592
0.04629	0.04865	0.06059	0.04473	0.0433	0.06298	0.05732	0.05903
0.04628	0.04421	0.05398	0.03783	0.04461	0.04164	0.03766	0.05799
0.04621	0.01756	0.03928	0.03947	0.02598	0.01918	0.02156	0.02256
0.0462	0.07527	0.02915	0.10284	0.03654	0.04648	0.02531	0.0387
0.04619	0.02518	0.02624	0.03686	0.03338	0.03056	0.0271	0.03323
0.04611	0.0388	0.0434	0.0517	0.01979	0.03829	0.04728	0.03173
0.04602	0.05655	0.04785	0.03134	0.04406	0.04645	0.05613	0.0436
0.04594	0.06172	0.04555	0.04706	0.02894	0.04741	0.0356	0.0461
0.04594	0.04431	0.04521	0.03176	0.03848	0.03836	0.03906	0.0417
0.04575	0.03372	0.04981	0.05965	0.04805	0.04738	0.0325	0.04104
0.04569	0.04118	0.06384	0.02326	0.03626	0.03893	0.02952	0.04071
0.04566	0.02162	0.05004	0.04705	0.02445	0.02409	0.01837	0.02021
0.04543	0.05708	0.05294	0.05671	0.05369	0.05194	0.0509	0.05065
0.04541	0.0462	0.04013	0.03435	0.04759	0.05325	0.04454	0.04973
0.0454	0.04718	0.04423	0.05186	0.04481	0.04237	0.06192	0.05265
0.04533	0.05352	0.03705	0.02671	0.04267	0.04288	0.05518	0.04577
0.04523	0.04194	0.03975	0.04927	0.04845	0.04296	0.04369	0.04119
0.04523	0.03633	0.03977	0.04831	0.04403	0.04543	0.03713	0.03291
0.0451	0.02609	0.03912	0.05633	0.03451	0.04021	0.04608	0.03724
0.045	0.04107	0.04399	0.03815	0.03839	0.03561	0.0442	0.03748
0.04496	0.04549	0.04583	0.05292	0.05806	0.04936	0.06392	0.05905
0.04483	0.05006	0.03712	0.07756	0.06044	0.05974	0.04542	0.05427
0.04482	0.04133	0.0396	0.04757	0.0477	0.06851	0.04923	0.05237
0.0448	0.03592	0.01319	0.05042	0.04789	0.04636	0.05181	0.061
0.04475	0.04731	0.04518	0.03673	0.04376	0.04769	0.04088	0.04327
0.04474	0.03906	0.04083	0.05868	0.03313	0.03948	0.02554	0.03414
0.04473	0.05049	0.04215	0.03918	0.03428	0.05823	0.05388	0.06235
0.04455	0.04643	0.05036	0.03814	0.05114	0.04858	0.0529	0.0488
0.04452	0.0326	0.03479	0.04338	0.03909	0.0394	0.03583	0.04475
0.04434	0.04426	0.03203	0.04273	0.03287	0.03592	0.03462	0.03907
0.04427	0.04359	0.03842	0.05281	0.03679	0.04062	0.02471	0.03655
0.04426	0.04279	0.04995	0.0454	0.04131	0.04445	0.04363	0.03806
0.04425	0.02616	0.02897	0.06275	0.03634	0.04072	0.02613	0.02702
0.04424	0.04543	0.02999	0.05072	0.0364	0.03665	0.03379	0.03607
0.04414	0.06987	0.05561	0.02542	0.0933	0.04506	0.05083	0.04075

0.04413	0.03633	0.03313	0.043	0.03238	0.03348	0.03242	0.03596
0.04409	0.01168	0.04607	0.01237	0.05949	0.04579	0.06008	0.04158
0.04406	0.04224	0.04251	0.03911	0.03489	0.0372	0.04349	0.03887
0.04393	0.03954	0.04196	0.0426	0.02828	0.03885	0.03079	0.02846
0.04386	0.04402	0.05718	0.04189	0.04145	0.04988	0.04314	0.04484
0.04383	0.04307	0.04222	0.04233	0.03856	0.03686	0.0304	0.02748
0.04381	0.02436	0.03209	0.05114	0.03108	0.03356	0.02031	0.02706
0.04374	0.04598	0.04029	0.04801	0.05246	0.0441	0.0486	0.04473
0.04374	0.04399	0.04014	0.04151	0.03795	0.03689	0.03898	0.03699
0.04367	0.05532	0.04642	0.0643	0.03965	0.04157	0.03104	0.04566
0.04344	0.04423	0.04977	0.04775	0.05719	0.04811	0.04954	0.04324
0.04342	0.03193	0.03013	0.03772	0.02896	0.02804	0.02407	0.03588
0.04341	0.06527	0.04795	0.06369	0.04928	0.04301	0.05521	0.03144
0.04333	0.0271	0.03191	0.04496	0.03332	0.03395	0.03398	0.03279
0.04321	0.06128	0.0432	0.06362	0.05732	0.06119	0.04053	0.06965
0.04316	0.0285	0.02974	0.0591	0.03287	0.03734	0.03406	0.04519
0.04308	0.04822	0.0497	0.04793	0.04864	0.04457	0.04805	0.05109
0.04307	0.04513	0.05854	0.05339	0.05721	0.06269	0.04473	0.05384
0.04302	0.05643	0.05574	0.05155	0.05461	0.06264	0.0779	0.0543
0.04302	0.02284	0.02826	0.02304	0.01865	0.02513	0.024	0.04487
0.04291	0.05299	0.04584	0.04012	0.04856	0.05384	0.05723	0.05322
0.04291	0.03999	0.03652	0.02857	0.03679	0.02678	0.04881	0.03852
0.04288	0.04016	0.0484	0.05322	0.0545	0.04498	0.04105	0.03925
0.04284	0.04319	0.03807	0.05497	0.0657	0.04456	0.03866	0.0369
0.04283	0.04781	0.04832	0.04375	0.05708	0.05296	0.05716	0.05714
0.04277	0.05096	0.06317	0.04844	0.05375	0.05211	0.04609	0.04998
0.04272	0.02883	0.03376	0.03406	0.02984	0.03157	0.04374	0.03484
0.04266	0.03742	0.03703	0.04529	0.0383	0.04117	0.05152	0.06191
0.04265	0.00495	0.02484	0.00779	0.00681	0.00791	0.00476	0.01149
0.0426	0.01826	0.03528	0.02417	0.02855	0.01006	0.01362	0.02313
0.04249	0.05595	0.05834	0.04532	0.06238	0.05918	0.0792	0.04826
0.04249	0.00925	0.01474	0.04595	0.0339	0.02906	0.01683	0.03905
0.0424	0.05623	0.04295	0.01202	0.00779	0.09157	0.0281	0.01942
0.04235	0.04727	0.05482	0.06478	0.04375	0.04854	0.0448	0.04999
0.04234	0.03942	0.03505	0.01081	0.03216	0.05475	0.05169	0.04805
0.04233	0.05806	0.04103	0.06478	0.05396	0.04277	0.05074	0.04484
0.04229	0.0587	0.07208	0.04385	0.06637	0.04665	0.07052	0.06111
0.04227	0.05008	0.04121	0.03968	0.04454	0.04071	0.05686	0.04488
0.04022	0.03373	0.03086	0.05683	0.02513	0.03340	0.02401	0.01100
0.04191	0.02367	0.02499	0.04513	0.02877	0.02846	0.03404	0.03783
0.04174	0.03611	0.02163	0.04687	0.03285	0.04486	0.05293	0.03112
0.04171	0.03857	0.04697	0.05201	0.04905	0.04389	0.02589	0.03444
0.04168	0.04244	0.0798	0.03653	0.11086	0.09063	0.08654	0.05846
0.04165	0.03875	0.0351	0.03032	0.04345	0.05141	0.03649	0.03431
0.04165	0.04303	0.03213	0.03821	0.0367	0.03937	0.04435	0.03035
0.04155	0.04579	0.02597	0.05903	0.05484	0.04531	0.04187	0.08357
0.04148	0.03713	0.03286	0.0589	0.03953	0.03193	0.02539	0.03397
0.04142	0.03327	0.02408	0.04497	0.02281	0.02069	0.02696	0.02602
0.04132	0.0542	0.05085	0.03203	0.04713	0.04339	0.06392	0.04231

0.04132	0.03137	0.03275	0.01865	0.05081	0.03393	0.04565	0.03928
0.04128	0.03064	0.04402	0.04285	0.04721	0.03161	0.03197	0.01844
0.04119	0.02932	0.04618	0.03476	0.03644	0.03228	0.04614	0.05457
0.0411	0.03462	0.03655	0.03149	0.0355	0.04375	0.03837	0.03375
0.04109	0.04413	0.03393	0.04994	0.03935	0.04384	0.04549	0.03998
0.041	0.04951	0.0464	0.03038	0.0409	0.04182	0.04949	0.04813
0.04084	0.02606	0.03895	0.06337	0.0313	0.02971	0.02768	0.02506
0.04083	0.04716	0.0494	0.03279	0.04283	0.04541	0.04723	0.05745
0.04083	0.0356	0.03271	0.04206	0.03679	0.04262	0.04162	0.03751
0.04073	0.03807	0.05246	0.04085	0.03675	0.03513	0.03823	0.04271
0.04072	0.04159	0.06338	0.0526	0.04441	0.05022	0.03664	0.04053
0.04067	0.04401	0.04686	0.03935	0.03236	0.04529	0.04337	0.05236
0.04063	0.02913	0.02879	0.03316	0.04713	0.03736	0.02967	0.0405
0.04043	0.03784	0.04563	0.02218	0.03495	0.03851	0.04326	0.03964
0.04043	0.02146	0.01507	0.04556	0.02635	0.0359	0.04311	0.03303
0.04037	0.04259	0.04882	0.04633	0.05062	0.042	0.05286	0.05059
0.04036	0.02874	0.03238	0.02817	0.02297	0.02742	0.02136	0.0247
0.04033	0.03705	0.0332	0.06187	0.03713	0.0401	0.04189	0.04791
0.04033	0.05002	0.04987	0.03941	0.05738	0.04514	0.05068	0.03389
0.04031	0.02347	0.02681	0.0359	0.02217	0.03204	0.01775	0.02236
0.04022	0.03373	0.03087	0.05684	0.02513	0.03341	0.02401	0.011
0.04021	0.04008	0.03789	0.02018	0.04211	0.03996	0.03628	0.03398
0.0402	0.03721	0.03646	0.05226	0.02907	0.02935	0.03097	0.02426
0.04008	0.04072	0.03836	0.04627	0.05826	0.0448	0.04305	0.04571
0.03989	0.06051	0.06106	0.06891	0.0678	0.06232	0.06582	0.04046
0.03987	0.05901	0.06824	0.07157	0.05304	0.04843	0.07254	0.04091
0.03984	0.05421	0.03248	0.04282	0.02995	0.03986	0.04699	0.04496
0.03981	0.03555	0.02991	0.03056	0.027	0.03512	0.02953	0.03666
0.0398	0.03247	0.03595	0.04071	0.03935	0.04405	0.0374	0.0405
0.03977	0.03819	0.04329	0.03112	0.03767	0.03423	0.01115	0.04117
0.03975	0.04428	0.04333	0.03398	0.03869	0.03885	0.03758	0.03817
0.03973	0.03853	0.04566	0.03207	0.04191	0.04245	0.0445	0.053
0.03968	0.03865	0.03118	0.02409	0.03436	0.0392	0.04123	0.04103
0.03968	0.01916	0.03376	0.00642	0.04216	0.02089	0.01643	0.02626
0.03967	0.02405	0.01847	0.05652	0.02273	0.0173	0.0285	0.02389
0.03963	0.02643	0.03321	0.03823	0.00254	0.03115	0.02569	0.03104
0.03952	0.03838	0.03469	0.03475	0.02978	0.04532	0.04801	0.05033
0.03946	0.02435	0.02901	0.0321	0.01856	0.03704	0.02633	0.01884
0.03944	0.05015	0.05573	0.04315	0.05829	0.05835	0.0455	0.05169
0.03935	0.03282	0.0311	0.03571	0.02611	0.0348	0.02317	0.02965
0.03929	0.0227	0.03185	0.04456	0.0284	0.03203	0.03994	0.05055
0.03923	0.03514	0.03298	0.03993	0.0285	0.0298	0.0339	0.03014
0.03922	0.05569	0.03972	0.08785	0.07587	0.06197	0.05805	0.04858
0.03914	0.03026	0.02757	0.04855	0.02383	0.03397	0.019	0.02684
0.03908	0.02955	0.05274	0.02393	0.03406	0.03218	0.05445	0.03894
0.03897	0.0424	0.04689	0.04968	0.05533	0.04241	0.0296	0.0454
0.03887	0.03649	0.05362	0.03794	0.04917	0.04395	0.03542	0.04446
0.03881	0.03845	0.05327	0.0329	0.05447	0.04672	0.0648	0.0337
0.03879	0.03853	0.03198	0.04235	0.03105	0.03338	0.03696	0.03107

0.03872	0.01815	0.02446	0.04311	0.02324	0.0278	0.0233	0.02805
0.03863	0.03073	0.03584	0.04611	0.03175	0.03515	0.03456	0.04307
0.03862	0.05932	0.04836	0.03437	0.05384	0.05481	0.06193	0.05658
0.03862	0.04309	0.04755	0.03586	0.04986	0.04671	0.04898	0.03509
0.03857	0.04052	0.04493	0.05868	0.04715	0.0471	0.051	0.04797
0.03855	0.03874	0.04251	0.03064	0.03278	0.03508	0.01392	0.03895
0.03844	0.05181	0.04671	0.03283	0.04233	0.04887	0.04502	0.03459
0.03835	0.03885	0.03854	0.0304	0.03828	0.04093	0.04203	0.03965
0.03835	0.02496	0.03191	0.0397	0.02908	0.02818	0.03012	0.02357
0.03819	0.03614	0.03495	0.0383	0.03669	0.03193	0.03793	0.03232
0.03817	0.04684	0.05528	0.04214	0.04661	0.05228	0.05018	0.04774
0.0381	0.04904	0.04557	0.04659	0.06513	0.04406	0.05646	0.04343
0.03809	0.0348	0.06267	0.0218	0.05524	0.05357	0.0544	0.04119
0.03806	0.03094	0.03192	0.04925	0.04107	0.03348	0.03231	0.03583
0.03805	0.04451	0.04774	0.05171	0.04814	0.04986	0.0593	0.04136
0.03802	0.03514	0.04075	0.02846	0.03723	0.04183	0.04897	0.04375
0.03802	0.04717	0.03527	0.03046	0.03391	0.04058	0.04205	0.03128
0.03796	0.0593	0.04457	0.04954	0.05293	0.04855	0.04852	0.03909
0.03792	0.04173	0.046	0.03377	0.04214	0.05657	0.04591	0.04973
0.03784	0.05185	0.05707	0.02982	0.06492	0.06168	0.05925	0.0492
0.03784	0.04677	0.04688	0.03695	0.05187	0.0476	0.06374	0.03768
0.03784	0.0282	0.05044	0.04295	0.02786	0.02937	0.03592	0.02211
0.03783	0.04279	0.03974	0.03754	0.03216	0.03889	0.0423	0.03926
0.03782	0.05515	0.04486	0.04535	0.04516	0.04776	0.03157	0.05246
0.03775	0.02442	0.02631	0.03075	0.02297	0.0242	0.02413	0.03736
0.03775	0.02835	0.01886	0.0339	0.03517	0.0194	0.02402	0.01615
0.03769	0.05351	0.0608	0.05948	0.05636	0.05013	0.044	0.02145
0.03762	0.0372	0.03721	0.03821	0.0432	0.04505	0.03826	0.043
0.03762	0.03275	0.0359	0.03282	0.04925	0.03894	0.03052	0.03892
0.0375	0.03908	0.04317	0.0435	0.03911	0.03285	0.02173	0.03708
0.03748	0.04528	0.04361	0.03932	0.03508	0.0335	0.04102	0.04551
0.0374	0.0508	0.04394	0.03864	0.05393	0.05708	0.06809	0.06177
0.03734	0.03001	0.02332	0.01727	0.00422	0.02926	0.04534	0.03987
0.03733	0.0483	0.06044	0.04642	0.04476	0.05109	0.04948	0.08286
0.03733	0.05286	0.07033	0.04451	0.06144	0.06623	0.06863	0.05342
0.03732	0.03544	0.03354	0.02502	0.02885	0.03254	0.0224	0.03743
0.03727	0.03115	0.0309	0.02674	0.03507	0.03734	0.03118	0.0382
0.03725	0.03942	0.03589	0.03669	0.03886	0.04084	0.04271	0.03674
0.0372	0.03252	0.02669	0.02389	0.02561	0.03276	0.02112	0.02105
0.03719	0.04393	0.04688	0.02231	0.04158	0.04271	0.04682	0.0496
0.0371	0.03996	0.03955	0.02407	0.03823	0.03912	0.05596	0.04247
0.03707	0.05624	0.01503	0.01179	0.01823	0.0281	0.01773	0.0191
0.03705	0.03597	0.03962	0.03441	0.03867	0.03773	0.0445	0.043
0.03695	0.02067	0.03682	0.02545	0.02523	0.0286	0.0395	0.01714
0.03689	0.04231	0.0191	0.04602	0.0278	0.0412	0.05513	0.03076
0.03687	0.02687	0.03507	0.02415	0.0259	0.02299	0.02762	0.03998
0.03685	0.02973	0.03125	0.02667	0.04331	0.03743	0.02885	0.03368
0.03685	0.02304	0.02257	0.04063	0.01887	0.02708	0.02601	0.01468
0.03683	0.04864	0.04006	0.02868	0.04019	0.04361	0.03348	0.0412

0.03675	0.03912	0.05128	0.03406	0.05419	0.05407	0.05543	0.04262
0.03671	0.03393	0.02996	0.03329	0.03281	0.03594	0.03216	0.03007
0.03669	0.03022	0.03155	0.03607	0.03238	0.03293	0.02375	0.02936
0.03662	0.02978	0.03405	0.06134	0.03393	0.03596	0.03269	0.04109
0.03659	0.05022	0.03117	0.0614	0.11741	0.04842	0.01926	0.05022
0.03657	0.01919	0.04542	0.01921	0.03306	0.03065	0.04256	0.02556
0.03655	0.04958	0.03678	0.03846	0.04589	0.04754	0.02557	0.03772
0.03651	0.04707	0.0533	0.02458	0.05289	0.04913	0.05659	0.04532
0.03648	0.046	0.03164	0.01525	0.01136	0.05315	0.05497	0.04361
0.03641	0.02491	0.02675	0.04218	0.02828	0.02836	0.01989	0.03142
0.0363	0.02492	0.03007	0.04353	0.03717	0.02843	0.04058	0.04189
0.03629	0.02911	0.02969	0.0287	0.02474	0.0271	0.03597	0.02414
0.03626	0.02722	0.03383	0.04064	0.03182	0.03309	0.01625	0.03649
0.03624	0.05701	0.03465	0.03737	0.03836	0.04098	0.05347	0.03935
0.03616	0.03713	0.03372	0.06442	0.07116	0.05278	0.03328	0.04032
0.03611	0.04728	0.05528	0.06537	0.08532	0.06849	0.04003	0.0564
0.03611	0.05106	0.05226	0.04153	0.06343	0.05335	0.04514	0.04724
0.0361	0.04216	0.05015	0.04982	0.0467	0.05981	0.04745	0.05421
0.03606	0.05172	0.05037	0.05071	0.03225	0.03553	0.04267	0.05021
0.03605	0.04339	0.03821	0.03405	0.03799	0.04523	0.03316	0.05521
0.03587	0.03838	0.04638	0.04311	0.04403	0.0446	0.0418	0.02791
0.03586	0.02083	0.02639	0.00527	0.01826	0.01863	0.01515	0.00567
0.03583	0.0247	0.02389	0.03826	0.02107	0.02386	0.02766	0.03088
0.03579	0.03245	0.05665	0.03119	0.03835	0.03465	0.01997	0.03601
0.03573	0.03841	0.03625	0.04232	0.03451	0.03844	0.03226	0.03457
0.03572	0.02888	0.01497	0.03604	0.02627	0.03279	0.03634	0.03153
0.03569	0.02595	0.01751	0.0316	0.02088	0.01821	0.01584	0.01276
0.03561	0.02124	0.05258	0.04607	0.05291	0.03927	0.03843	0.03075
0.0356	0.03864	0.03466	0.0291	0.0354	0.03709	0.03747	0.03725
0.03553	0.03418	0.03447	0.03653	0.03679	0.03532	0.03991	0.03795
0.03544	0.0232	0.03061	0.02031	0.02696	0.03187	0.04305	0.03577
0.03543	0.03109	0.06388	0.03617	0.02911	0.04088	0.04408	0.04203
0.03543	0.02742	0.03116	0.01329	0.04593	0.04667	0.0509	0.0397
0.03538	0.04705	0.05807	0.02745	0.06049	0.0431	0.07017	0.06375
0.03537	0.04928	0.03871	0.03726	0.04871	0.04511	0.05703	0.02927
0.03534	0.03731	0.05625	0.04682	0.05065	0.05026	0.06362	0.04277
0.03534	0.03739	0.03614	0.02987	0.03826	0.04142	0.05699	0.03348
0.03532	0.0335	0.03626	0.04404	0.04628	0.03258	0.04301	0.03704
0.03526	0.04722	0.07115	0.06641	0.07269	0.06023	0.04122	0.03818
0.03521	0.02073	0.03588	0.03613	0.03038	0.04028	0.02677	0.0195
0.03518	0.02338	0.02929	0.02685	0.02843	0.02715	0.02204	0.02691
0.03516	0.0329	0.03382	0.03534	0.04185	0.04026	0.04624	0.04239
0.03513	0.02452	0.04213	0.04947	0.02397	0.03126	0.0233	0.0474
0.03512	0.04028	0.04533	0.04954	0.041	0.03481	0.04362	0.02417
0.03506	0.024	0.0098	0.02582	0.02841	0.03693	0.0474	0.04046
0.03495	0.0351	0.03631	0.03149	0.04533	0.04036	0.03979	0.04371
0.03494	0.06389	0.05029	0.0418	0.07276	0.08581	0.05496	0.04865
0.03491	0.06329	0.04115	0.05695	0.04456	0.07397	0.05283	0.05682
0.0349	0.04034	0.04541	0.05507	0.0483	0.04702	0.0293	0.06199

0.03483	0.05012	0.05581	0.0279	0.05057	0.04899	0.05084	0.05077
0.03482	0.01718	0.01859	0.00622	0.01592	0.0258	0.03977	0.00445
0.03477	0.04698	0.03383	0.02397	0.03965	0.03833	0.02664	0.04946
0.03465	0.03379	0.04445	0.0359	0.03949	0.04308	0.0368	0.01888
0.03462	0.03107	0.02826	0.01247	0.02771	0.02999	0.02854	0.03112
0.03458	0.03814	0.04928	0.03206	0.03753	0.03605	0.04108	0.03992
0.03453	0.03954	0.02952	0.03231	0.03064	0.03423	0.04251	0.02399
0.03442	0.05493	0.03645	0.04702	0.03343	0.05027	0.06225	0.04773
0.03434	0.02774	0.0279	0.03115	0.0292	0.02575	0.02668	0.03582
0.03429	0.0607	0.06778	0.03385	0.05709	0.04834	0.05282	0.05571
0.0342	0.0323	0.04101	0.02814	0.04022	0.03201	0.03913	0.02651
0.03416	0.0385	0.05524	0.05582	0.04732	0.04557	0.05126	0.04609
0.03416	0.02218	0.03104	0.04027	0.02294	0.02376	0.04005	0.03155
0.03411	0.03075	0.04527	0.02821	0.04851	0.05449	0.03947	0.03818
0.03409	0.087	0.07763	0.05246	0.0329	0.02883	0.01081	0.24809
0.03408	0.02985	0.07169	0.01896	0.05395	0.04183	0.05229	0.03598
0.03407	0.04692	0.02778	0.11389	0.07846	0.06142	0.06669	0.03415
0.03405	0.02092	0.02469	0.03361	0.02459	0.02343	0.02575	0.01832
0.03404	0.03547	0.04282	0.03926	0.04507	0.03923	0.05737	0.04544
0.03403	0.0392	0.04302	0.04004	0.03675	0.04007	0.03651	0.02702
0.03398	0.04599	0.044	0.03891	0.04615	0.03634	0.03619	0.05396
0.03393	0.033	0.03878	0.03726	0.03501	0.03232	0.03308	0.03876
0.03393	0.02856	0.03423	0.04232	0.02982	0.03029	0.02413	0.03129
0.03392	0.0186	0.02576	0.0361	0.02854	0.0305	0.02551	0.02586
0.03391	0.03354	0.03582	0.03752	0.03938	0.04868	0.03102	0.03041
0.0338	0.01823	0.0239	0.02841	0.01654	0.01524	0.01815	0.02863
0.03373	0.0352	0.01281	0.03696	0.02675	0.03943	0.04364	0.02892
0.0337	0.02606	0.04126	0.0336	0.03276	0.05284	0.03256	0.02259
0.03367	0.02635	0.03738	0.05045	0.03915	0.02892	0.04306	0.01873
0.03364	0.01574	0.02713	0.03265	0.01813	0.0173	0.01089	0.01477
0.03362	0.04605	0.04509	0.01657	0.04624	0.05074	0.06566	0.05687
0.03361	0.02306	0.02525	0.02876	0.03512	0.02942	0.02115	0.02821
0.0336	0.02802	0.03279	0.01631	0.03004	0.02272	0.03387	0.02978
0.03359	0.03134	0.03987	0.0243	0.04013	0.02827	0.01799	0.03806
0.03352	0.02148	0.02636	0.02779	0.02549	0.02806	0.02981	0.04375
0.03352	0.04183	0.0328	0.01447	0.02841	0.03282	0.03926	0.03821
0.03351	0.05364	0.04303	0.03374	0.03781	0.03466	0.04386	0.0284
0.03346	0.02715	0.018	0.02849	0.02046	0.02509	0.01581	0.02513
0.03334	0.03243	0.02289	0.02456	0.04185	0.03677	0.05038	0.03782
0.03333	0.03803	0.03354	0.03675	0.03331	0.04915	0.03391	0.07365
0.03328	0.03598	0.05245	0.03865	0.05826	0.05013	0.05121	0.0433
0.03322	0.04859	0.03727	0.02716	0.06042	0.06144	0.07649	0.04477
0.03322	0.03723	0.03985	0.05312	0.03479	0.03664	0.03293	0.0295
0.03319	0.03357	0.02692	0.02821	0.02823	0.033	0.0449	0.02489
0.03284	0.02754	0.05745	0.04287	0.04333	0.03712	0.02955	0.03498
0.03279	0.03732	0.06064	0.0368	0.03603	0.03617	0.04668	0.04096
0.03273	0.02268	0.02615	0.0335	0.01948	0.02207	0.01806	0.01893
0.0327	0.02125	0.03031	0.03798	0.01662	0.02992	0.03192	0.02618
0.03268	0.02933	0.0319	0.03181	0.02649	0.03037	0.03245	0.03559

0.03265	0.03521	0.03571	0.0227	0.03373	0.03612	0.04681	0.03895
0.03253	0.03301	0.03448	0.0246	0.02434	0.03249	0.02648	0.02246
0.03243	0.04843	0.01368	0.05942	0.04508	0.01619	0.00734	0.00931
0.03242	0.03702	0.04647	0.03122	0.03869	0.03721	0.03656	0.0293
0.03237	0.02569	0.05028	0.03734	0.04534	0.03532	0.03304	0.02718
0.03234	0.02433	0.03372	0.0387	0.03335	0.02157	0.02264	0.03179
0.03233	0.0343	0.03448	0.02827	0.03308	0.02875	0.04036	0.02881
0.03229	0.03956	0.03466	0.03245	0.03827	0.03355	0.04152	0.03173
0.03225	0.03729	0.033	0.01938	0.03505	0.03273	0.03874	0.03079
0.03221	0.03406	0.04551	0.03175	0.04533	0.04173	0.04378	0.04682
0.03218	0.03397	0.03887	0.03313	0.03159	0.03052	0.0228	0.01195
0.03209	0.02884	0.03983	0.02247	0.03273	0.03467	0.04158	0.03863
0.03198	0.02998	0.03533	0.02364	0.03552	0.03709	0.04321	0.02934
0.03193	0.02168	0.02691	0.02747	0.03229	0.0288	0.0187	0.02902
0.03192	0.03496	0.03383	0.04657	0.02901	0.03699	0.03281	0.03926
0.03192	0.02315	0.02617	0.05229	0.01655	0.02652	0.00764	0.02668
0.03189	0.02368	0.02949	0.03538	0.03922	0.03165	0.03773	0.03898
0.03187	0.02297	0.02417	0.02905	0.02353	0.02619	0.03085	0.02319
0.03178	0.04298	0.05034	0.04052	0.03756	0.03982	0.04007	0.03241
0.03176	0.02559	0.03407	0.03256	0.03245	0.02533	0.03345	0.03324
0.03174	0.03416	0.02778	0.02585	0.03742	0.02989	0.04732	0.03687
0.03174	0.0219	0.02575	0.03889	0.03127	0.02594	0.02587	0.0349
0.03169	0.03511	0.03629	0.02386	0.04086	0.03158	0.02717	0.03468
0.03167	0.03515	0.02614	0.0259	0.02735	0.02836	0.03305	0.02902
0.03155	0.03174	0.03311	0.03746	0.03927	0.03453	0.03104	0.03164
0.03154	0.02256	0.023	0.02669	0.01947	0.02249	0.03684	0.01636
0.03151	0.02457	0.0195	0.03968	0.03504	0.04217	0.03536	0.03166
0.0315	0.02645	0.03768	0.01271	0.03646	0.03611	0.04008	0.05374
0.03148	0.01225	0.01708	0.01852	0.0146	0.01913	0.0094	0.01918
0.03147	0.03182	0.03166	0.03034	0.03243	0.02934	0.03562	0.03225
0.03144	0.01813	0.02603	0.03295	0.03657	0.02206	0.02416	0.02282
0.03136	0.02673	0.02728	0.04016	0.02441	0.02584	0.02368	0.03409
0.03131	0.03071	0.0479	0.0231	0.04023	0.0322	0.04434	0.0416
0.03128	0.0283	0.03752	0.04312	0.03229	0.03249	0.04715	0.04036
0.03128	0.02323	0.02568	0.02669	0.02695	0.02231	0.0227	0.01923
0.03122	0.04157	0.04896	0.02789	0.04087	0.04946	0.05878	0.04675
0.03122	0.03408	0.03024	0.04248	0.02518	0.033	0.04973	0.03935
0.0312	0.02873	0.02535	0.03229	0.01874	0.03033	0.04179	0.0446
0.03113	0.02906	0.02523	0.03348	0.02712	0.0398	0.04014	0.03537
0.03111	0.03356	0.03912	0.03798	0.03177	0.036	0.0256	0.03477
0.03111	0.03524	0.01575	0.02757	0.03023	0.02831	0.01977	0.02064
0.03102	0.03756	0.0295	0.05641	0.03541	0.03515	0.04728	0.04322
0.03093	0.02884	0.04153	0.05261	0.0383	0.0355	0.03323	0.03311
0.0309	0.02935	0.03038	0.02835	0.03123	0.03102	0.04146	0.01846
0.03089	0.03571	0.0339	0.03556	0.02664	0.0305	0.02422	0.02543
0.03073	0.03192	0.02369	0.01321	0.01296	0.01684	0.02452	0.03212
0.03072	0.03577	0.01783	0.05472	0.04244	0.02079	0.01336	0.01842
0.03065	0.04877	0.02588	0.02945	0.02564	0.02508	0.02148	0.0415
0.03062	0.03733	0.03583	0.04208	0.03656	0.03942	0.03055	0.03162

0.03059	0.02043	0.04281	0.04508	0.03751	0.05165	0.04649	0.02297
0.03053	0.03482	0.0315	0.01956	0.03608	0.02723	0.03224	0.02869
0.03051	0.02702	0.03049	0.0332	0.02796	0.0277	0.02514	0.02887
0.0305	0.02592	0.02748	0.01313	0.03092	0.02443	0.03231	0.03164
0.0305	0.04503	0.0492	0.0497	0.0238	0.04093	0.02833	0.03057
0.0305	0.01033	0.03227	0.00896	0.01633	0.01282	0.01535	0.00833
0.03044	0.02591	0.02594	0.0488	0.03034	0.02461	0.02613	0.03549
0.03044	0.01942	0.01356	0.01909	0.02059	0.01349	0.01744	0.017
0.0304	0.0259	0.03562	0.01706	0.0275	0.02903	0.04288	0.04196
0.03039	0.02223	0.02264	0.02601	0.02245	0.02462	0.02248	0.01857
0.03032	0.03157	0.02737	0.03657	0.04161	0.01483	0.03684	0.01609
0.03027	0.01019	0.01338	0.00573	0.0278	0.01025	0.01204	0.0093
0.03025	0.03532	0.04273	0.0373	0.03531	0.03313	0.04523	0.03482
0.03023	0.02493	0.0255	0.01125	0.02664	0.02507	0.0114	0.0285
0.03021	0.02577	0.02363	0.00776	0.02606	0.02092	0.01634	0.01679
0.03019	0.0363	0.02927	0.04538	0.02124	0.02312	0.03486	0.03205
0.03017	0.01947	0.02856	0.01906	0.01866	0.02973	0.01394	0.01604
0.03012	0.02313	0.02386	0.03582	0.01777	0.01823	0.00784	0.02591
0.0301	0.01758	0.02136	0.03411	0.02515	0.02561	0.02341	0.03374
0.03006	0.01636	0.01674	0.03412	0.01132	0.02293	0.00788	0.01107
0.02997	0.02919	0.02697	0.03192	0.02651	0.02482	0.02773	0.03181
0.02997	0.02547	0.02696	0.03651	0.02864	0.02841	0.02593	0.02486
0.02996	0.03473	0.03233	0.04445	0.0456	0.03679	0.03399	0.04647
0.02992	0.03047	0.04108	0.01268	0.02633	0.0353	0.02966	0.06063
0.02978	0.03611	0.04329	0.03841	0.05118	0.05068	0.04854	0.04399
0.02976	0.0162	0.02668	0.01495	0.02567	0.01939	0.03639	0.0271
0.02962	0.02913	0.05238	0.03953	0.03865	0.03393	0.02304	0.03146
0.02959	0.0289	0.02447	0.00822	0.02708	0.02441	0.04267	0.02596
0.02956	0.01577	0.02484	0.00699	0.02421	0.02242	0.0096	0.01051
0.02955	0.04001	0.03674	0.029	0.02039	0.03112	0.02395	0.02466
0.02955	0.02838	0.02609	0.02487	0.02696	0.02441	0.02221	0.01648
0.02954	0.02942	0.03908	0.02788	0.0294	0.05155	0.02571	0.032
0.02953	0.02714	0.03607	0.02722	0.03453	0.03612	0.02639	0.02637
0.02952	0.02672	0.03018	0.03061	0.0261	0.02253	0.02018	0.02663
0.02951	0.03332	0.03322	0.02524	0.03466	0.03465	0.03667	0.0298
0.02945	0.02492	0.02366	0.01648	0.02286	0.02178	0.01928	0.0182
0.02942	0.03326	0.03194	0.02561	0.03519	0.02825	0.03505	0.05403
0.0294	0.03073	0.03212	0.03024	0.03745	0.03456	0.01964	0.03389
0.02937	0.02234	0.02465	0.02442	0.0237	0.02041	0.02737	0.02513
0.02933	0.02509	0.03162	0.00476	0.03054	0.03013	0.03073	0.03334
0.02926	0.02175	0.03497	0.03375	0.02751	0.02527	0.01247	0.02873
0.02914	0.02798	0.02506	0.0493	0.04194	0.04055	0.02621	0.0334
0.02907	0.02666	0.02139	0.02232	0.02428	0.0227	0.02839	0.03018
0.02897	0.02851	0.03871	0.03072	0.03875	0.03464	0.03722	0.02561
0.02893	0.01939	0.02596	0.01555	0.0407	0.02202	0.02795	0.03144
0.02884	0.02054	0.027	0.02086	0.02615	0.02627	0.02234	0.02442
0.0288	0.03177	0.03851	0.02658	0.03207	0.04003	0.04303	0.04019
0.0288	0.02272	0.02589	0.03793	0.03262	0.02878	0.03501	0.0292
0.02876	0.03231	0.01746	0.00272	0.01841	0.00968	0.02427	0.01425

0.02874	0.03345	0.02579	0.02262	0.038	0.03621	0.04223	0.0344
0.02868	0.03351	0.0297	0.02732	0.03232	0.02651	0.03229	0.03706
0.02866	0.03346	0.03066	0.03554	0.03503	0.03272	0.03441	0.0353
0.02863	0.05499	0.06721	0.03179	0.04843	0.05055	0.05045	0.01834
0.02861	0.03218	0.01408	0.08326	0.06482	0.0183	0.00377	0.0054
0.02858	0.02928	0.02899	0.00603	0.03743	0.03779	0.03897	0.03685
0.02857	0.03005	0.04148	0.0249	0.03998	0.04721	0.03376	0.03525
0.02857	0.02129	0.03112	0.0299	0.02884	0.02577	0.02287	0.02288
0.02855	0.03294	0.03509	0.03962	0.03265	0.03689	0.03882	0.03105
0.02854	0.05748	0.06173	0.03979	0.0505	0.05261	0.05051	0.03474
0.02853	0.02563	0.03606	0.02926	0.03699	0.0349	0.02976	0.03696
0.02852	0.0355	0.03972	0.02649	0.04192	0.04852	0.05192	0.03072
0.02846	0.02269	0.02715	0.02594	0.0217	0.02219	0.01621	0.0181
0.0284	0.02465	0.02519	0.03242	0.0234	0.02265	0.01161	0.0226
0.02831	0.03769	0.0382	0.01888	0.0242	0.02842	0.02149	0.01937
0.02829	0.02909	0.0273	0.04072	0.03115	0.03139	0.02911	0.02125
0.02826	0.03276	0.0347	0.03075	0.0268	0.03157	0.04662	0.03354
0.02825	0.02266	0.02964	0.0315	0.02274	0.03122	0.02027	0.02629
0.02822	0.02999	0.036	0.02104	0.03916	0.02111	0.0186	0.01997
0.02822	0.02123	0.03341	0.0278	0.02719	0.03214	0.01852	0.01343
0.02819	0.03678	0.04171	0.02507	0.03357	0.04625	0.05681	0.03968
0.02817	0.01642	0.02772	0.02186	0.02677	0.03241	0.03085	0.02618
0.02816	0.0347	0.03381	0.02718	0.03518	0.03899	0.04284	0.03784
0.02814	0.03979	0.04561	0.04183	0.05485	0.04331	0.03663	0.04397
0.02814	0.02017	0.02347	0.0208	0.0233	0.02706	0.01847	0.02142
0.02813	0.01	0.01943	0.01314	0.01727	0.01926	0.01548	0.00858
0.02804	0.02966	0.03283	0.02634	0.04212	0.03707	0.03646	0.03756
0.02804	0.03157	0.03906	0.03145	0.02957	0.0261	0.02894	0.02846
0.02802	0.01778	0.03165	0.06497	0.02382	0.01666	0.02895	0.02271
0.02801	0.03864	0.04034	0.03173	0.03454	0.03393	0.04047	0.03057
0.02799	0.03444	0.05845	0.04078	0.05762	0.05203	0.04803	0.02903
0.02795	0.03508	0.04846	0.03483	0.04592	0.0363	0.03393	0.03419
0.02795	0.0173	0.01502	0.05319	0.02013	0.02639	0.03571	0.03112
0.02783	0.01294	0.01633	0.00864	0.01972	0.02573	0.01787	0.07186
0.02779	0.02824	0.02822	0.01932	0.03526	0.03537	0.03345	0.03294
0.02778	0.025	0.03328	0.02286	0.03127	0.03324	0.02389	0.02188
0.02774	0.03369	0.0376	0.02848	0.02685	0.03429	0.03448	0.02271
0.0277	0.01368	0.01888	0.02759	0.01877	0.00383	0.00971	0.00883
0.02764	0.02799	0.0218	0.02112	0.03581	0.03187	0.03414	0.03451
0.0276	0.01687	0.02226	0.03172	0.02111	0.02083	0.0161	0.02246
0.02756	0.03314	0.04005	0.01887	0.02769	0.02306	0.02583	0.00976
0.02753	0.0469	0.03482	0.02804	0.02866	0.02929	0.02218	0.03747
0.02752	0.01711	0.01948	0.02667	0.02284	0.02082	0.02362	0.02594
0.02747	0.05297	0.08805	0.06517	0.10492	0.10727	0.09434	0.04716
0.02747	0.03255	0.02797	0.02631	0.03147	0.02643	0.01619	0.0195
0.02746	0.03953	0.05012	0.03338	0.04145	0.04448	0.04722	0.05246
0.02735	0.03351	0.03759	0.02186	0.03976	0.03481	0.03978	0.0365
0.02733	0.02264	0.03028	0.03112	0.0259	0.02437	0.02607	0.02042
0.02732	0.02829	0.03447	0.01599	0.03347	0.0268	0.03864	0.02922

0.02729	0.00898	0.01523	0.01437	0.0258	0.02717	0.02507	0.03365
0.02729	0.0146	0.02311	0.03947	0.02656	0.0201	0.0249	0.02769
0.02724	0.03736	0.03664	0.02353	0.02147	0.03122	0.0246	0.02239
0.02723	0.04643	0.02602	0.03191	0.04426	0.07562	0.05821	0.02744
0.02714	0.03225	0.03275	0.02529	0.03525	0.02886	0.04825	0.0298
0.02714	0.01869	0.00801	0.01369	0.0199	0.01303	0.0118	0.02913
0.02714	0.01445	0.01827	0.01569	0.01102	0.01038	0.02131	0.02381
0.02711	0.0232	0.01954	0.05103	0.03945	0.03606	0.02308	0.02117
0.0271	0.02787	0.04092	0.01537	0.03884	0.04767	0.04831	0.03565
0.02707	0.01708	0.01766	0.02412	0.01678	0.01729	0.01588	0.01661
0.02706	0.03219	0.03777	0.03335	0.03772	0.03515	0.02703	0.0276
0.02705	0.04209	0.04003	0.01205	0.04261	0.04002	0.04858	0.0455
0.02705	0.02268	0.02261	0.045	0.02686	0.02316	0.02295	0.02413
0.02704	0.02716	0.02621	0.02727	0.0294	0.02564	0.02499	0.03011
0.02703	0.03924	0.03755	0.02828	0.04747	0.03813	0.0344	0.03633
0.02701	0.03033	0.03172	0.01614	0.03476	0.03328	0.03144	0.03109
0.02696	0.03555	0.04543	0.04581	0.05059	0.03888	0.03427	0.02449
0.0269	0.02748	0.03022	0.02908	0.03237	0.02992	0.01881	0.0255
0.02688	0.02134	0.01619	0.02437	0.01383	0.01577	0.03009	0.01718
0.02687	0.03324	0.03257	0.04478	0.02829	0.01949	0.0169	0.01702
0.02686	0.03135	0.02143	0.05385	0.04306	0.04164	0.03359	0.06133
0.02686	0.02576	0.02616	0.0318	0.017	0.03396	0.04566	0.02972
0.02679	0.02684	0.03205	0.02479	0.02829	0.02327	0.03168	0.02612
0.02678	0.02364	0.03176	0.02673	0.02347	0.02921	0.02198	0.03042
0.02676	0.03366	0.02605	0.01133	0.02449	0.02911	0.02906	0.02899
0.02676	0.03371	0.01669	0.03007	0.02426	0.02786	0.02405	0.02322
0.02673	0.01249	0.01965	0.05617	0.02753	0.031	0.03189	0.02663
0.02671	0.03025	0.02547	0.02371	0.03034	0.03349	0.04204	0.02442
0.02668	0.02261	0.02624	0.11232	0.04927	0.03285	0.01873	0.03031
0.02666	0.02101	0.02124	0.0345	0.02421	0.02478	0.02667	0.02044
0.02664	0.03887	0.03095	0.02208	0.03343	0.02975	0.04276	0.0303
0.02656	0.02864	0.02236	0.02346	0.0254	0.03039	0.01978	0.03016
0.02655	0.03064	0.02503	0.0234	0.02861	0.02946	0.04056	0.02255
0.02653	0.03056	0.03271	0.05387	0.02343	0.02185	0.02593	0.03213
0.02647	0.02379	0.0282	0.03184	0.02469	0.02789	0.01703	0.02618
0.02641	0.03782	0.0143	0.02795	0.02852	0.02889	0.0386	0.02408
0.0264	0.03387	0.03786	0.0232	0.04113	0.03201	0.0349	0.04304
0.02639	0.0305	0.0414	0.03551	0.0307	0.03341	0.03004	0.03767
0.02639	0.02571	0.01225	0.01508	0.02327	0.02961	0.00907	0.03001
0.02637	0.02655	0.0375	0.02845	0.02996	0.03121	0.03275	0.03039
0.02631	0.03087	0.02408	0.04979	0.04325	0.0245	0.02707	0.05215
0.02631	0.0313	0.03899	0.02094	0.02668	0.02632	0.02214	0.03448
0.02625	0.0551	0.02774	0.01599	0.07487	0.02685	0.0297	0.04304
0.02624	0.01443	0.01981	0.02062	0.02652	0.02017	0.0257	0.01556
0.0262	0.02961	0.02504	0.0191	0.02735	0.02379	0.01978	0.02719
0.0262	0.01777	0.02445	0.0255	0.02106	0.02759	0.01813	0.02458
0.02618	0.02723	0.03273	0.02099	0.03019	0.03222	0.03154	0.02215
0.02617	0.02612	0.02709	0.038	0.02836	0.02878	0.02364	0.01821
0.02617	0.02454	0.03043	0.02009	0.02295	0.023	0.02164	0.0154

0.0261	0.04764	0.04797	0.03912	0.03564	0.04575	0.03182	0.04908
0.02607	0.06368	0.00601	0.0714	0.0246	0.03949	0.01492	0.02131
0.02605	0.00901	0.00603	0.02627	0.00579	0.00334	0.00703	0.00312
0.02602	0.01862	0.02166	0.01624	0.02444	0.0222	0.02718	0.02277
0.02599	0.02675	0.02348	0.02775	0.02909	0.02491	0.05376	0.02916
0.02597	0.02267	0.0227	0.03133	0.02071	0.02304	0.02526	0.02087
0.02593	0.03456	0.03012	0.02269	0.03258	0.03043	0.04067	0.02849
0.02593	0.02327	0.03203	0.02989	0.02813	0.03192	0.02372	0.02667
0.02591	0.01464	0.02329	0.02367	0.02219	0.02269	0.01779	0.01932
0.02587	0.01957	0.01288	0.00314	0.04017	0.03883	0.03545	0.0356
0.02586	0.02153	0.01576	0.01991	0.02767	0.02607	0.03299	0.02165
0.02583	0.02461	0.02548	0.02096	0.02508	0.02624	0.02876	0.03139
0.02582	0.03656	0.02136	0.02097	0.01436	0.00804	0.00973	0.02357
0.02577	0.02134	0.02626	0.02065	0.02927	0.02351	0.01914	0.02237
0.02572	0.02317	0.02965	0.01857	0.02669	0.02552	0.03028	0.02987
0.02572	0.03866	0.05137	0.04871	0.05472	0.04492	0.04555	0.02623
0.02571	0.01529	0.0301	0.02636	0.02566	0.01451	0.03733	0.02056
0.0256	0.02898	0.02979	0.02845	0.03536	0.03439	0.02624	0.02819
0.02558	0.02038	0.0285	0.02267	0.02867	0.03054	0.02015	0.03562
0.02558	0.01983	0.02438	0.02965	0.02137	0.02113	0.02224	0.01874
0.02557	0.02958	0.02578	0.02563	0.0334	0.02711	0.02396	0.0201
0.02551	0.02889	0.02625	0.04051	0.03965	0.04425	0.04048	0.03848
0.02549	0.02108	0.02088	0.0245	0.02238	0.02829	0.03225	0.03913
0.02548	0.02336	0.02294	0.02871	0.02443	0.04496	0.0206	0.02953
0.02538	0.02655	0.03146	0.0308	0.03035	0.03172	0.02331	0.03068
0.02537	0.02077	0.02336	0.02795	0.01586	0.02268	0.02555	0.03076
0.02537	0.02476	0.0221	0.03125	0.02432	0.02395	0.02415	0.03064
0.02534	0.01671	0.02366	0.02955	0.02048	0.01972	0.01919	0.01023
0.02528	0.02112	0.01764	0.02401	0.0235	0.03071	0.01559	0.02906
0.02526	0.03883	0.04647	0.04163	0.02937	0.0403	0.03188	0.03461
0.02526	0.02382	0.02324	0.02117	0.02512	0.03065	0.01913	0.02965
0.02522	0.01186	0.0139	0.03244	0.01041	0.01177	0.01279	0.01071
0.0252	0.01629	0.02256	0.03131	0.01337	0.02127	0.01281	0.01726
0.02518	0.01622	0.019	0.02551	0.02065	0.01923	0.02059	0.01862
0.02517	0.0279	0.03074	0.01639	0.03152	0.02934	0.04	0.03269
0.02517	0.01722	0.01963	0.02076	0.01845	0.01826	0.01435	0.02062
0.02511	0.01074	0.02332	0.02167	0.03051	0.03757	0.03017	0.02877
0.02507	0.01621	0.0271	0.04708	0.02235	0.01943	0.03312	0.0225
0.02502	0.01702	0.01542	0.02664	0.01173	0.01132	0.01771	0.01455
0.025	0.03098	0.04177	0.0246	0.03315	0.04147	0.02974	0.03147
0.02498	0.01471	0.02067	0.01906	0.02715	0.02063	0.02566	0.03465
0.02498	0.01262	0.02084	0.04654	0.0106	0.01167	0.02123	0.01352
0.02497	0.01802	0.01701	0.01877	0.01599	0.0132	0.0174	0.0172
0.02494	0.02392	0.0362	0.02662	0.03203	0.03714	0.03823	0.03001
0.02494	0.01998	0.01904	0.03623	0.01964	0.01968	0.02185	0.02177
0.02492	0.0369	0.00022	0.06156	0.01443	0.03729	0.003	0.02213
0.02489	0.01802	0.02852	0.02304	0.02502	0.02487	0.01806	0.02013
0.02485	0.03298	0.03511	0.02854	0.03499	0.03475	0.03206	0.04535
0.0248	0.02284	0.03062	0.03938	0.02706	0.02558	0.03273	0.02755

0.02477	0.03709	0.02316	0.02791	0.02223	0.03203	0.02317	0.02604
0.02474	0.03894	0.0321	0.02972	0.01951	0.02047	0.02841	0.0533
0.02472	0.02074	0.02958	0.02072	0.03247	0.03217	0.02937	0.02716
0.02468	0.02446	0.03178	0.03003	0.01669	0.03172	0.02388	0.0341
0.02467	0.02453	0.02806	0.02272	0.03142	0.02981	0.02123	0.02509
0.02461	0.02871	0.05881	0.02858	0.04264	0.03651	0.04485	0.03787
0.02461	0.0228	0.01319	0.0222	0.01487	0.01559	0.01986	0.01785
0.02459	0.01607	0.045	0.02652	0.03218	0.02815	0.01893	0.02587
0.02457	0.02809	0.03753	0.02288	0.04067	0.04071	0.03835	0.0342
0.02457	0.01861	0.0184	0.02089	0.02299	0.0185	0.01552	0.0281
0.02455	0.02853	0.03896	0.03726	0.03037	0.0341	0.02445	0.02867
0.02452	0.02781	0.02721	0.02222	0.03357	0.03515	0.0212	0.03132
0.02451	0.02715	0.03499	0.01994	0.03088	0.00978	0.02845	0.03475
0.02446	0.03752	0.02911	0.21088	0.01681	0.0294	0.02703	0.05928
0.02446	0.02116	0.02734	0.03224	0.03072	0.02826	0.03227	0.02295
0.02442	0.03317	0.02976	0.02102	0.02477	0.02628	0.01618	0.02945
0.02441	0.01455	0.01598	0.01705	0.0167	0.01584	0.01634	0.02666
0.0244	0.02588	0.03205	0.02604	0.03285	0.02856	0.03438	0.03032
0.02437	0.03019	0.027	0.02598	0.02375	0.03148	0.0306	0.02951
0.02433	0.03845	0.03604	0.02162	0.04675	0.03236	0.03486	0.03858
0.02432	0.02779	0.01387	0.02394	0.01014	0.01371	0.02877	0.01875
0.02428	0.03448	0.03971	0.02152	0.03893	0.04018	0.03821	0.0413
0.02426	0.03016	0.02065	0.01882	0.01922	0.02874	0.02741	0.03008
0.02425	0.0323	0.02756	0.01883	0.02317	0.02869	0.02146	0.02712
0.02425	0.02676	0.02739	0.02776	0.028	0.03014	0.02366	0.02149
0.02424	0.01998	0.02543	0.02679	0.01919	0.02319	0.02987	0.02318
0.02423	0.02934	0.02595	0.02295	0.0248	0.02509	0.0273	0.02948
0.02421	0.02074	0.02035	0.02195	0.02038	0.02948	0.01926	0.02224
0.02419	0.0297	0.05833	0.04876	0.05754	0.04916	0.04324	0.03926
0.02418	0.02322	0.03489	0.02207	0.04326	0.03128	0.04049	0.04332
0.02418	0.01673	0.02697	0.0084	0.02997	0.03066	0.01612	0.02318
0.02411	0.02394	0.03606	0.01476	0.03384	0.03834	0.02599	0.03138
0.0241	0.02323	0.02401	0.02438	0.02737	0.03083	0.02913	0.03179
0.02407	0.01933	0.0234	0.02333	0.02972	0.03121	0.02489	0.02877
0.02403	0.02185	0.02735	0.02602	0.02501	0.01992	0.02418	0.01945
0.02402	0.01633	0.01977	0.00856	0.02394	0.02644	0.02067	0.02141
0.02402	0.01617	0.01641	0.02062	0.01657	0.02153	0.01917	0.01799
0.02395	0.04849	0.04352	0.04213	0.04018	0.04512	0.04837	0.03128
0.02395	0.01315	0.02667	0.02475	0.02021	0.02178	0.01968	0.01315
0.02391	0.02857	0.02478	0.02092	0.02026	0.02977	0.03332	0.01226
0.0239	0.01492	0.02124	0.02044	0.01708	0.02028	0.01823	0.02322
0.02385	0.04361	0.04617	0.02442	0.05768	0.03796	0.04457	0.03018
0.02384	0.01943	0.01976	0.0234	0.03418	0.03293	0.02608	0.02235
0.02381	0.01983	0.00776	0.01321	0.01348	0.00917	0.02343	0.01008
0.0238	0.0135	0.01616	0.0225	0.02212	0.01941	0.01582	0.01589
0.02371	0.01621	0.03245	0.02736	0.02484	0.02638	0.01433	0.02092
0.0237	0.02069	0.01943	0.02629	0.02486	0.02483	0.01433	0.02286
0.02366	0.02647	0.03435	0.02034	0.03607	0.03809	0.03119	0.02983
0.02364	0.01993	0.02514	0.03162	0.01928	0.0218	0.02745	0.03252

0.02358	0.02967	0.01913	0.01832	0.02448	0.0149	0.02978	0.02724
0.02356	0.0273	0.03347	0.01902	0.02289	0.01904	0.03396	0.02131
0.02353	0.03708	0.04033	0.01363	0.04368	0.04726	0.04407	0.04343
0.02352	0.01489	0.03552	0.02627	0.02702	0.02225	0.02205	0.01982
0.02351	0.03034	0.03513	0.0375	0.02759	0.02813	0.02566	0.01787
0.02351	0.01958	0.01848	0.01443	0.01536	0.01705	0.01553	0.01289
0.0235	0.01389	0.0198	0.04011	0.02482	0.02205	0.00794	0.01867
0.02348	0.01205	0.01597	0.01236	0.0142	0.0155	0.0153	0.01403
0.02339	0.01433	0.04159	0.01516	0.03199	0.02159	0.0227	0.01635
0.02334	0.02187	0.01701	0.04856	0.01989	0.02038	0.01113	0.03214
0.02327	0.0208	0.02562	0.02232	0.02178	0.02045	0.01742	0.01848
0.02324	0.0095	0.02364	0.01806	0.0182	0.02594	0.01889	0.02163
0.02315	0.02609	0.0292	0.02581	0.03072	0.02523	0.0277	0.0273
0.02311	0.03155	0.01113	0.03667	0.02796	0.0198	0.03131	0.03196
0.02311	0.01893	0.02993	0.01719	0.02031	0.0292	0.02145	0.01385
0.02309	0.02409	0.02126	0.04152	0.02344	0.02359	0.03073	0.0233
0.02307	0.01272	0.01799	0.03736	0.01765	0.02105	0.02315	0.02267
0.023	0.01953	0.03394	0.01837	0.02387	0.04246	0.01813	0.033
0.02297	0.03285	0.0285	0.03097	0.02862	0.03527	0.02731	0.03212
0.02297	0.01936	0.01841	0.02055	0.02033	0.02238	0.01945	0.01711
0.02295	0.00854	0.01753	0.02422	0.01901	0.02053	0.01939	0.00849
0.02294	0.02389	0.02313	0.02386	0.02795	0.031	0.02288	0.01373
0.02293	0.01355	0.0175	0.01773	0.03527	0.02301	0.02001	0.01833
0.02292	0.0136	0.0127	0.02842	0.01145	0.01514	0.01073	0.01618
0.02284	0.02515	0.01167	0.00321	0.03053	0.03192	0.02211	0.02099
0.0228	0.01542	0.02148	0.00666	0.02536	0.02162	0.0198	0.01494
0.02278	0.03556	0.03851	0.04532	0.02666	0.03492	0.03444	0.04523
0.02277	0.01074	0.02088	0.02692	0.02799	0.03213	0.02374	0.01739
0.02274	0.01781	0.02953	0.02328	0.01782	0.01507	0.02092	0.02062
0.02273	0.02796	0.03502	0.02052	0.03121	0.03203	0.02965	0.02223
0.02272	0.02644	0.05781	0.05984	0.08429	0.10184	0.03507	0.07959
0.02269	0.01663	0.03785	0.02139	0.03349	0.03273	0.0288	0.03052
0.02268	0.02602	0.04553	0.0492	0.03924	0.02673	0.04539	0.03561
0.02268	0.01333	0.01634	0.02289	0.01514	0.01891	0.01775	0.01546
0.02263	0.01895	0.02428	0.01452	0.02391	0.02241	0.03746	0.03226
0.02259	0.02309	0.01598	0.02705	0.01973	0.01737	0.01787	0.02933
0.02257	0.0229	0.04485	0.04174	0.02107	0.02688	0.02298	0.03976
0.02257	0.01638	0.02303	0.02503	0.02831	0.0218	0.02377	0.01749
0.02252	0.02137	0.00549	0.04405	0.0199	0.01314	0.00267	0.02168
0.0225	0.02687	0.03071	0.01788	0.02943	0.02258	0.03079	0.02802
0.02248	0.01935	0.00987	0.05822	0.0302	0.02995	0.02523	0.02054
0.02244	0.03443	0.04282	0.0354	0.03477	0.0251	0.03682	0.0273
0.02243	0.01941	0.01906	0.01961	0.02247	0.02075	0.01262	0.01705
0.02242	0.02146	0.01795	0.02668	0.02147	0.02159	0.01974	0.01883
0.0224	0.02185	0.02795	0.02617	0.02482	0.02601	0.02385	0.02894
0.02237	0.02074	0.03496	0.02351	0.02899	0.02301	0.01958	0.01524
0.02233	0.03243	0.02399	0.0078	0.05299	0.04436	0.03294	0.04143
0.02233	0.02051	0.01758	0.0111	0.01615	0.01777	0.02592	0.01962
0.0223	0.02286	0.03031	0.02103	0.02832	0.03796	0.02855	0.02568

0.02229	0.04618	0.03912	0.03395	0.04712	0.03724	0.06894	0.04314
0.02227	0.02099	0.01999	0.0177	0.01913	0.02133	0.02906	0.02799
0.02225	0.04472	0.02195	0.02343	0.01708	0.04575	0.04323	0.04277
0.02216	0.01254	0.0172	0.01795	0.02187	0.02419	0.01864	0.0181
0.02215	0.02836	0.01495	0.02321	0.03312	0.03511	0.03724	0.05364
0.02207	0.03045	0.04632	0.02935	0.02836	0.03678	0.04055	0.03239
0.02207	0.02707	0.02789	0.02651	0.02799	0.02673	0.02357	0.01989
0.02203	0.0238	0.03057	0.02531	0.03308	0.02625	0.0233	0.0262
0.02202	0.02195	0.01805	0.03463	0.02158	0.02131	0.01836	0.02236
0.02193	0.02791	0.02013	0.01872	0.0242	0.02602	0.03328	0.02077
0.0219	0.02377	0.02856	0.02319	0.02097	0.02258	0.02783	0.0205
0.02189	0.029	0.04291	0.02073	0.04759	0.05178	0.055	0.03367
0.02189	0.017	0.02356	0.00904	0.02042	0.02352	0.01955	0.02383
0.02189	0.03443	0.0254	0.03208	0.02047	0.02114	0.01976	0.01878
0.02188	0.02372	0.00012	0.03613	0	0.03137	0	0.02179
0.02187	0.02092	0.03175	0.03067	0.02305	0.01917	0.01451	0.0208
0.02184	0.02485	0.01372	0.03979	0.02641	0.02659	0.01786	0.02538
0.02182	0.03103	0.02168	0.01895	0.02043	0.01897	0.01583	0.01832
0.02177	0.01081	0.01455	0.01382	0.03082	0.02756	0.02142	0.02669
0.02177	0.0168	0.00575	0.01397	0.01969	0.02261	0.01952	0.01446
0.02176	0.02066	0.02297	0.01829	0.01843	0.01933	0.0178	0.01547
0.02175	0.02298	0.02763	0.01848	0.02859	0.03341	0.02754	0.02767
0.0217	0.01214	0.00924	0.01395	0.00985	0.01394	0.00814	0.01291
0.02169	0.0286	0.02543	0.11866	0.0471	0.05173	0.01847	0.05699
0.02169	0.0169	0.01774	0.02082	0.02348	0.02302	0.01422	0.02373
0.02167	0.0175	0.02926	0.01416	0.0034	0.03522	0.02732	0.02987
0.02167	0.0203	0.01612	0.02058	0.0176	0.02253	0.02146	0.02344
0.02166	0.02903	0.02794	0.0095	0.03609	0.02983	0.02897	0.03009
0.02165	0.01751	0.02126	0.02387	0.0191	0.01897	0.02275	0.03081
0.02165	0.0242	0.02942	0.02239	0.02816	0.02889	0.01685	0.02634
0.02161	0.02652	0.00845	0.01173	0.01842	0.0313	0.0126	0.00602
0.0216	0.01912	0.01974	0.01743	0.02491	0.02884	0.02388	0.02856
0.02154	0.02229	0.02251	0.02159	0.03328	0.02338	0.02096	0.02686
0.02149	0.01191	0.02121	0.01234	0.02031	0.0231	0.02127	0.02051
0.02147	0.01488	0.01403	0.02453	0.01663	0.01456	0.01205	0.01005
0.02146	0.02871	0.03723	0.02014	0.03135	0.03446	0.03282	0.03704
0.02146	0.01547	0.01202	0.01273	0.02426	0.01583	0.00622	0.01148
0.02145	0.01993	0.01494	0.02137	0.01594	0.01279	0.01458	0.01835
0.0214	0.02421	0.03244	0.03191	0.03143	0.02826	0.02181	0.03356
0.02137	0.02847	0.02098	0.01422	0.02656	0.03354	0.02763	0.02206
0.02137	0.01445	0.02502	0.00659	0.02553	0.02988	0.02429	0.01921
0.02136	0.02366	0.02081	0.02912	0.02442	0.0173	0.02342	0.01366
0.02135	0.0256	0.0345	0.03501	0.03403	0.03266	0.03029	0.02741
0.02133	0.02184	0.02533	0.02339	0.02369	0.02801	0.02079	0.02988
0.02129	0.02867	0.03517	0.02861	0.02991	0.03455	0.02377	0.04555
0.02129	0.00149	0.0143	0.04159	0.01473	0.01406	0.00886	0.01514
0.02128	0.02405	0.02463	0.02435	0.02567	0.02164	0.02991	0.0226
0.02126	0.04738	0.0135	0.04647	0.02975	0.03285	0.02466	0.0121
0.02125	0.01734	0.03419	0.01602	0.0493	0.03875	0.03922	0.03628

0.02122	0.01212	0.01893	0.01032	0.02123	0.02601	0.02629	0.02351
0.02119	0.01879	0.02406	0.02072	0.01758	0.0216	0.01948	0.02425
0.02112	0.04101	0.0285	0.07103	0.03012	0.03921	0.03501	0.03608
0.02112	0.0242	0.02031	0.01269	0.01978	0.01029	0.02266	0.00189
0.0211	0.02586	0.02286	0.00817	0.05204	0.02123	0.02089	0.03081
0.02109	0.01422	0.02754	0.01859	0.0284	0.02657	0.0356	0.01807
0.021	0.00986	0.01278	0.01574	0.01957	0.02058	0.01559	0.0249
0.021	0.02486	0.02345	0.01594	0.02697	0.02984	0.01622	0.01792
0.02094	0.01839	0.01913	0.02638	0.01842	0.01877	0.01985	0.02145
0.02091	0.02288	0.02388	0.01728	0.02625	0.0259	0.02015	0.02391
0.02086	0.02188	0.01934	0.02519	0.01743	0.02029	0.02582	0.01533
0.02086	0.02207	0.02926	0.0139	0.03085	0.03818	0.03555	0.01358
0.02081	0.03679	0.01791	0.02444	0.01782	0.02921	0.03504	0.02491
0.0208	0.02415	0.02363	0.02744	0.02234	0.02815	0.03144	0.02673
0.02079	0.02772	0.0346	0.01937	0.0271	0.03236	0.03864	0.0311
0.02077	0.02895	0.0253	0.0412	0.02995	0.02694	0.02897	0.01841
0.02076	0.02559	0.03256	0.02555	0.03454	0.03423	0.02799	0.0354
0.02075	0.0047	0.02677	0.0081	0.02931	0.02502	0.03333	0.02397
0.02074	0.02024	0.02618	0.02022	0.02347	0.02414	0.02404	0.02984
0.02074	0.00963	0.01277	0.01614	0.01306	0.01666	0.00905	0.00799
0.0207	0.01587	0.0184	0.01606	0.021	0.01973	0.016	0.0098
0.02067	0.01156	0.01779	0.0106	0.01322	0.01607	0.01168	0.01165
0.02066	0.01475	0.01657	0.01434	0.01679	0.01301	0.01786	0.01705
0.02063	0.02394	0.02376	0.03322	0.02809	0.01303	0.01028	0.02092
0.02062	0.01822	0.01571	0.01669	0.01915	0.02452	0.02484	0.02644
0.02056	0.01517	0.01644	0.02344	0.01595	0.01917	0.02249	0.03439
0.02054	0.01698	0.01748	0.02608	0.01957	0.02023	0.02067	0.02341
0.02053	0.01329	0.01617	0.01479	0.01984	0.02026	0.01877	0.01919
0.02052	0.01403	0.02007	0.02821	0.02811	0.02735	0.0323	0.02785
0.02049	0.03095	0.03922	0.03367	0.03519	0.02387	0.02894	0.03582
0.02049	0.02242	0.03014	0.02951	0.01692	0.0206	0.01591	0.02945
0.02049	0.02726	0.03541	0.02985	0.03298	0.02594	0.01236	0.01523
0.02048	0.02746	0.02423	0.0379	0.02439	0.02334	0.02713	0.03767
0.02048	0.01371	0.01761	0.01744	0.01539	0.01655	0.02593	0.01835
0.02047	0.03166	0.02005	0.01111	0.01649	0.0185	0.02418	0.02014
0.02046	0.02296	0.02323	0.01878	0.02018	0.03023	0.04382	0.0382
0.02046	0.02432	0.01678	0.01554	0.01803	0.01776	0.01702	0.02097
0.02042	0.01282	0.02404	0.00973	0.02184	0.02963	0.02285	0.02211
0.02033	0.02034	0.02584	0.02656	0.03081	0.03274	0.02395	0.02552
0.02033	0.01514	0.01283	0.01766	0.01427	0.01672	0.0129	0.01781
0.02028	0.01358	0.01566	0.01961	0.01749	0.01236	0.01671	0.02584
0.02028	0.01882	0.01635	0.02122	0.01664	0.02063	0.01488	0.01541
0.02027	0.02442	0.02872	0.04163	0.02975	0.02719	0.01877	0.02157
0.02026	0.01265	0.02942	0.02488	0.01436	0.01879	0.01325	0.0259
0.02023	0.0165	0.01687	0.02344	0.01621	0.02133	0.01819	0.02376
0.02017	0.01716	0.03796	0.02078	0.02392	0.02599	0.02969	0.02755
0.02016	0.02793	0.02894	0.02177	0.0311	0.03552	0.03438	0.01254
0.02013	0.02552	0.02119	0.01828	0.03583	0.01789	0.03402	0.02954
0.02012	0.02007	0.0182	0.01407	0.02151	0.01946	0.02145	0.02838

0.02012	0.02246	0.01838	0.01676	0.01861	0.02	0.02546	0.02002
0.02012	0.01375	0.01773	0.01266	0.02367	0.01948	0.0237	0.01574
0.02011	0.01986	0.03501	0.02526	0.02978	0.03608	0.02752	0.03615
0.02011	0.02093	0.021	0.02116	0.02365	0.02621	0.01952	0.0181
0.0201	0.02721	0.0332	0.03119	0.02327	0.02752	0.02367	0.02849
0.0201	0.03043	0.03307	0.02327	0.02998	0.03003	0.02036	0.01817
0.02001	0.01728	0.03882	0.01353	0.04185	0.02102	0.01853	0.03263
0.02001	0.02407	0.0236	0.02807	0.01824	0.02729	0.02753	0.03149
0.02	0.03749	0.04121	0.02597	0.04437	0.0478	0.05572	0.04119
0.02	0.02775	0.02339	0.02367	0.0166	0.01855	0.01989	0.02095
0.01997	0.02631	0.03626	0.02454	0.0348	0.03006	0.03768	0.02753
0.01997	0.01952	0.02205	0.02067	0.01707	0.0234	0.0193	0.02625
0.01992	0.01629	0.02004	0.02366	0.01979	0.0198	0.02008	0.01525
0.01985	0.01847	0.02124	0.01658	0.01965	0.01751	0.01922	0.02228
0.01984	0.0213	0.02666	0.01931	0.02522	0.02903	0.03164	0.0267
0.01984	0.01199	0.01516	0.01554	0.01581	0.01747	0.01069	0.01513
0.01984	0.01714	0.01572	0.0082	0.02072	0.01289	0.01765	0.01101
0.01979	0.02475	0.03356	0.02536	0.02787	0.03026	0.02583	0.02038
0.01978	0.01171	0.02731	0.01445	0.01945	0.02426	0.02159	0.01904
0.01977	0.01679	0.0169	0.01788	0.02095	0.02101	0.01174	0.02051
0.01977	0.02304	0.02546	0.0186	0.02422	0.02113	0.02366	0.0194
0.01977	0.01255	0.02594	0.0289	0.01585	0.02032	0.01241	0.01534
0.01974	0.01426	0.00885	0.02467	0.00985	0.01124	0.00901	0.0161
0.01972	0.01272	0.01716	0.01956	0.03846	0.02057	0.0262	0.03711
0.01969	0.02429	0.01797	0.00742	0.01762	0.0215	0.0181	0.0195
0.01968	0.01881	0.01545	0.00661	0.02825	0.01189	0.02318	0.01039
0.01966	0.0338	0.04073	0.02714	0.02901	0.02691	0.0181	0.0361
0.01965	0.01284	0.02521	0.0238	0.02342	0.02959	0.02265	0.02393
0.01963	0.01684	0.02013	0.01075	0.01783	0.01698	0.01494	0.01413
0.01963	0.0123	0.01001	0.02803	0.00918	0.00926	0.00914	0.00993
0.01961	0.00573	0.0115	0.02839	0.03516	0.02117	0.0218	0.02706
0.01959	0.01037	0.00588	0.0063	0.00992	0.01581	0.00814	0.01931
0.01957	0.01987	0.01906	0.02264	0.01969	0.01848	0.01713	0.01917
0.01956	0.01645	0.04581	0.00562	0.01368	0.01507	0.04146	0.03342
0.01945	0.0149	0.02349	0.02247	0.02132	0.02362	0.01948	0.00784
0.01943	0.01616	0.0198	0.01013	0.01128	0.01789	0.00682	0.01829
0.0194	0.01729	0.01961	0.02265	0.0221	0.02389	0.01495	0.01433
0.01937	0.0276	0.03989	0.02275	0.04356	0.04235	0.05546	0.0432
0.01935	0.0216	0.0228	0.01676	0.02357	0.02648	0.01602	0.02332
0.01931	0.03746	0.02587	0.01346	0.01625	0.02634	0.02806	0.00553
0.01929	0.01933	0.04199	0.02538	0.02832	0.03072	0.03627	0.01389
0.01928	0.01392	0.01353	0.0232	0.01295	0.01244	0.01369	0.01353
0.01927	0.01827	0.02331	0.01373	0.02072	0.0171	0.01786	0.01815
0.01924	0.01817	0.0144	0.01499	0.02145	0.01886	0.0172	0.02449
0.01924	0.0079	0.01149	0.01788	0.01462	0.00709	0.01023	0.01412
0.01922	0.01381	0.01543	0.02048	0.01823	0.01773	0.00864	0.01614
0.01921	0.0128	0.02112	0.03224	0.01953	0.0108	0.0046	0.00575
0.01912	0.02232	0.02914	0.01408	0.01716	0.01932	0.02378	0.02126
0.0191	0.02124	0.02569	0.03452	0.01297	0.01919	0.00794	0.02461

0.01909	0.01392	0.01967	0.01716	0.0151	0.02049	0.01593	0.01796
0.01907	0.01248	0.01922	0.01594	0.00732	0.01909	0.01215	0.00467
0.01902	0.01255	0.02447	0.01894	0.00865	0.01724	0.01652	0.01202
0.019	0.01922	0.02208	0.01863	0.02384	0.02602	0.02316	0.02433
0.01896	0.02409	0.03286	0.02448	0.03162	0.02949	0.01475	0.02753
0.01892	0.01955	0.03948	0.02812	0.03034	0.02724	0.03135	0.01941
0.0189	0.02077	0.02411	0.02456	0.01973	0.024	0.02429	0.02814
0.01888	0.0126	0.00772	0.00638	0.02052	0.01931	0.01917	0.02194
0.01886	0.02329	0.03251	0.0231	0.02963	0.03732	0.04409	0.04607
0.01883	0.0078	0.01028	0.01625	0.0083	0.00992	0.01078	0.00828
0.01882	0.00586	0.01372	0.01983	0.01423	0.02113	0.00885	0.01121
0.01881	0.01774	0.01784	0.00858	0.01102	0.00968	0.00811	0.00813
0.0188	0.03148	0.04306	0.01439	0.0428	0.05104	0.05065	0.03183
0.01877	0.01579	0.02665	0.0126	0.02853	0.02961	0.03302	0.03028
0.01874	0.01391	0.01577	0.01628	0.02078	0.00945	0.016	0.01479
0.01872	0.02755	0.03067	0.01722	0.02751	0.02297	0.0262	0.02395
0.01871	0.02317	0.03024	0.02535	0.04386	0.02631	0.03054	0.01879
0.01869	0.02653	0.02666	0.0125	0.02307	0.02184	0.02365	0.02747
0.01867	0.02196	0.02543	0.02208	0.01946	0.01827	0.02074	0.02083
0.01867	0.01945	0.02179	0.02198	0.02033	0.02229	0.01835	0.01664
0.01866	0.02097	0.02082	0.01916	0.02448	0.02024	0.0166	0.02151
0.01866	0.01689	0.02158	0.03408	0.01978	0.0162	0.02389	0.01973
0.01865	0.02433	0.02301	0.01267	0.02534	0.0197	0.03352	0.01973
0.01863	0.01906	0.03607	0.01669	0.03185	0.0221	0.01998	0.0188
0.01862	0.01208	0.01292	0.02275	0.01561	0.01264	0.01357	0.01531
0.01856	0.02738	0.02856	0.01876	0.02541	0.02861	0.0238	0.02896
0.01852	0.03055	0.02626	0.0285	0.01923	0.02271	0.03183	0.0363
0.01852	0.02665	0.01946	0.03549	0.02034	0.01624	0.01182	0.02977
0.01852	0.01826	0.02945	0.01806	0.02792	0.02076	0.01986	0.01646
0.01848	0.01353	0.01413	0.02064	0.01238	0.01349	0.01173	0.01634
0.01846	0.0125	0.01515	0.01923	0.02288	0.01978	0.01073	0.0189
0.01844	0.01759	0.02345	0.01738	0.02499	0.02231	0.01722	0.01951
0.01842	0.01444	0.03416	0.01569	0.02095	0.00639	0.02838	0.0319
0.01842	0.02474	0.03462	0.01362	0.0248	0.02414	0.02306	0.0293
0.01842	0.02159	0.02223	0.01269	0.02015	0.03132	0.02295	0.01825
0.01842	0.01004	0.02034	0.01309	0.01657	0.02113	0.01846	0.01774
0.0184	0.01795	0.03599	0.01032	0.03443	0.03535	0.03302	0.03111
0.01838	0.03358	0.01669	0.03837	0.0132	0.01445	0.01891	0.02702
0.01837	0.02169	0.02156	0.0273	0.02007	0.02564	0.01706	0.02054
0.01837	0.01413	0.01652	0.0201	0.0143	0.01597	0.00971	0.01852
0.01837	0.01441	0.03214	0.01901	0.02395	0.02146	0.00891	0.01669
0.01837	0.01754	0.00324	0.00997	0.00099	0.00888	0.01106	0.01472
0.01834	0.02523	0.02442	0.01197	0.02278	0.01863	0.01205	0.00385
0.01826	0.01232	0.03446	0.02902	0.02026	0.01871	0.03519	0.02192
0.01826	0.01697	0.01415	0.01676	0.01065	0.0152	0.01302	0.01671
0.01825	0.0218	0.02964	0.01151	0.02302	0.03247	0.03035	0.0383
0.01825	0.00996	0.01272	0.02125	0.01634	0.0146	0.01194	0.01223
0.01823	0.02668	0.02113	0.0309	0.01634	0.02051	0.0227	0.03255
0.01823	0.01964	0.0219	0.0189	0.02567	0.02468	0.01206	0.02292

0.01823	0.0185	0.02234	0.02029	0.01844	0.02231	0.02207	0.01506
0.01815	0.0135	0.01919	0.03984	0.01554	0.01305	0.01511	0.01063
0.01811	0.01788	0.01574	0.01145	0.01988	0.02574	0.01995	0.02283
0.01809	0.02224	0.02959	0.02111	0.02989	0.03476	0.03475	0.02814
0.01808	0.02149	0.02978	0.03246	0.02653	0.02541	0.01433	0.03185
0.01807	0.02612	0.02601	0.03234	0.01581	0.01587	0.0103	0.03082
0.01805	0.01162	0.01492	0.01264	0.01837	0.01165	0.00326	0.01599
0.01801	0.01057	0.01093	0.01007	0.01285	0.01273	0.01191	0.00418
0.01793	0.01771	0.02985	0.02409	0.02968	0.02948	0.02214	0.01819
0.01791	0.0197	0.0248	0.02366	0.02345	0.022	0.02036	0.02454
0.01788	0.01844	0.01985	0.01814	0.01996	0.03076	0.01678	0.02566
0.01787	0.02328	0.0337	0.02134	0.03597	0.03568	0.04696	0.04208
0.01786	0.01236	0.00828	0.0195	0.0133	0.018	0.00798	0.01527
0.01785	0.01532	0.01937	0.02178	0.01445	0.01543	0.0163	0.02157
0.01785	0.0074	0.01188	0.00646	0.00767	0.01162	0.00777	0.00887
0.01783	0.01809	0.01607	0.01668	0.01533	0.01701	0.01526	0.02045
0.01782	0.01372	0.01066	0.01113	0.01526	0.03808	0.02624	0.04602
0.01779	0.01569	0.02272	0.01596	0.01983	0.02262	0.01912	0.01834
0.01775	0.0162	0.02275	0.02964	0.02199	0.02212	0.0119	0.01805
0.01774	0.02596	0.00653	0.01625	0.0036	0.01673	0.03726	0.02645
0.01767	0.01807	0.02061	0.01864	0.01929	0.01976	0.01682	0.01944
0.01764	0.0122	0.01316	0.00999	0.01077	0.01192	0.01001	0.01164
0.01762	0.01396	0.02122	0.00522	0.01998	0.01751	0.02292	0.02083
0.01754	0.01782	0.01891	0.01461	0.02853	0.03171	0.02171	0.02764
0.01753	0.01384	0.01697	0.01222	0.01673	0.01861	0.01805	0.02093
0.01752	0.04724	0.03842	0.02718	0.04409	0.04908	0.07177	0.04078
0.01751	0.02175	0.02681	0.01316	0.03528	0.02886	0.04159	0.02482
0.01751	0.01397	0.01236	0.01729	0.00948	0.01003	0.02085	0.01386
0.01749	0.01761	0.0261	0.03668	0.02291	0.02341	0.02395	0.02277
0.01747	0.0163	0.02234	0.00863	0.01619	0.01252	0.01639	0.01703
0.01747	0.02888	0.02176	0.02136	0.03303	0.02529	0.02454	0.01206
0.01741	0.0345	0.02332	0.03132	0.01927	0.03819	0.05331	0.02957
0.0174	0.01435	0.00972	0.01321	0.01004	0.00982	0.00783	0.01076
0.01737	0.02417	0.0255	0.01402	0.0248	0.02532	0.03315	0.03645
0.01737	0.01672	0.01221	0.01149	0.01797	0.0175	0.02054	0.0175
0.0173	0.01403	0.01252	0.00489	0.01354	0.00866	0.00526	0.00497
0.01728	0.01967	0.01914	0.01925	0.02789	0.02757	0.01078	0.02071
0.01725	0.01597	0.0287	0.01785	0.02681	0.0292	0.02828	0.02802
0.01724	0.0133	0.01346	0.02866	0.01755	0.01425	0.01098	0.00982
0.01723	0.02133	0.02533	0.0261	0.028	0.02586	0.01774	0.02706
0.01722	0.00845	0.01562	0.00986	0.01758	0.01744	0.0203	0.01774
0.01721	0.01685	0.01983	0.01186	0.02033	0.01101	0.02169	0.01638
0.01715	0.02303	0.03712	0.00975	0.0328	0.02645	0.03265	0.026
0.01714	0.03327	0.03888	0.01516	0.02748	0.02144	0.02573	0.01729
0.01714	0.00697	0.01121	0.03351	0.01608	0.02255	0.01733	0.014
0.01713	0.00777	0.01378	0.00956	0.02192	0.02328	0.02501	0.02053
0.01709	0.01623	0.01834	0.02249	0.0405	0.02309	0.03111	0.03788
0.01708	0.0115	0.01645	0.01609	0.0129	0.02413	0.02147	0.02437
0.01707	0.01489	0.01851	0.01385	0.01105	0.02002	0.02278	0.01898

0.01706	0.02166	0.01655	0.01459	0.02522	0.01783	0.02287	0.02511
0.01705	0.00452	0.00839	0.01362	0.00644	0.00252	0.00319	0.00431
0.01703	0.02448	0.04038	0.03785	0.03074	0.02645	0.03783	0.02475
0.01703	0.0182	0.01699	0.01417	0.01876	0.01799	0.01236	0.01595
0.01703	0.01702	0.01539	0.01182	0.02684	0.01992	0.03085	0.00672
0.01702	0.01387	0.01932	0.02147	0.00956	0.01077	0.01788	0.01625
0.01702	0.01547	0.01499	0.01765	0.01607	0.01371	0.01434	0.01251
0.017	0.01763	0.01236	0.02041	0.01757	0.02168	0.02557	0.0178
0.01696	0.02438	0.03836	0.02048	0.03388	0.03815	0.03422	0.04003
0.01688	0.01016	0.03146	0.01158	0.02863	0.02091	0.01886	0.02619
0.01687	0.01841	0.01331	0.01471	0.01177	0.01559	0.02082	0.02811
0.01687	0.01034	0.01418	0.00242	0.01655	0.01761	0.01641	0.01827
0.01686	0.01589	0.00764	0.01709	0.00946	0.00969	0.01582	0.00685
0.01685	0.01492	0.02548	0.02661	0.03081	0.02449	0.01867	0.01783
0.01682	0.02142	0.02767	0.02352	0.02053	0.0226	0.02323	0.01832
0.01681	0.02563	0.02839	0.01787	0.02107	0.02736	0.02098	0.02665
0.01678	0.01978	0.01902	0.0156	0.01479	0.01579	0.01719	0.01731
0.01677	0.02257	0.02279	0.01765	0.01692	0.01954	0.01352	0.01358
0.01675	0.02004	0.01521	0.02638	0.02367	0.03034	0.01963	0.01181
0.01671	0.01978	0.01515	0.01741	0.01058	0.01524	0.01929	0.0242
0.01669	0.02487	0.02309	0.01126	0.01949	0.02134	0.03022	0.02397
0.01668	0.01609	0.02505	0.01663	0.02342	0.02515	0.01465	0.02087
0.01666	0.01738	0.01167	0.01363	0.01624	0.01716	0.0113	0.00969
0.01663	0.0285	0.03606	0.0251	0.02923	0.03031	0.02353	0.02521
0.01662	0.0187	0.02183	0.0241	0.02414	0.02846	0.01495	0.01684
0.01655	0.01951	0.02699	0.03414	0.01321	0.0272	0.02359	0.02671
0.01655	0.00991	0.01267	0.01549	0.01671	0.01768	0.00419	0.01559
0.01653	0.01722	0.0198	0.02425	0.02028	0.01773	0.01239	0.0117
0.01651	0.02482	0.02765	0.01907	0.02882	0.01361	0.02846	0.02083
0.01651	0.01501	0.01091	0.01865	0.0175	0.0183	0.01546	0.01596
0.0165	0.00793	0.0117	0.01085	0.01301	0.01649	0.01214	0.01324
0.01649	0.01223	0.01482	0.02171	0.01631	0.01713	0.01346	0.01932
0.01648	0.01639	0.02513	0.02584	0.02146	0.02167	0.02256	0.02281
0.01646	0.01429	0.00731	0.01602	0.01146	0.01267	0.00949	0.01774
0.01645	0.01541	0.01983	0.01868	0.02215	0.01918	0.01791	0.021
0.01643	0.03469	0.01841	0.01814	0.01832	0.02281	0.01269	0.0231
0.0164	0.01903	0.02244	0.018	0.03258	0.01827	0.02534	0.01786
0.01639	0.0068	0.00241	0.01073	0.00315	0.00327	0.00196	0.0036
0.01632	0.02135	0.01007	0.01272	0.00996	0.01052	0.00401	0.01381
0.01631	0.01963	0.01563	0.01892	0.01776	0.03608	0.0249	0.02883
0.01627	0.02254	0.01897	0.00729	0.01619	0.02073	0.01725	0.01419
0.01625	0.02244	0.02862	0.01801	0.02531	0.01766	0.01462	0.01982
0.01624	0.00949	0.00801	0.00305	0.02008	0.01916	0.0094	0.00572
0.01623	0.01393	0.02948	0.03328	0.02294	0.02549	0.02469	0.0187
0.01623	0.01823	0.0204	0.01885	0.0162	0.01859	0.022	0.0181
0.01622	0.01889	0.02398	0.02628	0.02604	0.02325	0.01546	0.01741
0.01622	0.01387	0.01372	0.01697	0.0129	0.01589	0.01525	0.01291
0.01617	0.01498	0.03521	0.01705	0.02417	0.02095	0.02194	0.0285
0.01617	0.01378	0.01652	0.02165	0.01619	0.02071	0.02081	0.02052

0.01617	0.0171	0.0211	0.01311	0.01417	0.01784	0.01405	0.01524
0.01616	0.02002	0.01468	0.04721	0.02009	0.02972	0.03033	0.03301
0.01616	0.01457	0.01426	0.00924	0.04363	0.04247	0.00944	0.03118
0.01616	0.02575	0.02641	0.01876	0.0188	0.01554	0.01109	0.01987
0.01612	0.0081	0.01036	0.00556	0.01152	0.0114	0.0115	0.01043
0.0161	0.01509	0.01401	0.0168	0.02089	0.01813	0.02221	0.03024
0.01609	0.0144	0.01513	0.01102	0.02867	0.01081	0.01126	0.01842
0.01604	0.01858	0.01888	0.01836	0.02814	0.02551	0.01553	0.01414
0.01603	0.00947	0.01198	0.01676	0.01115	0.01671	0.01159	0.00913
0.01602	0.02484	0.02015	0.00716	0.04144	0.02681	0.0315	0.03504
0.01597	0.02345	0.02803	0.01757	0.03508	0.01826	0.02921	0.01406
0.01596	0.00983	0.00997	0.00592	0.01649	0.01582	0.01724	0.0107
0.01596	0.01196	0.01629	0.02179	0.01438	0.01129	0.01333	0.00871
0.01596	0.01059	0.01726	0.00734	0.02073	0.01791	0.01159	0.00757
0.01595	0.01216	0.01349	0.01156	0.01408	0.01006	0.01607	0.00425
0.01593	0.02037	0.0232	0.01206	0.01677	0.01905	0.02423	0.02021
0.01593	0.0121	0.01332	0.01806	0.01455	0.01796	0.01549	0.01994
0.01591	0.02168	0.0293	0.01844	0.02479	0.02293	0.02075	0.02043
0.01591	0.01722	0.01277	0.0066	0.02001	0.02062	0.00933	0.01838
0.0159	0.01367	0.03676	0.04708	0.03481	0.02015	0.02033	0.03725
0.01589	0.02101	0.01844	0.01207	0.02441	0.01991	0.02492	0.01842
0.01587	0.07472	0.00048	0.0494	0.0181	0.04543	0.00494	0.02304
0.01586	0.01786	0.01729	0.02311	0.02781	0.02172	0.01231	0.0196
0.01584	0.01453	0.01741	0.01703	0.01874	0.0265	0.02955	0.02475
0.01583	0.0187	0.01304	0.01846	0.00899	0.01117	0.01326	0.01508
0.01582	0.01342	0.01102	0.02013	0.00928	0.01062	0.02214	0.01831
0.01581	0.01187	0.01443	0.02057	0.01766	0.0157	0.01328	0.03083
0.01581	0.03055	0.03201	0.01244	0.02615	0.02397	0.02994	0.0283
0.01579	0.01563	0.02327	0.01678	0.02472	0.03142	0.02388	0.02655
0.01573	0.01869	0.02612	0.01879	0.03019	0.03133	0.03223	0.01875
0.01573	0.01046	0.01167	0.01975	0.00836	0.0129	0.00713	0.00839
0.01571	0.01609	0.02204	0.0272	0.01793	0.02362	0.02454	0.0204
0.01569	0.02028	0.02141	0.02588	0.02534	0.03277	0.02979	0.0304
0.01569	0.01552	0.02073	0.00974	0.02699	0.01984	0.02894	0.01195
0.01568	0.011	0.0082	0.01847	0.0083	0.01237	0.01148	0.01426
0.01567	0.01703	0.02018	0.01439	0.01268	0.01875	0.01667	0.01618
0.01564	0.03408	0.0146	0.01977	0.01599	0.02814	0.02399	0.02046
0.01563	0.02708	0.01506	0.01815	0.02125	0.02203	0.02854	0.03125
0.01559	0.01638	0.02185	0.04397	0.01899	0.03643	0.00848	0.02073
0.01559	0.01399	0.01714	0.02001	0.01435	0.01612	0.01366	0.01578
0.01557	0.01167	0.04969	0.02871	0.01504	0.00899	0.029	0.02524
0.01552	0.01499	0.01528	0.03234	0.0192	0.02321	0.0165	0.02668
0.01551	0.01882	0.02912	0.02059	0.01397	0.01692	0.01255	0.01452
0.01548	0.0113	0.0208	0.01371	0.01904	0.02075	0.01982	0.01953
0.01546	0.01441	0.02423	0.00891	0.01651	0.02726	0.02938	0.01965
0.01545	0.01254	0.00699	0.01758	0.01361	0.01937	0.02012	0.00812
0.01542	0.00993	0.01358	0.00631	0.01063	0.01047	0.00981	0.01333
0.0154	0.00866	0.00801	0.0051	0.0089	0.01435	0.01087	0.01007
0.0154	0.01813	0.01391	0.01147	0.01808	0.01849	0.01073	0.00951

0.01538	0.01658	0.0183	0.01167	0.0201	0.01637	0.0179	0.02082
0.01538	0.01236	0.01416	0.01876	0.0115	0.01924	0.01926	0.016
0.01536	0.01366	0.02227	0.01645	0.02366	0.02159	0.01224	0.02212
0.01533	0.02217	0.02256	0.02638	0.0212	0.0189	0.0229	0.01776
0.01533	0.01026	0.0102	0.01335	0.00975	0.01174	0.00956	0.00873
0.01532	0.00779	0.00532	0.00816	0.02011	0.00752	0.01971	0.02251
0.01529	0.01979	0.01024	0.022	0.02484	0.01632	0.02037	0.0189
0.01529	0.01692	0.01378	0.01965	0.01592	0.0076	0.01899	0.01332
0.01523	0.01684	0.01333	0.00578	0.01512	0.01459	0.01235	0.0212
0.01522	0.01479	0.02408	0.01263	0.02852	0.02894	0.01769	0.02122
0.01522	0.0081	0.01155	0.01722	0.01458	0.00971	0.0109	0.00566
0.01516	0.02029	0.01861	0.01751	0.02066	0.01994	0.01674	0.01546
0.01515	0.01233	0.01805	0.02126	0.01665	0.0171	0.01682	0.02139
0.01515	0.01629	0.0263	0.01435	0.0249	0.02224	0.0215	0.01398
0.01514	0.01138	0.02086	0.02067	0.0142	0.01666	0.01658	0.01357
0.01507	0.01305	0.01734	0.02401	0.01637	0.01428	0.00938	0.01033
0.01506	0.01132	0.01101	0.01398	0.01129	0.00966	0.0097	0.01814
0.01502	0.0118	0.00635	0.01381	0.00882	0.00978	0.01487	0.01109
0.01501	0.01578	0.01344	0.01387	0.01709	0.01643	0.02595	0.01854
0.01499	0.01294	0.01594	0.01341	0.01866	0.02338	0.01711	0.00739
0.01498	0.01052	0.01451	0.00598	0.026	0.01278	0.01924	0.01355
0.01498	0.01174	0.01547	0.00652	0.03235	0.02705	0.01345	0.01286
0.01495	0.02168	0.04349	0.01674	0.0186	0.02106	0.02324	0.02932
0.01494	0.01257	0.0148	0.0091	0.01424	0.01259	0.01452	0.01075
0.01494	0.01427	0.02101	0.02116	0.01968	0.0219	0.00903	0.0097
0.01493	0.01628	0.0173	0.06646	0.00951	0.01318	0.02381	0.02257
0.01491	0.0107	0.00688	0.00701	0.0047	0.00645	0.00743	0.00856
0.0149	0.01469	0.01878	0.01553	0.02253	0.02319	0.03004	0.01048
0.01488	0.01387	0.02227	0.01621	0.01853	0.0283	0.02862	0.02032
0.01487	0.02093	0.02629	0.01816	0.01906	0.02343	0.0264	0.02961
0.01486	0.01603	0.01378	0.02044	0.01744	0.00228	0.01293	0.01193
0.01486	0.02514	0.02028	0.01479	0.01833	0.01569	0.01919	0.00315
0.01483	0.02154	0.01541	0.01346	0.01802	0.02451	0.02087	0.01449
0.01482	0.01362	0.01627	0.00985	0.02142	0.01528	0.01339	0.02223
0.01479	0.01837	0.02733	0.01694	0.02307	0.02305	0.02246	0.01679
0.01478	0.01201	0.0139	0.01529	0.01096	0.01437	0.01407	0.01296
0.01476	0.0174	0.01028	0.01709	0.01187	0.02632	0.01154	0.00676
0.01471	0.01551	0.02654	0.01871	0.02426	0.0226	0.02754	0.02406
0.01469	0.01676	0.02356	0.0038	0.03477	0.02835	0.03167	0.02145
0.01466	0.01043	0.01305	0.01052	0.00213	0.01417	0.01346	0.01545
0.01462	0.01084	0.01333	0.00679	0.01423	0.01759	0.01545	0.01483
0.01461	0.00424	0.00785	0.00933	0.00582	0.00987	0.01436	0.02185
0.01461	0.00691	0.0104	0.01006	2E-05	0.00936	0.00993	0.01399
0.01461	0.01498	0.01788	0.00838	0.01703	0.00885	0.01204	0.01005
0.0146	0.01452	0.02049	0.02606	0.01582	0.01699	0.00939	0.01898
0.01459	0.01679	0.01413	0.00854	0.01945	0.01516	0.0193	0.01834
0.01454	0.0138	0.01499	0.00776	0.01173	0.01589	0.01196	0.00554
0.01451	0.01799	0.01904	0.02024	0.02169	0.0178	0.01468	0.00457
0.0145	0.02504	0.0242	0.0215	0.01945	0.01681	0.00919	0.01505

0.0145	0.01454	0.02145	0.01296	0.0185	0.01953	0.02071	0.01442
0.01445	0.01806	0.0241	0.01848	0.0355	0.03237	0.02808	0.02245
0.01444	0.00807	0.00939	0.0192	0.01182	0.01481	0.00941	0.01017
0.01443	0.01541	0.015	0.01364	0.01419	0.01722	0.02182	0.01778
0.01442	0.02807	0.02294	0.02622	0.01882	0.01796	0.0279	0.0238
0.01439	0.0159	0.0191	0.02272	0.01978	0.01469	0.01634	0.0149
0.01436	0.00944	0.04099	0.0205	0.03065	0.02013	0.01977	0.02477
0.01436	0.02574	0.02067	0.0236	0.02213	0.02431	0.01782	0.02342
0.0143	0.01286	0.01302	0.01188	0.01403	0.0125	0.01082	0.01379
0.0142	0.01717	0.01832	0.01126	0.01712	0.01566	0.01396	0.01371
0.01416	0.02383	0.02566	0.00977	0.03159	0.04023	0.02911	0.02139
0.01416	0.0104	0.01583	0.00924	0.01128	0.01184	0.00548	0.01417
0.01412	0.01244	0.02246	0.01981	0.02039	0.01405	0.01427	0.01274
0.01411	0.01634	0.01532	0.01591	0.01378	0.0126	0.01669	0.01405
0.0141	0.02027	0.04229	0.00995	0.0481	0.04018	0.03986	0.0204
0.0141	0.01732	0.02622	0.01036	0.02724	0.0332	0.03259	0.01223
0.01407	0.01332	0.01291	0.01713	0.01296	0.01455	0.00855	0.01175
0.01404	0.01242	0.01416	0.00993	0.00942	0.01052	0.01649	0.00973
0.01403	0.0129	0.02317	0.02541	0.0208	0.02139	0.02033	0.02761
0.01403	0.01453	0.01347	0.01144	0.02189	0.02019	0.0192	0.01887
0.01402	0.01058	0.01341	0.01281	0.01437	0.01413	0.01333	0.01636
0.014	0.01278	0.01595	0.00909	0.00957	0.0129	0.00795	0.01503
0.01399	0.02115	0.02461	0.00304	0.02526	0.02366	0.02429	0.02243
0.01399	0.00782	0.01666	0.00848	0.01763	0.01849	0.01708	0.01248
0.01398	0.01398	0.0141	0.01433	0.01506	0.01403	0.03136	0.02266
0.01398	0.00853	0.02152	0.00909	0.01599	0.01298	0.00786	0.00982
0.01397	0.01281	0.01491	0.01194	0.01391	0.0189	0.00981	0.01462
0.01397	0.01007	0.01098	0.0253	0.01128	0.01483	0.01015	0.01192
0.01394	0.01101	0.01492	0.0055	0.02839	0.02715	0.01477	0.01696
0.01394	0.01157	0.00945	0.01312	0.01597	0.0101	0.01045	0.00913
0.01393	0.01324	0.02462	0.00957	0.00267	0.02722	0.02249	0.02339
0.01393	0.01355	0.01933	0.00943	0.03036	0.02209	0.01788	0.02221
0.01392	0.01348	0.01512	0.0102	0.01892	0.02066	0.01584	0.01844
0.01391	0.01211	0.00656	0.00451	0.00555	0.01075	0.00491	0.00065
0.01389	0.00931	0.01277	0.00507	0.01435	0.01621	0.01696	0.0181
0.01382	0.01516	0.02856	0.01724	0.01995	0.02286	0.01952	0.02503
0.01381	0.01391	0.00987	0.009	0.01399	0.01497	0.01515	0.02047
0.0138	0.00965	0.01918	0.01134	0.01604	0.02019	0.01624	0.01644
0.01379	0.00769	0.01007	0.00098	0.01097	0.01218	0.0037	0.00263
0.01377	0.01776	0.02299	0.01657	0.01593	0.01808	0.01748	0.01336
0.01376	0.02476	0.02539	0.01012	0.0232	0.02314	0.0451	0.02434
0.01363	0.02816	0.02585	0.02097	0.01736	0.01854	0.02433	0.02593
0.01363	0.01165	0.01339	0.01908	0.01587	0.01578	0.01918	0.02293
0.01363	0.00967	0.01348	0.00976	0.01963	0.0184	0.01316	0.01829
0.01363	0.01424	0.01661	0.01772	0.01691	0.01505	0.01116	0.01406
0.01362	0.02873	0.02199	0.00694	0.03254	0.03937	0.03496	0.02062
0.01361	0.0112	0.01848	0.02284	0.01245	0.01883	0.01444	0.02134
0.0136	0.02171	0.01565	0.00991	0.02042	0.01873	0.02078	0.02239
0.01356	0.00537	0.02387	0.02439	0.02189	0.01077	0.01461	0.01525

0.01355	0.01481	0.01265	0.01669	0.01883	0.01499	0.01717	0.01675
0.01354	0.00939	0.01677	0.01477	0.01266	0.01373	0.01209	0.01343
0.01353	0.00824	0.01968	0.00849	0.01874	0.02335	0.02016	0.02189
0.01353	0.0119	0.01293	0.01072	0.01382	0.01297	0.01316	0.01216
0.01349	0.00931	0.02447	0.02207	0.01527	0.02679	0.02401	0.02421
0.01348	0.00753	0.05151	0.02519	0.03007	0.00392	0.06502	0.03508
0.01346	0.00984	0.01611	0.0035	0.02985	0.02094	0.02912	0.03265
0.01346	0.02646	0.02026	0.00955	0.03193	0.02044	0.02222	0.03228
0.01346	0.01836	0.03269	0.02124	0.01339	0.02233	0.02633	0.02494
0.01345	0.01891	0.02229	0.01041	0.02054	0.01488	0.0178	0.01356
0.01345	0.01357	0.01677	0.00784	0.01084	0.00888	0.01593	0.01275
0.01343	0.01117	0.00878	0.00737	0.00731	0.00608	0.00337	0.00839
0.01339	0.0142	0.01667	0.0071	0.02366	0.02054	0.01176	0.0219
0.01338	0.00685	0.01164	0.00964	0.01252	0.01352	0.01592	0.02043
0.01338	0.01196	0.02844	0.01653	0.01792	0.00485	0.00989	0.01955
0.01338	0.0049	0.01834	0.01122	0.02099	0.01358	0.02327	0.01643
0.01338	0.02137	0.02454	0.00695	0.02466	0.08343	0.09905	0.015
0.01338	0.00999	0.01325	0.0144	0.0122	0.01231	0.0067	0.01016
0.01337	0.0064	0.01853	0.00361	0.01448	0.01452	0.01828	0.02405
0.01337	0.02616	0.00759	0.00767	0.00861	0.01377	0.01003	0.00924
0.01332	0.01922	0.02476	0.0099	0.02277	0.0275	0.03194	0.0115
0.01328	0.00869	0.01014	0.01171	0.0109	0.00964	0.01099	0.01136
0.01326	0.01486	0.01232	0.00891	0.01056	0.01546	0.00887	0.00544
0.01325	0.01544	0.02369	0.01747	0.02167	0.02322	0.01092	0.01785
0.01325	0.02082	0.01026	0.01341	0.01134	0.00775	0.00238	0.01627
0.01324	0.01464	0.02533	0.01224	0.02326	0.02104	0.02837	0.02058
0.01323	0.00543	0.00843	0.01009	0.00736	0.01575	0.00893	0.01323
0.01323	0.01203	0.01363	0.01054	0.01371	0.01245	0.01102	0.01201
0.01322	0.01915	0.01741	0.01687	0.01761	0.02357	0.02956	0.02633
0.01319	0.03599	0.02682	0.0299	0.03104	0.03019	0.01375	0.02493
0.01319	0.0118	0.01699	0.0112	0.00938	0.01392	0.02127	0.01565
0.01318	0.00527	0.0233	0.0135	0.02523	0.02258	0.02919	0.03972
0.01316	0.0117	0.01837	0.01439	0.01504	0.01564	0.0168	0.01609
0.01311	0.01659	0.01571	0.01299	0.01643	0.01694	0.04829	0.02435
0.01308	0.01424	0.00913	0.02487	0.01974	0.01165	0.01116	0.01527
0.01307	0.01208	0.01227	0.01799	0.01275	0.01417	0.01904	0.01583
0.01305	0.00904	0.01447	0.01098	0.01478	0.01621	0.0141	0.01053
0.01303	0.01836	0.01351	0.01111	0.01494	0.01358	0.01514	0.0107
0.01301	0.03335	0.0403	0.01702	0.02948	0.03016	0.02807	0.01899
0.01301	0.01357	0.01414	0.00372	0.04297	0.03472	0.03523	0.01293
0.01301	0.03302	0.01514	0.04981	0.01927	0.03404	0.01515	0.01099
0.01301	0.00889	0.01251	0.01834	0.00541	0.01138	0.0053	0.00497
0.01299	0.02902	0.02545	0.01441	0.03011	0.03905	0.02011	0.02901
0.01299	0.01368	0.01456	0.0086	0.02188	0.01783	0.01351	0.01316
0.01297	0.02966	0.01763	0.00254	0.03356	0.01954	0.02457	0.02509
0.01296	0.02056	0.03182	0.00847	0.02788	0.01986	0.02902	0.01929
0.01296	0.00325	0.00709	0.02262	0.00443	0.01809	0.00709	0.00635
0.01292	0.00836	0.01342	0.00891	0.01235	0.01105	0.00679	0.0067
0.01286	0.00968	0.00356	0.00539	0.00152	0.00802	0.00333	0.01108

0.01286	0.01254	0.02214	0.01716	0.01324	0.0191	0.01219	0.0071
0.01285	0.01475	0.01887	0.00429	0.03589	0.02369	0.02949	0.01709
0.01281	0.01483	0.01843	0.01788	0.02562	0.02372	0.0368	0.02871
0.0128	0.00946	0.02524	0.0125	0.01709	0.02238	0.02249	0.01701
0.01277	0.01365	0.01251	0.01395	0.01363	0.01359	0.01545	0.02065
0.01277	0.01097	0.01122	0.02118	0.01206	0.01374	0.00679	0.01372
0.01276	0.01396	0.0205	0.0094	0.01876	0.02395	0.02213	0.02076
0.01274	0.01101	0.0157	0.01618	0.00998	0.01579	0.0076	0.00935
0.01273	0.0122	0.01125	0.01428	0.01061	0.01103	0.01529	0.00835
0.01272	0.03148	0.01774	0.02114	0.02351	0.01689	0.0159	0.02138
0.01271	0.01012	0.01904	0.0051	0.02518	0.01301	0.00887	0.0173
0.0127	0.01373	0.01715	0.00771	0.01187	0.01192	0.01486	0.00882
0.01269	0.01439	0.0259	0.01817	0.01951	0.00454	0.02437	0.03089
0.01265	0.01457	0.00555	0.00444	0.02591	0.01189	0.00495	0.02227
0.01265	0.0116	0.01494	0.00708	0.0157	0.01477	0.00838	0.01414
0.01265	0.01756	0.01024	0.01452	0.00625	0.01281	0.02609	0.01227
0.01264	0.02532	0.02831	0.0137	0.03015	0.03854	0.03824	0.03721
0.01263	0.01245	0.01567	0.02514	0.02439	0.01783	0.01141	0.01321
0.01263	0.01118	0.01744	0.01669	0.01049	0.01138	0.0085	0.01132
0.01263	0.00944	0.01118	0.01477	0.01027	0.0118	0.00299	0.00982
0.01262	0.01407	0.01288	0.02262	0.01446	0.01188	0.00732	0.0195
0.01262	0.01204	0.00928	0.01876	0.01023	0.0117	0.00979	0.01438
0.01262	0.0073	0.0103	0.00551	0.00976	0.00974	0.00823	8E-05
0.01258	0.0101	0.01591	0.01148	0.00965	0.01032	0.01001	0.00667
0.01256	0.01419	0.02579	0.01362	0.02951	0.02379	0.01409	0.01433
0.01256	0.01203	0.01186	0.0199	0.00673	0.00828	0.00914	0.01108
0.01255	0.01162	0.01199	0.01095	0.01747	0.0156	0.01205	0.01155
0.01254	0.02402	0.03021	0.01273	0.00938	0.01	0.01539	0.02637
0.01252	0.01507	0.02838	0.01808	0.02756	0.02743	0.0176	0.02199
0.01251	0.01212	0.00882	0.0168	0.00972	0.00942	0.01591	0.00912
0.01247	0.01998	0.01828	0.01664	0.02005	0.01906	0.01455	0.01882
0.01244	0.01636	0.02633	0.01329	0.00931	0.01613	0.01239	0.01822
0.01243	0.01813	0.02019	0.00427	0.01704	0.02286	0.00401	0.01739
0.01242	0.00568	0.00739	0.01109	0.00704	0.01082	0.00851	0.01641
0.0124	0.01681	0.00846	0.00702	0.00904	0.01061	0.00068	0.00968
0.01236	0.00795	0.01467	0.01085	0.01217	0.01571	0.00832	0.01471
0.01236	0.03087	0.00229	0.03533	0.051	0.00926	0.00492	0.0029
0.01234	0.00761	0.01363	0.00893	0.01048	0.00966	0.01903	0.01592
0.0123	0.0193	0.01418	0.00696	0.01623	0.0247	0.01788	0.02248
0.01229	0.02143	0.01721	0.01235	0.01624	0.01512	0.01014	0.00928
0.01226	0.01097	0.00739	0.00687	0.00881	0.00636	0.0126	0.01093
0.01225	0.02182	0.01919	0.01791	0.01704	0.02006	0.02172	0.01788
0.01224	0.01559	0.01454	0.01581	0.01452	0.01477	0.01454	0.01332
0.01222	0.01115	0.01415	0.01619	0.01425	0.01331	0.00783	0.01214
0.01221	0.01411	0.01153	0.02415	0.01073	0.02075	0.00946	0.01825
0.01208	0.00723	0.00691	0.0119	0.00846	0.00868	0.01032	0.01347
0.01208	0.01129	0.00885	0.01369	0.01086	0.00764	0.00753	0.00849
0.01207	0.01053	0.01667	0.01659	0.01161	0.01091	0.01682	0.01586
0.01206	0.02848	0.02493	0.0183	0.01884	0.024	0.01726	0.01814

0.01206	0.00601	0.00396	0.0095	0.00707	0.01022	0.0031	0.00639
0.01202	0.01801	0.01091	0.01473	0.01804	0.01591	0.01939	0.02393
0.012	0.0083	0.01023	0.01234	0.01079	0.01019	0.00861	0.01492
0.01194	0.01048	0.01849	0.01657	0.01224	0.01293	0.01298	0.01719
0.0119	0.01882	0.02048	0.00956	0.02193	0.02175	0.01955	0.02993
0.01188	0.01411	0.01296	0.01438	0.01377	0.01548	0.01573	0.0214
0.01188	0.00691	0.01419	0.00829	0.00329	0.0154	0.01119	0.00637
0.01186	0.00793	0.0172	0.0056	0.01438	0.01079	0.01598	0.01693
0.01186	0.00771	0.00627	0.01127	0.00968	0.01083	0.01253	0.00809
0.01184	0.01754	0.02039	0.0063	0.02346	0.01298	0.02395	0.00886
0.01184	0.00487	0.01386	0.01166	0.01033	0.00339	0.00512	0.00842
0.01182	0.0098	0.0167	0.00871	0.01638	0.01866	0.01667	0.01839
0.01181	0.01349	0.02561	0.00782	0.02758	0.01954	0.0211	0.01698
0.01181	0.00866	0.00862	0.00974	0.01045	0.0165	0.0042	0.0094
0.0118	0.01619	0.00824	0.00245	0.00478	0.01312	0.0072	0.01574
0.01179	0.00449	0.00436	0.0031	0.00198	0.00752	0.00853	0.00073
0.01176	0.02161	0.01662	0.03397	0.06562	0.02671	0.0313	0.01627
0.01175	0.01253	0.0152	0.01813	0.01833	0.01831	0.01505	0.01686
0.01174	0.00405	0.00516	0.00704	0.00366	0.0052	0.01242	0.01283
0.01172	0.0115	0.01704	0.01767	0.01404	0.01458	0.01198	0.01241
0.01171	0.01194	0.0105	0.02332	0.012	0.02106	0.02157	0.00734
0.0117	0.01205	0.01253	0.01624	0.01397	0.01397	0.01262	0.01342
0.01169	0.02295	0.02327	0.0109	0.02255	0.02195	0.02921	0.02362
0.01167	0.01181	0.01945	0.01951	0.01727	0.01499	0.02274	0.0367
0.01167	0.01001	0.01157	0.01183	0.01089	0.00674	0.00387	0.0097
0.01163	0.01762	0.01579	0.01502	0.01711	0.01282	0.01244	0.01707
0.01162	0.02497	0.01739	0.0052	0.08848	0.03688	0.0205	0.02373
0.01161	0.00924	0.01057	0.01122	0.01312	0.01184	0.02085	0.0145
0.01157	0.0185	0.01609	0.02233	0.0239	0.02903	0.03571	0.04389
0.01157	0.03194	0.03899	0.01841	0.0329	0.02495	0.04583	0.03454
0.01157	0.02139	0.01672	0.00547	0.02861	0.01315	0.02397	0.01825
0.01157	0.00957	0.01838	0.00693	0.00102	0.01371	0.01346	0.01621
0.01155	0.01005	0.01193	0.01131	0.00894	0.01441	0.0115	0.0109
0.01154	0.01697	0.02885	0.01743	0.01933	0.01832	0.02029	0.02296
0.01154	0.007	0.0077	0.01046	0.00804	0.0096	0.00579	0.00469
0.01152	0.01201	0.02059	0.01313	0.01352	0.01568	0.01313	0.01568
0.01151	0.00504	0.01533	0.0045	0.01038	0.00952	0.0052	0.01093
0.01148	0.03498	0.03397	0.01549	0.03798	0.02405	0.04084	0.02206
0.01146	0.01525	0.01938	0.01772	0.02494	0.03461	0.02597	0.02226
0.01146	0.00927	0.0155	0.00782	0.01374	0.01732	0.01341	0.00991
0.01145	0.0098	0.01357	0.01916	0.00843	0.00995	0.01248	0.01314
0.01143	0.00906	0.01583	0.01363	0.00848	0.00784	0.01115	0.00998
0.0114	0.00984	0.01163	0.00469	0.01023	0.01027	0.0078	0.01638
0.0114	0.009	0.00603	0.01244	0.00901	0.0117	0.00616	0.01173
0.0114	0.00742	0.01006	0.00268	0.02352	0.01028	0.00813	0.01042
0.01139	0.01525	0.02032	0.02451	0.02619	0.01953	0.01647	0.01754
0.01138	0.00984	0.01133	0.01269	0.00871	0.00891	0.00947	0.01288
0.01137	0.01119	0.01222	0.00876	0.00988	0.01172	0.01885	0.01749
0.01136	0.01782	0.02465	0.00888	0.01802	0.01626	0.02176	0.02228

0.01135	0.00837	0.03353	0.00785	0.03364	0.01759	0.03486	0.03235
0.01133	0.014	0.00982	0.01089	0.00638	0.00938	0.01451	0.01046
0.01132	0.00976	0.00749	0.00858	0.00884	0.01317	0.01058	0.014
0.01127	0.01142	0.01506	0.00866	0.00137	0.01831	0.01531	0.01826
0.01127	0.00871	0.00801	0.00995	0.00742	0.01002	0.01024	0.00585
0.01123	0.01129	0.01288	0.01726	0.02013	0.01383	0.02126	0.01681
0.01123	0.00513	0.01105	0.05301	0.01711	0.02223	0.00712	0.01616
0.01122	0.00651	0.01238	0.01335	0.00763	0.01032	0.00937	0.01014
0.0112	0.01165	0.0206	0.02094	0.02316	0.02408	0.01954	0.0243
0.0112	0.0111	0.01693	0.00791	0.01332	0.01267	0.01174	0.01198
0.01113	0.0129	0.01777	0.01389	0.02303	0.0221	0.01262	0.01848
0.01113	0.00565	0.00921	0.00873	0.01079	0.00447	0.00949	0.01202
0.01112	0.00864	0.01039	0.00779	0.01293	0.01547	0.00923	0.01175
0.01109	0.01396	0.01722	0.00965	0.02361	0.02132	0.02021	0.01639
0.01109	0.00862	0.01543	0.00738	0.01544	0.01344	0.01766	0.01146
0.01103	0.02259	0.02309	0.01489	0.01974	0.02401	0.03094	0.01548
0.01102	0.01647	0.02055	0.02247	0.02036	0.02322	0.0272	0.01762
0.01102	0.009	0.01549	0.01604	0.01404	0.01335	0.01523	0.01476
0.01101	0.02209	0.01455	0.01506	0.01879	0.0194	0.01452	0.00696
0.011	0.01156	0.02081	0.00769	0.01886	0.01822	0.01445	0.01396
0.01098	0.02105	0.02318	0.01796	0.01561	0.01739	0.01795	0.01739
0.01097	0.01061	0.01032	0.0068	0.01573	0.01829	0.01251	0.01358
0.01097	0.00602	0.0093	0.00997	0.00672	0.01004	0.00771	0.00853
0.01096	0.01168	0.01955	0.00477	0.01654	0.01688	0.01851	0.01808
0.01096	0.00844	0.02513	0.00248	0.01337	0.01284	0.0125	0.01022
0.01096	0.00642	0.01579	0.01774	0.01106	0.0085	0.01763	0.00629
0.01094	0.01307	0.01673	0.01299	0.01283	0.01425	0.01193	0.00449
0.0109	0.01675	0.0125	0.00858	0.02315	0.01445	0.00967	0.02451
0.0109	0.01278	0.01242	0.0108	0.01315	0.01437	0.01263	0.01586
0.01087	0.01394	0.03124	0.00855	0.04346	0.04062	0.05481	0.02406
0.01085	0.00634	0.00497	0.01236	0.01134	0.00638	0.01075	0.05304
0.01084	0.01035	0.00954	0.00445	0.02085	0.01382	0.00848	0.01507
0.01082	0.01177	0.01595	0.00316	0.02066	0.01961	0.01528	0.01416
0.01082	0.01767	0.02653	0.0269	0.0192	0.02894	0.02479	0.01125
0.01081	0.01215	0.006	0.01221	0.00777	0.00951	0.01498	0.01555
0.0108	0.01429	0.0101	0.01128	0.01689	0.01888	0.01559	0.02219
0.01079	0.00816	0.02259	0.00907	0.00965	0.0092	0.01393	0.01707
0.01079	0.00785	0.01288	0.01338	0.00731	0.00686	0.00886	0.00846
0.01078	0.00957	0.01144	0.00466	0.01153	0.00996	0.00847	0.00917
0.01076	0.00247	0.00457	0.01151	0.00638	0.01194	0.00298	0.00247
0.01072	0.01286	0.01322	0.00505	0.01183	0.01701	0.0167	0.01819
0.01072	0.00656	0.00494	0.00269	0.00675	0.00653	0.01444	0.00705
0.01071	0.01074	0.02094	0.00803	0.02276	0.02131	0.01259	0.02505
0.0107	0.03164	0.0167	0.01615	0.01381	0.02444	0.03032	0.02539
0.0107	0.00955	0.01553	0.01543	0.01235	0.01346	0.01563	0.01139
0.01069	0.01898	0.01817	0.00546	0.00656	0.01131	0.01708	0.0233
0.01068	0.01115	0.00969	0.01137	0.01201	0.01261	0.01254	0.01098
0.01065	0.01138	0.01601	0.01201	0.01032	0.00545	0.01319	0.0124
0.01063	0.00969	0.00817	0.00732	0.01017	0.00954	0.00777	0.00792

0.0106	0.00981	0.01084	0.01106	0.00974	0.0122	0.01488	0.01256
0.01058	0.00513	0.01488	0.00615	0.01069	0.01515	0.01098	0.01181
0.01058	0.01801	0.02773	0.01236	0.01809	0.0183	0.01659	0.00475
0.01055	0.01308	0.01717	0.01488	0.01849	0.01684	0.02281	0.02002
0.01055	0.01245	0.03097	0.01218	0.01539	0.02784	0.01985	0.01873
0.01051	0.02534	0.02584	0.015	0.03004	0.01827	0.03121	0.03053
0.01051	0.01638	0.01137	0.0058	0.00746	0.00995	0.00703	0.00957
0.01048	0.01145	0.01457	0.01679	0.01124	0.01514	0.0183	0.01951
0.01046	0.01102	0.01097	0.00506	0.00779	0.00676	0.0093	0.00751
0.01044	0.01224	0.00763	0.0134	0.01136	0.01118	0.00777	0.01083
0.01043	0.01768	0.02084	0.00553	0.02381	0.02228	0.02326	0.01718
0.01042	0.00865	0.01134	0.00304	0.02495	0.01836	0.01239	0.01008
0.01041	0.01388	0.01476	0.01189	0.01775	0.02538	0.01419	0.0129
0.01039	0.01143	0.00969	0.00722	0.01918	0.00774	0.01155	0.01411
0.01035	0.00498	0.01533	0.02456	0.01214	0.01198	0.00976	0.01098
0.01033	0.01504	0.02136	0.01482	0.01832	0.01294	0.01777	0.01453
0.0103	0.00643	0.00999	0.00389	0.01671	0.00985	0.01119	0.0132
0.01029	0.00431	0.00763	0.00514	0.00952	0.01	0.0047	0.01261
0.01026	0.01879	0.01826	0.00364	0.0175	0.01512	0.0148	0.01562
0.01026	0.00885	0.01451	0.00391	0.05036	0.02243	0.0064	0.01516
0.01024	0.00986	0.01598	0.00905	0.01433	0.01509	0.01289	0.01691
0.01024	0.01351	0.01022	0.0071	0.02141	0.01553	0.01391	0.01635
0.01023	0.00844	0.01275	0.01515	0.00938	0.00721	0.01173	0.01073
0.01023	0.00842	0.01088	0.0144	0.01121	0.01212	0.00712	0.00915
0.01023	0.00285	0.00165	0.00682	0.00183	0.00204	0.003	0.00369
0.01022	0.01562	0.0215	0.01698	0.01826	0.01977	0.00973	0.0122
0.0102	0.01222	0.01918	0.01222	0.01989	0.02199	0.01392	0.01489
0.0102	0.00685	0.01427	0.00043	0.00735	0.01865	0.01163	0.01376
0.01015	0.01621	0.01851	0.00873	0.01671	0.02073	0.01667	0.02162
0.01015	0.02216	0.00994	0.02256	0.03413	0.01571	0.0129	0.00637
0.01013	0.00983	0.0119	0.01969	0.01248	0.00981	0.01429	0.01361
0.01012	0.01346	0.01829	0.00649	0.01647	0.01365	0.01324	0.01036
0.01012	0.01141	0.01104	0.01112	0.00882	0.01366	0.01261	0.00727
0.0101	0.0109	0.0205	0.00217	0.00648	0.02131	0.00315	0.0176
0.0101	0.0144	0.0234	0.00492	0.01843	0.02184	0.01819	0.01556
0.01009	0.0098	0.01439	0.00509	0.04707	0.02217	0.01394	0.00683
0.01008	0.01718	0.01862	0.01312	0.01471	0.01377	0.0104	0.03109
0.01007	0.01106	0.01807	0.01402	0.02217	0.01719	0.01652	0.01739
0.01006	0.03476	0.00687	0.00622	0.03765	0.01093	0.0056	0.00615
0.01005	0.00449	0.0142	0.00147	0.018	0.01251	0.01661	0.0169
0.01002	0.01411	0.02839	0.00941	0.0478	0.03883	0.02654	0.03417
0.01002	0.00311	0.01053	0.00887	0.01078	0.01085	0.00432	0.00915
0.01002	0.0047	0.00722	0.00939	0.00746	0.00525	0.00583	0.00761
0.01001	0.00829	0.01157	0.03072	0.01001	0.00935	0.00606	0.01426
0.01001	0.00773	0.01017	0.00767	0.01161	0.01093	0.00633	0.01328
0.01001	0.00867	0.00951	0.00401	0.01056	0.01923	0.01186	0.01127
0.01001	0.00814	0.00802	0.00375	0.00831	0.01005	0.0111	0.00684
0.00999	0.02166	0.0103	0.01884	0.02878	0.01597	0.02373	0.01823
0.00996	0.01645	0.02217	0.01276	0.01908	0.01366	0.01384	0.02586

0.00996	0.00752	0.00706	0.01382	0.00786	0.00729	0.00569	0.00709
0.00995	0.0058	0.01584	0.00676	0.01358	0.01446	0.01455	0.01351
0.00993	0.0094	0.01067	0.00805	0.00642	0.01729	0.01833	0.01093
0.00991	0.01408	0.02317	0.005	0.02028	0.0188	0.01333	0.01503
0.00989	0.00566	0.01128	0.00163	0.01026	0.01	0.00406	0.01067
0.00987	0.0123	0.01264	0.01327	0.01408	0.01529	0.01154	0.01381
0.00986	0.02725	0.01154	0.00462	0.02917	0.00883	0.00565	0.01334
0.00986	0.00676	0.00952	0.00391	0.01628	0.01662	0.00977	0.00923
0.00985	0.00515	0.01685	0.00712	0.01331	0.0134	0.01536	0.01684
0.00985	0.00381	0.0048	0.00851	0.00665	0.00834	0.0053	0.00492
0.00984	0.01379	0.01564	0.00426	0.0221	0.01117	0.01274	0.01516
0.00983	0.00412	0.01105	0.01102	0.00627	0.00908	0.00747	0.00693
0.0098	0.01155	0.01158	0.01042	0.02108	0.01156	0.00916	0.00994
0.0098	0.01114	0.01001	0.00933	0.01075	0.00893	0.00904	0.00921
0.00979	0.00638	0.01013	0.0069	0.00782	0.01584	0.01126	0.00915
0.00978	0.01073	0.01088	0.00831	0.00929	0.01035	0.01055	0.0097
0.00976	0.0075	0.00785	0.00897	0.00635	0.0112	0.00904	0.00839
0.00975	0.01121	0.01183	0.0026	0.00747	0.01012	0.00658	0.00871
0.00973	0.01057	0.01243	0.00977	0.01076	0.00788	0.00925	0.01038
0.00971	0.00673	0.01466	0.00386	0.01091	0.01443	0.01058	0.02075
0.00971	0.01255	0.01502	0.02355	0.01339	0.01369	0.02306	0.01388
0.00971	0.00624	0.00867	0.01137	0.01016	0.01384	0.00168	0.01055
0.0097	0.00858	0.02133	0.00314	0.01457	0.03115	0.01643	0.0183
0.00966	0.00376	0.01314	0.00211	0.01163	0.0062	0.01484	0.01816
0.00966	0.00861	0.00455	0.01321	0.00746	0.01297	0.01269	0.01654
0.00963	0.00965	0.012	0.00552	0.02282	0.01398	0.00868	0.01218
0.00961	0.00501	8E-05	0.01248	0.01095	0.01284	0.01479	0.01227
0.00954	0.00489	0.00365	0.00802	0.00417	0.00368	0.00707	0.0078
0.00953	0.02294	0.03745	0.02118	0.01032	0.02238	0.02185	0.02113
0.00952	0.01677	0.01536	0.00443	0.02031	0.0191	0.01459	0.01591
0.00951	0.01658	0.01134	0.0115	0.02513	0.01896	0.02103	0.02363
0.00951	0.00781	0.01422	0.01464	0.02174	0.01968	0.01815	0.01249
0.00951	0.01732	0.01849	0.01825	0.01366	0.02255	0.01894	0.01045
0.00948	0.00756	0.01002	0.00304	0.02198	0.01824	0.02461	0.00291
0.00947	0.01647	0.02287	0.00968	0.02084	0.01919	0.01671	0.00577
0.00946	0.01116	0.01032	0.01564	0.00878	0.00726	0.01427	0.01325
0.00945	0.00771	0.01055	0.01069	0.00743	0.00786	0.00485	0.02827
0.00942	0.01096	0.01312	0.00254	0.01307	0.01321	0.00995	0.02494
0.00941	0.00765	0.00919	0.01197	0.00999	0.01195	0.01148	0.01307
0.00938	0.01989	0.0239	0.02605	0.0155	0.021	0.01899	0.01957
0.00938	0.013	0.0177	0.00682	0.01496	0.01546	0.01923	0.01464
0.00938	0.00861	0.00569	0.01006	0.00598	0.00697	0.00597	0.00687
0.00929	0.02043	0.03656	0.02166	0.01976	0.02186	0.02	0.01708
0.00928	0.01348	0.01724	0.00638	0.01445	0.0092	0.01747	0.01239
0.00928	0.00576	0.00653	0.02007	0.00719	0.00933	0.00919	0.00815
0.00927	0.01544	0.01811	0.00267	0.03192	0.02947	0.02525	0.01941
0.00927	0.00791	0.01363	0.00198	0.02833	0.02057	0.02154	0.0133
0.00927	0.00926	0.01516	0.00497	0.00837	0.01113	0.00708	0.00873
0.00925	0.0157	0.01977	0.00991	0.01314	0.01592	0.01848	0.014

0.00925	0.01558	0.01878	0.00341	0.00221	0.00177	0.00185	0.00336
0.00924	0.0115	0.01565	0.01787	0.01632	0.01189	0.01254	0.00658
0.00923	0.01338	0.01081	0.00826	0.00649	0.00695	0.01609	0.00758
0.00923	0.00719	0.01219	0.01783	0.01793	0.00945	0.00972	0.00484
0.0092	0.0048	0.01814	0.01217	0.02382	0.00943	0.02744	0.03379
0.00917	0.00477	0.01351	0.01135	0.01038	0.01244	0.00935	0.01001
0.00916	0.00981	0.01119	0.00431	0.0113	0.0118	0.01328	0.01475
0.00916	0.00803	0.01075	0.00626	0.02366	0.00832	0.00969	0.00872
0.00916	0.0064	0.0066	0.0048	0.00138	0.00504	0.00917	0.00857
0.00914	0.01278	0.00966	0.00467	0.0254	0.01288	0.01075	0.00789
0.00912	0.01436	0.00864	0.01256	0.00768	0.01028	0.01503	0.01026
0.00911	0.00838	0.0094	0.00396	0.00824	0.00895	0.00598	0.0049
0.0091	0.01209	0.01684	0.00781	0.01337	0.01588	0.00764	0.01379
0.00908	0.00608	0.00927	0.0141	0.00998	0.01189	0.01176	0.01055
0.00904	0.00729	0.01895	0.00808	0.01167	0.00884	0.01493	0.00849
0.00903	0.02146	0.02634	0.0082	0.0293	0.03569	0.03738	0.01438
0.009	0.01121	0.00927	0.00793	0.01518	0.01338	0.01433	0.00765
0.00899	0.00819	0.01124	0.00347	0.00981	0.01028	0.00768	0.00796
0.0089	0.0137	0.01455	0.0091	0.00985	0.01067	0.01181	0.01027
0.00889	0.00989	0.01055	0.00562	0.00961	0.00991	0.00693	0.00785
0.00888	0.00928	0.00695	0.00185	0.05321	0.01768	0.00707	0.00627
0.00888	0.01157	0.00679	0.0068	0.01685	0.00482	0.01297	0.00314
0.00886	0.01137	0.01618	0.00771	0.01736	0.01005	0.01266	0.019
0.00882	0.00552	0.00274	0.01941	0.03246	0.01457	0.00951	0.00622
0.00881	0.00897	0.01107	0.00806	0.01702	0.01449	0.00906	0.00888
0.00879	0.00735	0.02006	0.00453	0.03088	0.01446	0.00858	0.01077
0.00878	0.00629	0.0059	0.00251	0.01717	0.00174	0.00882	0.00413
0.00875	0.01441	0.00884	0.00484	0.02224	0.00998	0.01209	0.01869
0.00875	0.01155	0.01599	0.00631	0.0165	0.00909	0.00428	0.00793
0.00874	0.01196	0.00737	0.00253	0.0282	0.01094	0.005	0.0018
0.00872	0.00669	0.00648	0.00511	0.00417	0.00866	0.00525	0.00822
0.00872	0.0055	0.0073	0.01178	0.00689	0.00459	0.00585	0.00655
0.00869	0.00904	0.0133	0.01016	0.01479	0.01257	0.01392	0.01448
0.00868	0.00239	0.00593	0.00972	0.00358	0.01006	0.00541	0.00854
0.00867	0.00718	0.0054	0.01105	0.0101	0.01278	0.01032	0.00931
0.00865	0.01076	0.00794	0.00393	0.03388	0.01415	0.01089	0.02002
0.00864	0.0203	0.01373	0.00154	0.01702	0.01118	0.01279	0.01364
0.00861	0.00482	0.03684	0.0035	0.0191	0.01719	0.01755	0.01215
0.00859	0.01201	0.01273	0.00605	0.01311	0.0152	0.0124	0.0134
0.00855	0.00675	0.01289	0.00734	0.0062	0.00896	0.00708	0.01761
0.00855	0.00315	0.00403	0.00708	0.00727	0.00643	0.0016	0.00519
0.00854	0.00507	0.01107	0.00976	0.00595	0.00697	0.01103	0.00714
0.00853	0.00534	0.00583	0.00788	0.00919	0.00801	0.00869	0.00621
0.00852	0.01013	0.02005	0.00944	0.0176	0.01919	0.01891	0.01023
0.00851	0.01047	0.0174	0.00806	0.01725	0.01409	0.01177	0.0124
0.0085	0.00707	0.01069	0.0055	0.00876	0.00751	0.00565	0.00815
0.00849	0.01134	0.01466	0.00159	0.01341	0.01199	0.01121	0.01003
0.00845	0.01187	0.0342	0.04837	0.04106	0.02678	0.01666	0.01976
0.00844	0.01513	0.0234	0.00916	0.00884	0.01142	0.01141	0.00846

0.00843	0.01878	0.02023	0.00906	0.01517	0.01357	0.01443	0.00555
0.00841	0.00793	0.00814	0.00527	0.00523	0.00795	0.01026	0.00944
0.00841	0.00746	0.00363	0.01247	0.0127	0.00672	0.00725	0.00665
0.0084	0.0071	0.00891	0.00458	0.00678	0.00807	0.00617	0.00103
0.00838	0.01196	0.0221	0.01441	0.0218	0.02808	0.02418	0.02768
0.00838	0.00373	0.00726	0.00647	0.00672	0.0049	0.00567	0.01406
0.00838	0.00673	0.01205	0.00638	0.01966	0.0149	0.00615	0.0132
0.00838	0.00934	0.01045	0.01745	0.00952	0.01083	0.00804	0.00888
0.00838	0.00778	0.00764	0.00589	0.00994	0.00128	0.00924	0.00801
0.00838	0.00541	0.00812	0.00379	0.00709	0.00557	0.00643	0.00525
0.00837	0.01275	0.01307	0.01151	0.02186	0.01381	0.01242	0.01651
0.00835	0.00302	0.00252	0.00243	0.00943	0.00839	0.00212	0.00454
0.00834	0.00861	0.00483	0.00183	0.00899	0.00969	0.0078	0.0113
0.00834	0.0089	0.00561	0.00559	0.00418	0.00917	0.00192	0.0084
0.00832	0.01634	0.01936	0.00232	0.03797	0.02046	0.01907	0.02092
0.00829	0.01157	0.01577	0.01031	0.0107	0.01373	0.01037	0.011
0.00827	0.00443	0.0048	0.01111	0.0056	0.00958	0.00685	0.00807
0.00824	0.00652	0.02064	0.00293	0.05235	0.04355	0.0446	0.01117
0.00819	0.01589	0.00557	0.00381	0.01801	0.00725	0.01078	0.01257
0.00818	0.00851	0.01233	0.00592	0.01466	0.00745	0.00485	0.00615
0.00815	0.00533	0.00686	0.00688	0.00635	0.00761	0.00595	0.00519
0.00815	0.00539	0.00817	0.00833	0.00231	0.00959	0.00751	0.003
0.00815	0.00644	0.0043	0.00464	0.00154	0.00927	0.00826	0.00203
0.00814	0.00967	0.01679	0.00647	0.01604	0.01518	0.01756	0.01357
0.00814	0.00688	0.01109	0.0125	0.00862	0.00602	0.00705	0.00659
0.00811	0.00677	0.0034	0.00112	0.005	0.00335	0.00169	0.01133
0.00811	0.00699	0.00746	0.00582	0.00633	0.00724	0.00758	0.01006
0.00809	0.01185	0.0095	0.00875	0.00595	0.01101	0.00682	0.00657
0.00807	0.01226	0.01143	0.00191	0.01278	0.01553	0.01915	0.01583
0.00807	0.01519	0.00887	0.00428	0.02029	0.01072	0.01325	0.00484
0.00805	0.00905	0.01366	0.0047	0.0109	0.01226	0.01014	0.0142
0.00805	0.00719	0.00645	0.01168	0.012	0.0075	0.00608	0.0127
0.00801	0.01114	0.00727	0.00899	0.00482	0.0051	0.00574	0.01221
0.00796	0.01489	0.02264	0.00876	0.02392	0.02415	0.0244	0.01776
0.00796	0.0085	0.01377	0.00984	0.01217	0.01316	0.01072	0.01757
0.00796	0.00594	0.01017	0.00905	0.00716	0.00335	0.00093	0.00868
0.00795	0.00424	0.00799	0.00817	0.0075	0.00504	0.00514	0.00646
0.00794	0.00973	0.00664	0.00558	0.00615	0.00987	0.00294	0.0054
0.00793	0.00578	0.00622	0.00854	0.00642	0.00779	0.0023	0.00507
0.00787	0.01409	0.01877	0.01344	0.01015	0.01406	0.01807	0.01678
0.00787	0.01957	0.02442	0.00651	0.01767	0.00516	0.02132	0.0152
0.00786	0.00716	0.00192	0.00312	0.00938	0.00723	0.00737	0.00301
0.00783	0.01108	0.0158	0.00698	0.02752	0.02266	0.01543	0.01995
0.00782	0.01196	0.0113	0.00127	0.01444	0.0122	0.00997	0.01571
0.00779	0.00951	0.0103	0.00434	0.01215	0.00856	0.01073	0.00837
0.00778	0.00506	0.00637	0.00297	0.0051	0.00745	0.00725	0.00886
0.00777	0.00832	0.01611	0.01076	0.0155	0.01593	0.0095	0.01285
0.00776	0.00764	0.00892	0.00333	0.00927	0.00938	0.00759	0.01022
0.00776	0.01249	0.01543	0.00774	0.01703	0.01744	0.02189	0.0099

0.00775	0.00973	0.01083	0.01094	0.00819	0.01153	0.0164	0.01271
0.00775	0.01099	0.01618	0.00622	0.01479	0.01582	0.0226	0.00808
0.00775	0.0073	0.01243	0.00438	0.00956	0.00531	0.00457	0.00603
0.00773	0.01683	0.00952	0.00596	0.01475	0.00719	0.00801	0.00715
0.00772	0.0125	0.01044	0.00359	0.01522	0.01498	0.01538	0.01087
0.00772	0.00635	0.00885	0.00012	0.01244	0.00691	0.01037	0.01038
0.00771	0.00739	0.01226	0.00837	0.00901	0.00588	0.00119	0.00702
0.00768	0.00427	0.00685	0.00148	0.00812	0.00447	0.00633	0.01786
0.00768	0.00554	0.0095	0.00312	0.00777	0.01053	0.00684	0.00495
0.00768	0.0274	0.00467	0.0029	0.02273	0.00701	0.00216	0.00438
0.00767	0.00444	0.00601	0.00607	0.01046	0.00514	0.00888	0.00697
0.00762	0.0083	0.00919	0.00432	0.00837	0.0157	0.00889	0.01182
0.00759	0.00488	0.01359	0.00613	0.00747	0.01605	0.01	0.00985
0.00757	0.00929	0.00909	0.00675	0.00833	0.00931	0.01389	0.0131
0.00756	0.01173	0.02226	0.01056	0.01796	0.01393	0.04152	0.01965
0.00754	0.00845	0.0109	0.00432	0.00717	0.00522	0.00972	0.00891
0.00753	0.00949	0.00605	0.0078	0.01112	0.01003	0.00818	0.00392
0.00752	0.00629	0.00763	0.00354	0.00447	0.00792	0.00942	0.01375
0.00752	0.00636	0.0087	0.00333	0.0261	0.01776	0.03372	0.00908
0.00751	0.01713	0.0234	0.02122	0.01829	0.03132	0.01209	0.02216
0.00751	0.01456	0.00969	0.00601	0.02509	0.01059	0.01647	0.01251
0.00748	0.01478	0.01731	0.00761	0.01636	0.01931	0.02234	0.01702
0.00748	0.00768	0.0026	0.00593	0.00623	0.00178	0.00399	0.00196
0.00746	0.01201	0.00599	0.00914	0.01685	0.01112	0.01328	0.01071
0.00744	0.00432	0.01226	0.00314	0.03917	0.0289	0.00983	0.00367
0.0074	0.00952	0.02058	0.02158	0.00813	0.01613	0.00995	0.0139
0.00738	0.00543	0.00679	0.00416	0.00049	0.00825	0.01043	0.0054
0.00734	0.00535	0.00415	0.00474	0.00193	0.00863	0.00423	0.00053
0.00733	0.00581	0.00909	0.00748	0.00893	0.01196	0.00265	0.00866
0.00732	0.00484	0.00665	0.01524	0.00825	0.01467	0.01403	0.01233
0.00731	0.00611	0.01237	0.00816	0.00797	0.01227	0.00749	0.00883
0.00731	0.00166	0.00321	0.00052	0.01156	0.00706	0.00568	0.0031
0.0073	0.01082	0.00421	0.00362	0.02029	0.00697	0.00919	0.01325
0.00729	0.00868	0.02401	0.00535	0.01792	0.02182	0.01749	0.01703
0.00729	0.00631	0.00439	0.00268	0.00566	0.0107	0.00248	0.00698
0.00727	0.01877	0.01158	0.01389	0.01222	0.01852	0.00993	0.01552
0.00727	0.01109	0.02834	0.01065	0.02463	0.0157	0.03099	0.01417
0.00725	0.01068	0.01044	0.00625	0.01097	0.00936	0.01205	0.00537
0.00722	0.01375	0.01439	0.00793	0.01609	0.00551	0.01242	0.00614
0.00721	0.00524	0.00517	0.00307	0.01357	0.0059	0.01118	0.02754
0.00719	0.00799	0.0117	0.00918	0.01354	0.01099	0.00903	0.01491
0.00718	0.00922	0.00643	0.00826	0.02601	0.02202	0.02306	0.02023
0.00712	0.02578	0.01431	0.00682	0.00725	0.01142	0.005	0.01059
0.00712	0.00444	0.02394	0.00676	0.00546	0.00884	0.00391	0.00654
0.00712	0.0061	0.00613	0.00457	0.00559	0.00739	0.00365	0.00576
0.00711	0.01061	0.02571	0.00512	0.0238	0.01414	0.01703	0.00682
0.0071	0.00812	0.00617	0.00669	0.00673	0.00737	0.00853	0.00842
0.0071	0.00778	0.00728	0.00743	0.01079	0.012	0.00298	0.00829
0.00709	0.00785	0.00923	0.00737	0.00702	0.01074	0.00807	0.00742

0.00706	0.00437	0.00662	0.0027	0.00674	0.00333	0.00564	0.00059
0.00705	0.00686	0.0089	0.00621	0.00992	0.01064	0.0096	0.01041
0.00704	0.00225	0.0069	0.01304	0.00604	0.0057	0.00777	0.00515
0.00701	0.00441	0.00566	0.00715	0.00235	0.00448	0.00259	0.01109
0.00694	0.00713	0.0107	0.00852	0.00955	0.00676	0.00472	0.00109
0.00693	0.00773	0.0041	0.00112	0.01388	0.0104	0.00492	0.00905
0.00692	0.0143	0.00704	0.01668	0.01196	0.01912	0.01703	0.01239
0.00692	0.00426	0.00682	0.02814	0.0101	0.01041	0.00158	0.01114
0.00691	0.00336	0.01275	0.00979	0.00518	0.00793	0.01011	0.01456
0.0069	0.00216	0.001	0.00242	0.00171	0.00314	0.00194	0.00434
0.00689	0.00431	0.00588	0.00383	0.00462	0.008	0.00633	0.00571
0.00687	0.0067	0.02606	0.00716	0.0217	0.01736	0.02359	0.01738
0.00686	0.01648	0.01459	0.01235	0.01343	0.00832	0.01164	0.01524
0.00685	0.01075	0.00615	0.00694	0.00313	0.00866	0.01018	0.013
0.00685	0.00439	0.00567	0.0045	0.00678	0.0079	0.00503	0.01209
0.00685	0.00647	0.00316	0.00238	0.01231	0.01455	0.00721	0.00619
0.00683	0.01029	0.01119	0.00924	0.00946	0.01209	0.01122	0.0083
0.00682	0.01141	0.01856	0.01176	0.02408	0.02468	0.03076	0.01673
0.00681	0.0066	0.00382	0.00393	0.00445	0.00442	0.00603	0.0077
0.00681	0.00147	0.00212	0.00183	0.00554	0.01195	0.00888	0.00135
0.00679	0.01455	0.00909	0.00366	0.01103	0.00599	0.01271	0.00192
0.00678	0.00562	0.00558	0.00281	0.00939	0.00735	0.00552	0.00373
0.00676	0.00809	0.01376	0.00231	0.01249	0.01115	0.00288	0.00987
0.00676	0.00447	0.01175	0.00426	0.00689	0.00483	0.00524	0.00274
0.00674	0.01348	0.00949	0.01097	0.01666	0.02609	0.01853	0.01549
0.00671	0.0108	0.03064	0.01455	0.00352	0.01594	0.00642	0.00751
0.00668	0.01676	0.01537	0.00488	0.01363	0.01977	0.01826	0.02247
0.00667	0.00557	0.00393	0.00304	0.00843	0.01327	0.00225	0.01158
0.00667	0.01206	0.00636	0.00299	0.00702	0.00541	0.00639	0.00635
0.00665	0.00327	0.00021	0.02066	0.01447	0.0191	0.00608	0.02406
0.00665	0.01126	0.00782	0.00731	0.01191	0.02109	0.0303	0.02005
0.00658	0.00829	0.01769	0.00868	0.00836	0.01006	0.00768	0.0106
0.00658	0.00744	0.00508	0.0056	0.00686	0.01275	0.01265	0.00454
0.00654	0.00476	0.00595	0.00911	0.00892	0.00756	0.00635	0.00479
0.00652	0.00694	0.00641	0.01	0.00905	0.01046	0.00999	0.00495
0.00651	0.00964	0.02838	0.00133	0.00474	0.01478	0.00736	0.02219
0.00651	0.00917	0.01245	0.00058	0.01193	0.01042	0.00733	0.00931
0.00649	0.01374	0.0065	0.0079	0.0217	0.01001	0.02327	0.02394
0.00646	0.00285	0.01346	0.01254	0.0097	0.01086	0.01369	0.01284
0.00642	0.00671	0.01365	0.00601	0.01207	0.0116	0.016	0.00799
0.00639	0.00443	0.0079	0.00527	0.0057	0.00704	0.00125	0.00419
0.00637	0.00372	0.01063	0.00698	0.01297	0.01652	0.01606	0.01646
0.00634	0.00457	0.01222	0.02301	0.01035	0.00819	0.004	0.0103
0.00631	0.00734	0.00387	0.00251	0.01309	0.00755	0.00561	0.00534
0.00629	0.0049	0.01195	0.01383	0.0056	0.00831	0.00829	0.01282
0.00628	0.00291	0.00637	0.00914	0.00654	0.0071	0.00867	0.00685
0.00627	0.00449	0.0081	0.00653	0.00603	0.00349	0.00472	0.00548
0.00625	0.01681	0.00424	0.01221	0.01362	0.01676	0.01349	0.01043
0.00624	0.00349	0.00391	0.00543	0.00753	0.00621	0.00323	0.00411

0.00622	0.00456	0.00605	0.00295	0.0049	0.00631	0.013	0.00968
0.00621	0.00284	0.00565	0.00386	0.00449	0.00883	0.00428	0.00554
0.00619	0.00492	0.00283	0.00911	0.00515	0.00576	0.00055	0.00393
0.00618	0.00963	0.01329	0.00395	0.01048	0.01201	0.0087	0.00691
0.00615	0.00472	0.01411	0.0076	0.01542	0.01102	0.01111	0.01193
0.00615	0.01123	0.01425	0.00216	0.00743	0.00837	0.00752	0.01162
0.00615	0.00537	0.00614	0.01188	0.00806	0.01133	0.00901	0.00916
0.00615	0.00649	0.00876	0.00317	0.02811	0.00905	0.00247	0.00413
0.00614	0.00834	0.00313	0.00513	0.00115	0.0113	0.00661	0.01213
0.00612	0.00452	0.01003	0.00186	0.02321	0.01326	0.01791	0.02025
0.00608	0.00094	0.00883	0.005	0.00679	0.00338	0.002	0.00548
0.00607	0.02017	0.00615	0.00195	0.01628	0.00661	0.01248	0.01002
0.00601	0.00914	0.01091	0.01942	0.01015	0.00938	0.01245	0.00771
0.00601	0.00419	0.00399	0.00688	0.00736	0.0052	0.00485	0.00486
0.006	0.00545	0.01765	0.00551	0.0126	0.00615	0.00849	0.0125
0.006	0.00317	0.00408	0.00392	0.00831	0.00893	0.00385	0.00872
0.006	0.0096	0.01484	0.00552	0.01765	0.01346	0.00811	0.00782
0.00599	0.00981	0.00496	0.00282	0.00397	0.00743	0.00297	0.00452
0.00599	0.00172	0.00121	0.00076	0.00394	0.00195	0.00099	0.0018
0.00596	0.01219	0.01243	0.00233	0.01814	0.01426	0.0172	0.01103
0.00596	0.01775	0.0122	0.00306	0.01468	0.00806	0.00408	0.00686
0.00596	0.00517	0.00381	0.00043	0.00302	0.00421	0.00204	0.00255
0.00595	0.00669	0.0045	0.00157	0.00609	0.00911	0.00638	0.00844
0.00592	0.01262	0.01212	0.00644	0.03027	0.01158	0.0069	0.00911
0.00587	0.03486	0.03654	0.00726	0.04401	0.03908	0.04139	0.04121
0.00583	0.00406	0.00472	0.00338	0.00522	0.00395	0.00785	0.00535
0.00578	0.00466	0.00626	0.00354	0.03391	0.01629	0.00753	0.0066
0.00577	0.00795	0.01344	0.0081	0.00741	0.01422	0.00445	0.01436
0.00574	0.00362	0.00612	0.00485	0.00262	0.00383	0.01025	0.00321
0.00572	0.01604	0.00442	0.00315	0.01116	0.01803	0.00906	0.0218
0.00566	0.01885	0.00397	0.00346	0.00413	0.00326	0.00558	0.00703
0.0056	0.00822	0.00766	0.00158	0.01491	0.00978	0.01149	0.00953
0.00559	0.00372	0.00532	0.01045	0.00197	0.00931	0.00757	0.00155
0.00557	0.00212	0.01149	0.01138	0.0061	0.00279	0.0035	0.01021
0.00553	0.00579	0.00924	0.00108	0.00646	0.00722	0.00522	0.00686
0.00551	0.0055	0.00427	0.00197	0.00344	0.00617	0.0016	0.00146
0.0055	0.00338	0.00306	0.00276	0.00965	0.0045	0.0028	0.00471
0.00549	0.00308	0.00323	0.00566	0.00604	0.00716	0.00542	0.00247
0.00548	0.00576	0.00245	0.00599	0.00788	0.00653	0.00413	0.00831
0.00546	0.00785	0.00766	0.00562	0.01104	0.00628	0.00852	0.00221
0.00545	0.00964	0.00325	0.00295	0.00373	0.00187	0.00359	0.00273
0.0054	0.00698	0.00611	0.00322	0.00941	0.01016	0.00768	0.00524
0.00538	0.00837	0.00535	0.0026	0.0105	0.00899	0.00618	0.0123
0.00536	0.00772	0.00587	0.01187	0.00796	0.0071	0.01187	0.00845
0.00534	0.00515	0.00479	0.00112	0.00623	0.00328	0.00126	0.00317
0.00531	0.01035	0.00591	0.00542	0.01492	0.00892	0.01453	0.01221
0.00531	0.00746	0.00678	0.00347	0.02077	0.01618	0.00402	0.01116
0.00531	0.01547	0.00156	0.00234	0.01299	0.00336	0.00162	0.01109
0.00531	0.00521	0.00737	0.00123	0.00751	0.00767	0.00467	0.00685

0.00525	0.00444	0.00692	0.00654	0.0049	0.00418	0.00864	0.00492
0.00522	0.01434	0.01348	0.00026	0.01568	0.00398	0.01178	0.01344
0.0052	0.00526	0.00297	0.00387	0.00618	0.01107	0.00926	0.00636
0.0052	0.00892	0.00459	0.00803	0.01106	0.01142	0.01008	0.00138
0.00519	0.00924	0.02068	0.00534	0.01941	0.01631	0.02098	0.01385
0.00518	0.00421	0.0032	0.01165	0.00693	0.00678	0.0036	0.01226
0.00516	0.0051	0.0072	0.00811	0.00622	0.00715	0.00561	0.00217
0.00502	0.00478	0.00467	0.00938	0.00585	0.00706	0.00781	0.00941
0.00502	0.00665	0.01478	0.00422	0.0072	0.00389	0.01013	0.0072
0.00501	0.01041	0.0072	0.01394	0.01122	0.00866	0.00766	0.00732
0.00493	0.00585	0.00789	0.00201	0.00525	0.00534	0.00521	0.00687
0.0049	0.00244	0.00586	0.00252	0.00201	0.00305	0.00544	0.00992
0.0049	0.00324	0.00688	0.00092	0.00468	0.00519	0.00356	0.00445
0.00488	0.00324	0.00543	0.00492	0.00669	0.00438	0.00459	0.00414
0.00485	0.01238	0.00848	0.00819	0.00621	0.0111	0.00804	0.0021
0.00483	0.00455	0.0076	0.0056	0.00638	0.00725	0.00117	0.01177
0.00481	0.00779	0.01982	0.00226	0.00836	0.01929	0.00241	0.01484
0.00481	0.00901	0.00866	0.00584	0.0075	0.01581	0.00912	0.00742
0.0048	0.0091	0.00586	0.00201	0.00895	0.00463	0.0068	0.00763
0.00475	0.00464	0.00482	0.00139	0.00682	0.00785	0.00616	0.00602
0.00473	0.02234	0.00447	0.00492	0.02651	0.00271	0.00637	0.00527
0.00467	0.00161	0.00923	0.006	0.01734	0.01031	0.02954	0.00694
0.00466	0.00583	0.0022	0.01538	0.01073	0.0019	0.00659	0.0086
0.00464	0.01382	0.01231	0.01001	0.01621	0.01439	0.00924	0.00533
0.00459	0.00995	0.00463	0.00769	1E-05	0.00458	0.01059	0.00742
0.00459	0.00472	0.00319	0.00157	0.00492	0.00711	0.00195	0.00483
0.00458	0.00462	0.00336	0.0042	0.0024	0.0102	0.00582	0.00211
0.00453	0.00957	0.00508	0.00243	0.0104	0.00542	0.0047	0.00393
0.00453	0.0031	0.00218	0.00353	0.00229	0.00486	0.00258	0.00202
0.00452	0.00701	0.00418	0.00838	0.00511	0.00485	0.00811	0.01369
0.0045	0.00586	0.0067	0.00255	0.01618	0.0081	0.0072	0.00594
0.00446	0.00197	0.006	0.00105	0.0044	0.00156	0.00939	0.00832
0.00443	0.00448	0.00718	0.00623	0.00854	0.00631	0.00615	0.00607
0.00442	0.00827	0.00528	0.00461	0.00255	0.00323	0.00605	0.00545
0.00441	0.00711	0.00436	0.00412	0.00338	0.00283	0.00328	0.00426
0.0044	0.01647	0.0044	0.00931	0.00427	0.00319	0.00776	0.01875
0.0044	0.00315	0.00509	0.0045	0.00487	0.00891	0.00646	0.00563
0.00438	0.00653	0.01629	0.00476	0.00752	0.00238	0.01714	0.00265
0.00438	0.00577	0.00627	0.00485	0.00522	0.00521	0.00541	0.00119
0.00435	0.00296	0.00373	0.00265	0.00424	0.00704	0.00199	0.00037
0.00434	0.01135	0.01271	0.00919	0.0143	0.0194	0.00743	0.01054
0.00434	0.00231	0.00222	0.00174	0.00051	0.00392	0.00204	0.0063
0.00431	0.00588	0.0079	0.00532	0.00535	0.00498	0.00234	0.00614
0.00428	0.00804	0.01163	0.0016	0.00958	0.00989	0.00687	0.00699
0.00423	0.00364	0.00258	0.0051	0.00628	0.00522	0.01397	0.01773
0.00421	0.00179	0.00485	0.00528	0.00407	0.00709	0.00656	0.00836
0.00421	0.00874	0.01143	0.00294	0.02592	0.00992	0.00801	0.00724
0.00416	0.00098	0.00957	0.00761	0.00427	0.00779	0.00617	0.00752
0.00415	0.00371	0.0018	0.00323	0.00375	0.00714	0.00257	0.00938

0.00415	0.00575	0.00294	0.00352	0.00944	0.00352	0.0023	0.00546
0.00414	0.01252	0.00368	0.00742	0.00786	0.00963	0.00602	0.00233
0.00413	0.0034	0.01206	0.00644	0.00639	0.00958	0.00844	0.00709
0.00407	0.00295	0.00462	0.00156	0.00836	0.00467	0.00455	0.01327
0.00407	0.00764	0.01838	0.00596	0.01683	0.00681	0.01441	0.0088
0.00406	0.00636	0.0052	0.00277	0.00996	0.01865	0.0099	0.01426
0.00404	0.00913	0.00597	0.0005	0.01856	0.00597	0.00816	0.00814
0.00404	0.00219	0.00139	0.00254	0.00162	0.0025	0.00138	0.00228
0.00403	0.00371	0.01279	0.00571	0.00904	0.01378	0.00908	0.00921
0.00401	0.0137	0.01293	0.00817	0.01169	0.01276	0.01357	0.01029
0.00398	0.00355	0.00641	0.00271	0.00428	0.00853	0.00814	0.00437
0.00389	0.00347	0.00104	0.00194	0.00145	0.00808	0.00714	0.00384
0.00383	0.00842	0.01421	0.00487	0.01229	0.00993	0.01074	0.0096
0.00381	0.01613	0.00935	0.02385	0.01134	0.02149	0.01793	0.01359
0.00379	0.01286	0.00833	0.00054	0.00531	0.00683	0.00853	0.00587
0.00372	0.00547	0.00763	0.00027	0.00968	0.00606	0.00829	0.00583
0.00369	0.00395	0.01518	0.00476	0.00968	0.01222	0.00565	0.00688
0.00366	0.00266	0.0071	0.00602	0.00678	0.00877	0.00667	0.00577
0.00364	0.01772	0.01548	0.01607	0.01697	0.01425	0.00321	0.00663
0.00364	0.00563	0.00339	0.00405	0.00165	0.00518	0.00211	0.00427
0.00359	0.00927	0.01828	0.00713	0.00632	0.01195	0.00665	0.01173
0.00357	0.00713	0.00851	0.00526	0.00356	0.00286	0.00684	0.00536
0.0035	0.00232	0.00378	0.00625	0.00126	0.00397	0.00051	0.0024
0.00347	0.00235	0.00747	0.00032	0.01272	0.00321	0.00214	0.00582
0.00343	0.00911	0.00632	0.00289	0.00523	0.0042	0.00589	0.00499
0.00334	0.00336	0.02302	0.00624	0.00848	0.02648	0.00681	0.0082
0.00331	0.00635	0.00854	0.00216	0.00843	0.00816	0.00654	0.00721
0.0033	0.00523	0.00478	0.00081	0.00485	0.00986	0.00322	0.00358
0.00324	0.00319	0.02019	0.00355	0.00799	0.00729	0.00626	0.00817
0.00319	0.00271	0.00643	0.0057	0.00635	0.00921	0.00734	0.00601
0.00316	0.01504	0.00965	0.01366	0.01174	0.01108	0.01069	0.01
0.00315	0.00306	0.00695	0.00044	0.00764	0.00572	0.0042	0.00508
0.00315	0.00496	0.01461	0.00279	0.02236	0.01321	0.00776	0.00401
0.00314	0.00625	0.00603	0.00263	0.02155	0.00564	0.00317	0.00349
0.00313	0.00386	0.00415	0.00227	0.00563	0.00454	0.00241	0.00086
0.00312	0.0105	0.01886	0.00353	0	0.00549	0.00261	0.00074
0.00305	0.00543	0.00906	0.00241	0.01242	0.01177	0.0114	0.01303
0.00302	0.00528	0.00448	0.0007	0.01576	0.00787	0.00593	0.00272
0.00296	0.00135	0.00115	0.00145	0.00105	0.0037	0.00307	0.00015
0.00291	0.0046	0.00383	0.00391	0.00549	0.00307	0.0023	0.00137
0.00284	0.00777	0.00459	0.00476	0.00665	0.00556	0.00493	0.00408
0.00273	0.00151	0.00111	0.00136	0.0029	0.00388	0.00105	0.00366
0.00271	0.00447	0.00171	0.00802	0.00247	0.00457	0.00709	0.01861
0.0027	0.00399	0.01464	0.00045	0.00068	0.0078	0.0036	0.00849
0.00269	0.00288	0.0014	0.00258	0.00532	0.0044	0.00097	0.00347
0.00267	0.00504	0.00244	0.00409	0.01603	0.00125	0.00459	0.00178
0.00265	0.00313	0.00137	0.00071	0.00411	0.00497	0.00192	0.0107
0.00258	0.00554	0.01384	0.0015	0.01472	0.01353	0.01573	0.00893
0.00256	0.00697	0.00629	0.00637	0.01003	0.00761	0.00922	0.01469

0.00254	0.00099	0.00078	0.00398	0.00137	0.002	0.00229	0.00237
0.0025	0.00012	0.0045	0.00201	0.00357	0.00169	0.00051	0.0002
0.00246	0.00227	0.00281	0.00784	0.00148	0.00186	0.00082	0.00373
0.00242	0.0075	0.00471	0.00099	0.00305	0.00331	0.00186	0.00177
0.00238	0.01328	0.01765	0	0.02128	0.00752	0.01897	0.02018
0.00236	0.00048	0.00411	0.00354	0.00189	0.00165	0.0014	0.00632
0.00225	0.00307	0.00275	0.00192	0.00092	0.00194	0.00113	0.00298
0.00209	0.00504	0.00245	0.00175	0.01682	0.00734	0.00254	0.00511
0.00206	0.00351	0.0016	0.00174	0.00535	0.00128	0.00113	0.00021
0.00198	0.00141	0.00182	0.00409	0.00063	0.00118	0.00121	0.00088
0.00197	0.00601	0.00575	0.00435	0.00658	0.00476	0.00451	0.0052
0.00172	0.00103	0.00263	0.00353	0.00366	0.00362	0.0014	0.00146
0.00154	0.0039	0.00209	0.00313	0.00332	0.00107	0.00187	0.00075
0.00146	0.00125	0.00152	0.00838	0.00189	0.00206	0.00217	0.00352
0.00127	0.001	0.00017	0.0102	0.00138	0.00094	0.00209	0.00651
0.00119	0.00438	0.01032	0.00438	0.01726	0.00451	0.01461	0.01288
0.00113	0.0041	0.00643	0.00059	0.00363	0.0053	0.00494	0.00761
0.00101	0.00123	0.00141	6E-05	0.00138	0.0021	0	0.00407
0.00092	0.0079	0.01036	0.00398	0.00893	0.01063	0.00321	0.00588
0.00084	0.00028	0.00207	0.00725	0.00511	0.00235	0.00098	0.00134
