# Supplementary material for: A proteomic-informed view of the changes induced by loss of cellular adherence: The example of mouse macrophages
Source: PLoS One. 2021 May 28;16(5):e0252450. doi: 10.1371/journal.pone.0252450 (PMC8162644; doi:10.1371/journal.pone.0252450)
Supplement: S2 Table — (PDF) [file pone.0252450.s005.pdf]

Supplementary Table 1: proteins showing a significant change in abundance between adherent and non-adherent cells

Note: when several spots are present for one protein, they are numbered from basic (least modified) to acidic (most modified)
T test : p value in the Student T-test for the adherent vs non-adherent (susp) comparison
U test : value of the U parameter in the Mann-Whitney U test for the adherent vs non-adherent (susp) comparison

abbreviation	acc. Number	protein name	ratio susp/adh	T test	U test	number unique peptides	number unique spectra	sequence coverage
1433 gam	P61982	14-3-3 protein gamma	0,805746277	0,038220937	2	9	13	55%
A16A1	Q571I9	Aldehyde dehydrogenase family 16 member A1	1,290290621	0,033964803	1	6	6	10%
acadl	P51174	Long-chain specific acyl-CoA dehydrogenase, mitochondrial	1,23682476	0,016347082	0	18	24	38%
aldr/1	P45376	Aldose reductase	1,024381387	0,508618476	6	15	24	50%
aldr/2	P45376	Aldose reductase	0,910241657	0,048068674	1	19	51	58,00%
anxa1/1	P10107	Annexin A1	1,109832882	0,148513318	4	32	177	78%
anxa1/2	P10107	Annexin A1	0,932344086	0,241711193	4	28	49	71%
anxa1/3	P10107	Annexin A1	0,682600382	0,033856401	0	22	32	68%
anxa2/1	P07356	Annexin A2	0,974035335	0,516240908	6	27	197	66%
anxa2/2	P07356	Annexin A2	0,777410299	0,050872156	1	11	12	43%
anxa3/1	O35639	Annexin A3	1,060269536	0,330595549	6	21	41	59%
anxa3/2	O35639	Annexin A3	0,986777625	0,85858004	7	14	23	46%
anxa3/3	O35639	Annexin A3	0,60144848	0,001695145	0	2	2	7%
anxa4/1	P97429	Annexin A4	1,002798404	0,946522399	7	24	56	66%
anxa4/2	P97429	Annexin A4	0,784656596	0,01127691	0	20	33	59%
anxa5	P48036	Annexin A5	0,847587487	0,060208089	1	29	259	83%
arp2/1	P61161	Actin-related protein 2	1,055469716	0,418767836	6	16	38	45,00%
arp2/2	P61161	Actin-related protein 2	0,851684109	0,085751693	0	2	3	5,00%
arpc2/1	Q9CVB6	Actin-related protein 2/3 complex subunit 2	1,09372566	0,219384369	3	13	22	37%
arpc2/2	Q9CVB6	Actin-related protein 2/3 complex subunit 2	0,902338377	0,08679415	2	14	15	59%

arpc2/3	Q9CVB6	Actin-related protein 2/3 complex subunit 2	0,781282232	0,018966595	0	24	50	81%
atg3	Q9CPX6	Ubiquitin-like-conjugating enzyme ATG3	1,887168142	0,023346451	0	7	10	24%
atp5h/1	Q9DCX2	ATP synthase subunit d, mitochondrial	0,919098458	0,052251968	1	10	15	65%
atp5h/2	Q9DCX2	ATP synthase subunit d, mitochondrial	0,830662683	0,02505455	1	7	7	55%
atpb/1	P56480	ATP synthase subunit beta, mitochondrial	1,045320787	0,674951683	6	21	328	48%
atpb/2	P56480	ATP synthase subunit beta, mitochondrial	0,809031375	0,015155216	0	16	59	39%
atpb/3	P56480	ATP synthase subunit beta, mitochondrial	0,841681925	0,034419514	1	9	20	27%
brcc3	P46737	Lys-63-specific deubiquitinase BRCC36	1,122406155	0,048002307	2	3	4	13%
cap1/1	P40124	Adenylyl cyclase-associated protein 1	0,989442259	0,850994718	7	17	24	40%
cap1/2	P40124	Adenylyl cyclase-associated protein 1	0,920536954	0,253387805	5	9	10	22%
cap1/3	P40124	Adenylyl cyclase-associated protein 1	0,872356891	0,054579004	1	13	25	35%
capg/1	P24452	Macrophage-capping protein	0,954318207	0,39525808	5	12	67	49%
capg/2	P24452	Macrophage-capping protein	0,803620331	0,006640936	0	11	21	49%
capg/3	P24452	Macrophage-capping protein	0,828973223	0,079166719	0	11	41	34%
capg/4	P24452	Macrophage-capping protein	0,652293497	0,062780539	1			
catd	P18242	Cathepsin D	1,165939514	0,095649863	0	9	10	28%
caza2	P47754	F-actin-capping protein subunit alpha-2	0,809641382	0,041920974	0	6	7	29%
clic4	Q9QYB1	Chloride intracellular channel protein 4	0,623341362	0,023269451	0	18	39	82%
cof1/1	P18760	Cofilin-1	1,14739805	0,472451744	6	5	6	42%
cof1/2	P18760	Cofilin-1	0,639041584	0,018079856	0	11	51	57%
cof1/3	P18760	Cofilin-1	0,827331811	0,318817499	5	5	6	42%
cof1/4	P18760	Cofilin-1	0,707158772	0,008391953	0	7	10	57%

comd4	Q9CQ02	COMM domain-containing protein 4	1,456414104	0,049624984	1	5	5	31%
ddx39b	Q9Z1N5	Spliceosome RNA helicase Ddx39b	1,221594725	0,032860661	0	16	23	46%
dnm2	P39054	Dynamin-2	1,645305514	0,023665207	0	23	36	32%
eef2/1	P58252	Elongation factor 2	1,370430544	0,043992113	0	13	18	15%
eef2/2	P58252	Elongation factor 2	1,283030536	0,044288928	0	20	33	23%
eef2/3	P58252	Elongation factor 2	1,158265045	0,075441739	2	18	30	22,00%
eef2/4	P58252	Elongation factor 2	1,088915938	0,292777346	4	16	20	18,00%
ef1g	Q9D8N0	Elongation factor 1-gamma	0,779327533	0,051554799	7	18	24	43%
ehd1	Q9WVK4	EH domain-containing protein 1	0,907172241	0,028582869	0	11	12	24%
eno1/1	P17182	Alpha-enolase	1,001976807	0,982901566	8	17	24	47%
eno1/2	P17182	Alpha-enolase	0,928911771	0,139137379	3	22	54	64%
eno1/3	P17182	Alpha-enolase	0,777616175	0,075597705	2	29	145	63%
f10a1	Q99L47	Hsc70-interacting protein	0,669994969	0,037503655	1	7	12	21%
fumh	P97807	Fumarate hydratase, mitochondrial	0,802326196	0,009710581	0	20	39	51%
ganab/1	Q8BHN3	Neutral alpha-glucosidase	1,120645922	0,403337655	7	9	10	11%
ganab/2	Q8BHN3	Neutral alpha-glucosidase	1,084076376	0,452278916	7	9	9	12,00%
ganab/3	Q8BHN3	Neutral alpha-glucosidase	1,250302786	0,021844461	0	7	7	7,00%
ganab/4	Q8BHN3	Neutral alpha-glucosidase	1,789621812	0,022061285	1	23	30	29,00%
gdib/1	Q61598	Rab GDP dissociation inhibitor beta	0,889463541	0,098611926	0	24	33	67%
gdib/2	Q61598	Rab GDP dissociation inhibitor beta	0,794920301	0,033504189	1	10	11	25%
gelsolin/1	P13020	Gelsolin	1,53286248	0,022422743	0	16	26	26%
gelsolin/2	P13020	Gelsolin	1,219009826	0,214220906	5	23	56	38%
gelsolin/3	P13020	Gelsolin	1,192201162	0,458366207	6	17	17	32%
gelsolin/4	P13020	Gelsolin	1,055984005	0,789721106	8	17	34	26%
gipc1	Q9Z0G0	PDZ domain-containing protein GIPC1	1,721153846	0,03512765	0	8	16	30%
gnb2l1	P68040	Guanine nucleotide-binding protein subunit beta-2-like 1	0,870816078	0,039683129	2	8	9	32%
hars	Q61035	Histidine--tRNA ligase, cytoplasmic	1,24427926	0,003303275	0	13	15	26%
hem2	P10518	Delta-aminolevulinic acid dehydratase	1,582741554	0,010215701	0	12	18	38%

hint1	P70349	Histidine triad nucleotide-binding protein 1	1,820138355	0,125209532	0	7	10	78%
hsp74/1	Q61316	Heat shock 70 kDa protein 4	1,263037285	0,19323848	4	24	28	40,00%
hsp74/2	Q61316	Heat shock 70 kDa protein 4	1,196684912	0,283170193	5	34	63	60%
hsp74/3	Q61316	Heat shock 70 kDa protein 4	1,102858744	0,54121719	6			
hsp74/4	Q61316	Heat shock 70 kDa protein 4	1,012521255	0,901292956	8			
hsp74/5	Q61316	Heat shock 70 kDa protein 4	0,542792793	0,03236657	2			
hxk3/1	Q3TRM8	Hexokinase-3	1,369735408	0,27009332	4	45	76	49%
hxk3/2	Q3TRM8	Hexokinase-3	1,670547874	0,010936046	0	49	94	53%
hxk3/3	Q3TRM8	Hexokinase-3	2,132250056	0,090126719	2	52	104	53%
hyou1/1	Q9JKR6	Hypoxia up-regulated protein 1	1,473414591	0,017363138	0	31	48	43%
hyou1/2	Q9JKR6	Hypoxia up-regulated protein 1	1,100830225	0,47379077	6	37	58	48%
hyou1/3	Q9JKR6	Hypoxia up-regulated protein 1	1,146915934	0,401311088	5	28	60	36%
hyou1/4	Q9JKR6	Hypoxia up-regulated protein 1	0,904354148	0,405696792	3			
hyou1/5	Q9JKR6	Hypoxia up-regulated protein 1	0,764350841	0,135039075	3			
ide/1	Q9JHR7	Insulin-degrading enzyme	2,112579186	0,026852722	1	13	15	14%
ide/2	Q9JHR7	Insulin-degrading enzyme	2,623164763	0,003424798	0			
imdh2/2	P24547	Inosine-5'-monophosphate dehydrogenase 2	0,560691509	0,016126227	1	27	40	61%
imdh2/2	P24547	Inosine-5'-monophosphate dehydrogenase 2	1,098279187	0,536553849	6	11	14	21%
impa1/1	O55023	Inositol monophosphatase 1	0,952384954	0,576747661	7	6	9	23%
impa1/2	O55023	Inositol monophosphatase 1	0,725284656	0,07136186	0	2	2	7,00%
kars/1	Q99MN1	Lysine--tRNA ligase	1,322969972	0,027276131	0	25	33	45%
kars/2	Q99MN1	Lysine--tRNA ligase	1,271413558	0,015252139	0	22	32	40%
kpym/1	P52480	pyruvate kinase	0,924990401	0,187914324	3	41	200	77%
kpym/2	P52480	pyruvate kinase	0,864285832	0,035618385	1	24	93	47%
kpym/3	P52480	pyruvate kinase	0,921745088	0,432877766	4	15	16	33%
lars/1	Q8BMJ2	Leucine--tRNA ligase, cytoplasmic	1,977262726	0,014841097	0	10	10	10%
lars/2	Q8BMJ2	Leucine--tRNA ligase, cytoplasmic	1,984055459	0,018868684	0	9	10	10,00%
lhpp	Q9D7I5	Phospholysine phosphohistidine inorganic pyrophosphate phosphatase	1,689076947	0,017372414	0	10	18	71%

lon	Q8CGK3	Lon protease homolog, mitochondrial	1,647734444	0,035930209	1	4	4	5%
lpprc	Q6PB66	Leucine-rich PPR motif-containing protein, mitochondrial	1,626184324	0,084488824	0	44	47	32%
lsp1/1	P19973	Lymphocyte-specific protein 1	0,497064374	0,006293032	0	12	15	52%
lsp1/2	P19973	Lymphocyte-specific protein 1	0,582476266	0,018394536	0	12	15	56%
mcm7/1	Q61881	DNA replication licensing factor MCM7	1,119465743	0,186280018	2	27		46%
mcm7/2	Q61881	DNA replication licensing factor MCM7	1,383358701	0,008625574	0	5	5	8,00%
memo1	Q91VH6	Protein MEMO1	0,78487432	0,0064803	0	3	4	10%
mesd	Q9ERE7	LDLR chaperone MESD	1,395750332	0,059939064	3	3	3	21%
mgn	P61327	Protein mago nashi homolog	1,410411055	0,006786167	0	2	2	14%
mic60/1	Q8CAQ8	MICOS complex subunit Mic60	0,964833349	0,733329108	6	12	12	18%
mic60/2	Q8CAQ8	MICOS complex subunit Mic60	1,164764079	0,572699133	5	11	12	15%
mic60/3	Q8CAQ8	MICOS complex subunit Mic60	1,265350877	0,049540753	2	3	3	4%
mk14	P47811	Mitogen-activated protein kinase 14	1,19424307	0,045686669	1	13	16	57%
mlec	Q6ZQI3	Malectin	1,363332905	0,004910015	0	4	5	15%
mvp/1	Q9EQK5	Major vault protein	1,683879208	0,016811336	0	10	10	12%
mvp/2	Q9EQK5	Major vault protein	1,226394695	0,22303483	3	22	32	29%
mvp/3	Q9EQK5	Major vault protein	1,074903602	0,653053004	8	14	14	18%
mindy3	Q9CV28	Protein FAM188A	2,86949429	0,019865353	0	6	7	24%
nfyc	P70353	Nuclear transcription factor Y subunit gamma	0,195448745	0,068724312	0	2	5	5%
odo1	Q60597	2-oxoglutarate dehydrogenase, mitochondrial	1,641840566	0,038230029	1	13	14	14%
osgep	Q8BWU5	Probable tRNA N6-adenosine threonylcarbamoyltransferase	1,420850348	0,01717367	1	8	12	31%
ostf1	Q62422	Osteoclast-stimulating factor 1	0,752127596	0,018813708	0	6	8	37%
parvg	Q9ERD8	parvin gamma	0,57870	0,06403	1	4	4	15%
pcna	P17918	Proliferating cell nuclear antigen	0,774402825	0,015973057	0	13	31	71%
pdcd5	P56812	Programmed cell death protein 5	2,522699758	0,039801257	0	3	4	29%
pef1	Q8BFY6	Peflin	1,271385776	0,013246967	0	6	9	20%

pgam/1	Q9DBJ1	Phosphoglycerate mutase 1	0,937917032	0,227745925	5	20	179	62%
pgam/2	Q9DBJ1	Phosphoglycerate mutase 1	0,843875261	0,03180944	0	12	21	62%
pgam/3	Q9DBJ1	Phosphoglycerate mutase 1	0,699979299	0,048213737	0	15	46	67%
pgd/1	Q9DCD0	6-phosphogluconate dehydrogenase, decarboxylating	1,005451405	0,925056616	6	5		12,00%
pgd/2	Q9DCD0	6-phosphogluconate dehydrogenase, decarboxylating	0,880636408	0,090247158	0	2		4,00%
plst/1	Q61233	Plastin-2	1,363833635	0,014314383	0	42	264	71%
plst/2	Q61233	Plastin-2	1,227847408	0,001300653	0	30	45	64%
pp1r7	Q3UM45	Protein phosphatase 1 regulatory subunit 7	0,586628553	0,06833637	2	15	17	52%
pp2aa	P63330	Serine/threonine-protein phosphatase 2A catalytic subunit alpha isoform	0,821941229	0,002152052	0	11	21	44%
prx1 ox	P3570	Peroxiredoxin-1	0,766201913	0,079281626	1	2	2	11%
prx2	Q61171	Peroxiredoxin-2	0,888088454	0,139569729	2	8	17	61%
prx3	P20108	Peroxiredoxin-3	1,197634058	0,045979772	0	6	6	24%
prx5	P99029	Peroxiredoxin-5	1,128261448	0,042930822	0	2	3	9%
psb4/1	P99026	Proteasome subunit beta type-4	0,974742595	0,639276555	7	8	14	49%
psb4/2	P99026	Proteasome subunit beta type-4	0,706863965	0,008444089	0	3	3	16%
psd13	Q9WVJ2	26S proteasome non-ATPase regulatory subunit 13	1,17038653	0,037482111	1	23	34	69%
pth2	Q8R2Y8	Peptidyl-tRNA hydrolase 2, mitochondrial	1,795934324	0,014480605	0	6	10	54%
pur2	Q64737	Trifunctional purine biosynthetic protein adenosine-3	1,728560738	0,04778627	1	46	77	60%
pur4/1	Q5SUR0	Phosphoribosylformylglycinamidine synthase	2,238628159	0,003758619	0	17	24	15%
pur4/2	Q5SUR0	Phosphoribosylformylglycinamidine synthase	1,684821786	0,043268932	1	14	20	13%
pur4/3	Q5SUR0	Phosphoribosylformylglycinamidine synthase	1,205001544	0,121256026	4	3	3	3,00%
pygb/1	Q8CI94	Glycogen phosphorylase, brain form	1,317094654	0,016447807	0	27	33	41%

pygb/2	Q8CI94	Glycogen phosphorylase, brain form	1,237790009	0,073115482	3	14	15	20%
rrf2m	Q8R2Q4	Ribosome-releasing factor 2, mitochondrial	1,9	0,037800764	0	20	27	31%
rs12	P63323	40S ribosomal protein S12	1,759074854	0,06652763	0	8	20	55%
sarnp	Q9D1J3	SAP domain-containing ribonucleoprotein	0,584253417	0,02556337	0	5	7	28%
sgta	Q8BJU0	Small glutamine-rich tetratricopeptide repeat-containing protein alpha	1,553229974	0,017345107	0	9	11	34%
shlb1	Q9JK48	Endophilin-B1	1,332818641	0,025127415	1	10	10	29%
snx18	Q91ZR2	Sorting nexin-18	1,498971386	0,000438432	0	2	2	5%
spee/1	Q64674	Spermidine synthase	0,90128458	0,324718434	4	2	2	7%
spee/2	Q64674	Spermidine synthase	0,739985218	0,047817504	2	4	4	11%
srsf1	Q6PDM2	Serine/arginine-rich splicing factor 1	1,279460285	0,060652265	0	15	68	48%
sucb2	Q9Z2I8	Succinyl-CoA ligase [GDP-forming] subunit beta, mitochondrial	1,274178496	0,002696708	0	6	7	18%
tcpe/1	P80316	T-complex protein 1 subunit epsilon	0,7232845	0,051175185	2	4	4	9%
tcpe/2	P80316	T-complex protein 1 subunit epsilon	0,814430095	0,037144936	1	16	24	31%
tcpe/3	P80316	T-complex protein 1 subunit epsilon	0,851779429	0,258144096	4	3	3	6%
tpis/1	P17751	Triosephosphate isomerase	1,142300292	0,231762872	4	12	22	46%
tpis/2	P17751	Triosephosphate isomerase	0,950850518	0,311780003	4	16	53	62%
tpis/3	P17751	Triosephosphate isomerase	0,800866616	0,018641716	0	12	22	55%
tpp2	Q64514	Tripeptidyl-peptidase 2	1,77577885	0,048943389	0	26	26	23%
trap1/1	Q9CQN1	Heat shock protein 75 kDa, mitochondrial	1,310943784	0,003030903	0	17	20	32%
trap1/2	Q9CQN1	Heat shock protein 75 kDa, mitochondrial	1,083730048	0,528130209	6	24	32	44%
tub a/1	P05213	tubulin alpha 1	0,79682914	0,086812104	2	26	191	67%
tub a/2	P05213	tubulin alpha 1	0,832060111	0,090374622	3			
tub a/3	P05213	tubulin alpha 1	0,817641236	0,16319322	4			
tub b/1	P99024	tubulin beta 5	1,045320787	0,674951683	6			
tub b/2	P99024	tubulin beta 5	0,841681925	0,034419514	1	31	342	76%
tub b/3	P99024	tubulin beta 5	0,809031375	0,015155216	0			

txd17	Q9CQM5	Thioredoxin domain-containing protein 17	0,531945373	0,050850608	2	4	10	29%
vdac2	Q60930	Voltage-dependent anion-selective channel protein 2	0,831711008	0,027237451	1	5	6	22%
vigilin/1	Q8VDJ3	Vigilin	1,976534603	0,032147849	1	46	57	41%
vigilin/2	Q8VDJ3	Vigilin	2,091410559	0,036125344	1	19	20	20%
vigilin/3	Q8VDJ3	Vigilin	2,831908191	0,044169553	3	31	38	32%
vma5a	Q99KC8	von Willebrand factor A domain-containing protein 5A	1,557475779	0,080291862	0	32	116	44%
vps29	Q9QZ88	Vacuolar protein sorting-associated protein 29	1,607099018	0,02142162	1	8	15	48%
wdr1/1	O88342	WD repeat-containing protein 1	1,553173424	0,059728061	0	23	28	69,00%
wdr1/2	O88342	WD repeat-containing protein 1	1,393646592	0,002765385	0	19	21	41,00%
wdr1/3	O88342	WD repeat-containing protein 1	0,784254452	0,022550289	1	19	25	49%
wdr61	Q9ERF3	WD repeat-containing protein 61	0,641573034	0,018748575	0	7	10	24%
