# Supplementary material for: A proteomic-informed view of the changes induced by loss of cellular adherence: The example of mouse macrophages
Source: PLoS One. 2021 May 28;16(5):e0252450. doi: 10.1371/journal.pone.0252450 (PMC8162644; doi:10.1371/journal.pone.0252450)
Supplement: S3 Table — (PDF) [file pone.0252450.s006.pdf]

Supplementary table 2: Modulated pathways highlighted by the DAVID annotation tool

Annotation Cluster 1	Enrichment Score: 4,875336572541458				
Category	Term	Count	PValue	Genes	FDR
GOTERM_MF_DIRECT	GO:0003824~catalytic activity	14	2,44E-06	Q8CI94, P70349, P52480, Q9Z2I8, Q9JHR7, P17751, Q9DBJ1, Q64737, P97807, P10518, Q3TRM8, Q8BHN3, P24547, Q64674	0,00324
KEGG_PATHWAY	mmu01130:Biosynthesis of antibiotics	11	3,10E-06	Q5SUR0, Q9DCD0, P52480, Q9Z2I8, Q3TRM8, Q60597, P17182, P17751, Q9DBJ1, P97807, Q64737	0,00360
GOTERM_BP_DIRECT	GO:0008152~metabolic process	13	8,48E-06	Q8CI94, P51174, P52480, Q9Z2I8, Q9JHR7, Q60597, P17751, Q9DBJ1, Q571I9, Q64737, P10518, Q3TRM8, Q8BHN3	0,01288
KEGG_PATHWAY	mmu01100:Metabolic pathways	21	4,91E-04	Q8CI94, P51174, Q9DCD0, P52480, Q9Z2I8, P17182, P17751, Q60597, Q9DBJ1, P26443, Q64737, P97807, P10518, Q5SUR0, Q9DCX2, Q8BHN3, Q3TRM8, O55023, P45376, P24547, Q64674	0,56748

Annotation Cluster 2	Enrichment Score: 4,759494983661082				
Category	Term	Count	PValue	Genes	FDR
GOTERM_MF_DIRECT	GO:0098641~cadherin binding involved in cell-cell adhesion	12	4,23E-07	P58252, P07356, Q8VDJ3, Q9WVK4, P52480, Q9JK48, P24452, P17182, P68040, Q9Z0G0, P10107, Q9D8N0	0,00056

GOTERM_CC_DIRECT	GO:0005913~cell-cell adherens junction	12	6,17E-07	P58252, P07356, Q8VDJ3, Q9WVK4, P52480, Q9JK48, P24452, P17182, P68040, Q9Z0G0, P10107, Q9D8N0	0,00079
GOTERM_BP_DIRECT	GO:0098609~cell-cell adhesion	5	0,0201736	Q8VDJ3, P24452, P17182, Q9Z0G0, Q9D8N0	26,63610
Annotation Cluster 3	Enrichment Score: 4,539317514208334				
Category	Term	Count	PValue	Genes	FDR
UP_KEYWORDS	Annexin	5	1,78E-07	P48036, P07356, P97429, O35639, P10107	0,00022
SMART	SM00335:ANX	5	2,25E-07	P48036, P07356, P97429, O35639, P10107	0,00022
UP_KEYWORDS	Calcium/phospholipid-binding	5	2,57E-07	P48036, P07356, P97429, O35639, P10107	0,00032
INTERPRO	IPR018502:Annexin repeat	5	2,62E-07	P48036, P07356, P97429, O35639, P10107	0,00035
INTERPRO	IPR018252:Annexin repeat, conserved site	5	2,62E-07	P48036, P07356, P97429, O35639, P10107	0,00035
INTERPRO	IPR001464:Annexin	5	2,62E-07	P48036, P07356, P97429, O35639, P10107	0,00035
UP_SEQ_FEATURE	repeat:Annexin 1	5	8,93E-07	P48036, P07356, P97429, O35639, P10107	0,00122
UP_SEQ_FEATURE	repeat:Annexin 3	5	8,93E-07	P48036, P07356, P97429, O35639, P10107	0,00122
UP_SEQ_FEATURE	repeat:Annexin 2	5	8,93E-07	P48036, P07356, P97429, O35639, P10107	0,00122
UP_SEQ_FEATURE	repeat:Annexin 4	5	8,93E-07	P48036, P07356, P97429, O35639, P10107	0,00122

GOTERM_MF_DIRECT	GO:0005544~calcium-dependent phospholipid binding	5	1,33E-04	P48036, P07356, P97429, O35639, P10107	0,17655
GOTERM_MF_DIRECT	GO:0019834~phospholipase A2 inhibitor activity	3	1,82E-04	P07356, O35639, P10107	0,24124
GOTERM_MF_DIRECT	GO:0004859~phospholipase inhibitor activity	3	4,52E-04	P07356, O35639, P10107	0,59773
GOTERM_MF_DIRECT	GO:0048306~calcium-dependent protein binding	4	0,0066961	P07356, P97429, O35639, P10107	8,51827
UP_KEYWORDS	Calcium	10	0,0115127	P48036, P07356, Q8BFY6, P97429, Q9WVK4, Q61233, O35639, Q60597, P13020, P10107	13,31539
GOTERM_MF_DIRECT	GO:0005509~calcium ion binding	9	0,0407637	P48036, P07356, Q8BFY6, P97429, Q9WVK4, Q61233, O35639, P13020, P10107	42,39093
GOTERM_CC_DIRECT	GO:0042383~sarcolemma	3	0,1212073	P48036, P07356, P10107	80,71645
GOTERM_CC_DIRECT	GO:0009986~cell surface	5	0,3980759	P07356, P97429, Q9JHR7, Q9QYB1, P10107	99,84453
Annotation Cluster 4	Enrichment Score: 4,4075104524459				
Category	Term	Count	PValue	Genes	FDR

GOTERM_CC_DIRECT	GO:0005739~mitochondrion	26	1,14E-06	Q60930, P51174, P52480, Q9JHR7, Q8R2Y8, Q60597, P26443, Q9QYB1, Q99MN1, Q61171, Q9CQN1, P47811, Q8R2Q4, P18242, Q8CAQ8, Q61035, Q9Z2I8, P20108, Q6PB66, P97807, P99029, Q9DCX2, Q9JK48, Q8CGK3, Q3TRM8, P68040	0,00145
UP_KEYWORDS	Transit peptide	13	2,85E-06	Q8CAQ8, Q9CQN1, P51174, Q6PB66, P20108, Q9Z2I8, P99029, Q8R2Y8, Q8CGK3, Q60597, Q8R2Q4, P26443, P97807	0,00352
UP_KEYWORDS	Mitochondrion	17	1,72E-05	Q8CAQ8, Q60930, P51174, Q9Z2I8, P20108, Q6PB66, Q8R2Y8, Q60597, P26443, Q9QYB1, P97807, Q9CQN1, P99029, Q9DCX2, Q9JK48, Q8CGK3, Q8R2Q4	0,02119
UP_SEQ_FEATURE	transit peptide:Mitochondrion	12	1,05E-04	Q9CQN1, P51174, Q6PB66, P20108, Q9Z2I8, P99029, Q8R2Y8, Q8CGK3, Q60597, Q8R2Q4, P26443, P97807	0,14401
GOTERM_CC_DIRECT	GO:0005759~mitochondrial matrix	5	0,0155847	Q9CQN1, P51174, Q8CGK3, Q60597, P26443	18,13445
Annotation Cluster 5	Enrichment Score: 4,313672745450267				
Category	Term	Count	PValue	Genes	FDR
KEGG_PATHWAY	mmu01200:Carbon metabolism	10	1,45E-07	Q9DCD0, P52480, Q9Z2I8, Q3TRM8, Q60597, P17182, P17751, P26443, Q9DBJ1, P97807	0,00017

UP_KEYWORDS	Glycolysis	6	4,63E-07	P52480, Q3TRM8, Q60597, P17182, P17751, Q9DBJ1	0,00057
GOTERM_BP_DIRECT	GO:0006096~glycolytic process	6	1,46E-06	P52480, Q3TRM8, Q60597, P17182, P17751, Q9DBJ1	0,00223
KEGG_PATHWAY	mmu01130:Biosynthesis of antibiotics	11	3,10E-06	Q5SUR0, Q9DCD0, P52480, Q9Z2I8, Q3TRM8, Q60597, P17182, P17751, Q9DBJ1, P97807, Q64737	0,00360
KEGG_PATHWAY	mmu00010:Glycolysis / Gluconeogenesis	5	0,0013889	P52480, Q3TRM8, P17182, P17751, Q9DBJ1	1,59886
KEGG_PATHWAY	mmu01230:Biosynthesis of amino acids	4	0,0185241	P52480, P17182, P17751, Q9DBJ1	19,49412
KEGG_PATHWAY	mmu05230:Central carbon metabolism in cancer	3	0,0812778	P52480, Q3TRM8, Q9DBJ1	62,58505

Annotation Cluster 6	Enrichment Score: 4,207371847383979				
Category	Term	Count	PValue	Genes	FDR
UP_KEYWORDS	Nucleotide-binding	26	1,39E-07	P58252, P70349, Q60930, P52480, Q9JHR7, P26443, Q64737, Q99MN1, Q61316, Q5SUR0, Q9CQN1, P61161, Q9WVK4, Q9Z1N5, P47811, P80316, Q61881, Q9JKR6, Q8R2Q4, Q8BMJ2, Q9Z2I8, Q61035, P39054, P99024, Q3TRM8, Q8CGK3	0,00017

GOTERM_MF_DIRECT	GO:0000166~nucleotide binding	28	4,35E-06	P58252, P70349, Q60930, P52480, Q9JHR7, P26443, Q64737, Q61316, Q99MN1, Q9CQN1, Q5SUR0, Q9Z1N5, P61161, Q9WVK4, P47811, P80316, Q61881, Q9JKR6, Q8R2Q4, P24547, Q8BMJ2, Q61035, Q9Z2I8, P39054, Q6PDM2, P99024, Q8CGK3, Q3TRM8	0,00576
UP_KEYWORDS	ATP-binding	19	2,97E-05	P52480, Q61035, Q9JHR7, P26443, Q64737, Q61316, Q99MN1, Q9CQN1, Q5SUR0, Q9WVK4, P61161, Q9Z1N5, P80316, P47811, Q61881, Q9JKR6, Q8CGK3, Q3TRM8, Q8BMJ2	0,03664
GOTERM_MF_DIRECT	GO:0005524~ATP binding	20	5,63E-04	P52480, Q9Z2I8, Q61035, Q9JHR7, P26443, Q64737, Q61316, Q99MN1, Q9CQN1, Q5SUR0, Q9Z1N5, Q9WVK4, P61161, P80316, P47811, Q61881, Q9JKR6, Q8CGK3, Q3TRM8, Q8BMJ2	0,74356
UP_SEQ_FEATURE	nucleotide phosphate-binding region:ATP	10	0,090945	Q5SUR0, P61161, Q9Z1N5, Q9WVK4, P47811, Q61881, Q3TRM8, Q8CGK3, Q64737, Q99MN1	72,90258
Annotation Cluster 7	Enrichment Score: 2,743456753601563				
Category	Term	Count	PValue	Genes	FDR
GOTERM_MF_DIRECT	GO:0003779~actin binding	12	2,76E-06	P40124, P61161, P61982, P47754, Q61233, O88342, P24452, P13020, P18760, P19973, Q9CVB6, Q9Z0G0	0,00366

GOTERM_CC_DIRECT	GO:0005925~focal adhesion	12	4,86E-06	P48036, P40124, P61161, P61982, Q61598, Q61233, Q9JKR6, P39054, P13020, P18760, Q9CVB6, P10107	0,00618
UP_KEYWORDS	Actin-binding	9	1,72E-05	P40124, P61161, P47754, Q61233, O88342, P24452, P13020, P18760, Q9CVB6	0,02128
GOTERM_CC_DIRECT	GO:0001891~phagocytic cup	4	1,37E-04	Q61233, P39054, P68040, P10107	0,17394
GOTERM_CC_DIRECT	GO:0002102~podosome	4	5,69E-04	P61161, Q61233, O88342, P13020	0,72218
GOTERM_MF_DIRECT	GO:0051015~actin filament binding	6	8,49E-04	P58252, P61161, Q61233, O88342, P18760, Q9CVB6	1,11904
GOTERM_CC_DIRECT	GO:0042995~cell projection	10	0,0103526	P48036, P61161, Q9WVK4, Q61233, O88342, P39054, P68040, P18760, Q9CVB6, P10107	12,41543
GOTERM_CC_DIRECT	GO:0005856~cytoskeleton	13	0,0108135	P63330, P61161, P46737, P80316, Q6PB66, Q61233, Q9EQK5, P99024, O88342, P39054, P13020, P18760, Q9CVB6	12,93364
UP_KEYWORDS	Cell projection	9	0,0108423	P61161, Q9WVK4, Q61233, O88342, P39054, P68040, P18760, Q9CVB6, P10107	12,58709
GOTERM_CC_DIRECT	GO:0015629~actin cytoskeleton	5	0,0194195	Q61233, O88342, P13020, P18760, Q9CVB6	22,10532
UP_KEYWORDS	Cytoskeleton	11	0,0212253	P63330, P61161, P46737, P80316, Q61233, P99024, O88342, P39054, P13020, P18760, Q9CVB6	23,25975
GOTERM_CC_DIRECT	GO:0030054~cell junction	5	0,4986125	P07356, Q61233, O88342, P39054, Q9CVB6	99,98484

UP_KEYWORDS	Cell junction	4	0,5677176	Q61233, O88342, P39054, Q9CVB6	99,99680
Annotation Cluster 8	Enrichment Score: 2,592709923835073				
Category	Term	Count	PValue	Genes	FDR
GOTERM_MF_DIRECT	GO:0016491~oxidoreductase activity	11	0,0019322	Q61171, P51174, Q9DCD0, P20108, Q61598, P99029, Q60597, P45376, P26443, P24547, Q571I9	2,53035
UP_KEYWORDS	Oxidoreductase	10	0,0022104	Q61171, P51174, Q9DCD0, P61982, P20108, P99029, Q60597, P45376, P26443, P24547	2,69376
GOTERM_BP_DIRECT	GO:0055114~oxidation-reduction process	11	0,0039025	Q61171, P51174, Q9DCD0, P20108, Q61598, P99029, Q60597, P45376, P26443, P24547, Q571I9	5,76956
Annotation Cluster 9	Enrichment Score: 2,47674691153277				
Category	Term	Count	PValue	Genes	FDR
UP_KEYWORDS	Actin capping	3	0,0025565	P47754, P24452, P13020	3,10948
GOTERM_BP_DIRECT	GO:0051016~barbed-end actin filament capping	3	0,0033872	P47754, P24452, P13020	5,02603
GOTERM_BP_DIRECT	GO:0051693~actin filament capping	3	0,0042881	P47754, P24452, P13020	6,32241
Annotation Cluster 10	Enrichment Score: 2,2403167604049785				
Category	Term	Count	PValue	Genes	FDR
INTERPRO	IPR012336:Thioredoxin-like fold	6	5,56E-04	Q61171, P20108, P99029, Q9CQM5, Q9QYB1, Q9D8N0	0,75114

GOTERM_MF_DIRECT	GO:0008379~thioredoxin peroxidase activity	3	6,31E-04	Q61171, P20108, P99029	0,83288
UP_SEQ_FEATURE	active site:Cysteine sulfenic acid (-SOH) intermediate	3	6,42E-04	Q61171, P20108, P99029	0,87547
GOTERM_MF_DIRECT	GO:0004601~peroxidase activity	4	6,69E-04	Q61171, P20108, P99029, Q9CQM5	0,88281
UP_SEQ_FEATURE	domain:Thioredoxin	4	0,0011577	Q61171, P20108, P99029, Q9CQM5	1,57382
UP_KEYWORDS	Redox-active center	4	0,0012056	Q61171, P20108, P99029, Q9CQM5	1,47764
GOTERM_MF_DIRECT	GO:0051920~peroxiredoxin activity	3	0,0013374	Q61171, P20108, P99029	1,75782
UP_KEYWORDS	Antioxidant	3	0,0019853	Q61171, P20108, P99029	2,42249
GOTERM_BP_DIRECT	GO:0042744~hydrogen peroxide catabolic process	3	0,002587	Q61171, P20108, P99029	3,86044
UP_KEYWORDS	Peroxidase	3	0,0042799	Q61171, P20108, P99029	5,15516
GOTERM_MF_DIRECT	GO:0016209~antioxidant activity	3	0,007798	Q61171, P20108, P99029	9,85394
INTERPRO	IPR013766:Thioredoxin domain	3	0,0164565	Q61171, P20108, P99029	20,13693
GOTERM_BP_DIRECT	GO:0006979~response to oxidative stress	4	0,0366131	Q61171, P20108, P99029, P10518	43,27167
GOTERM_BP_DIRECT	GO:0045454~cell redox homeostasis	3	0,0493952	Q61171, P20108, P99029	53,69378
UP_KEYWORDS	Disulfide bond	7	0,9963539	Q61171, P20108, P99029, Q9CQM5, P13020, P10107, P18242	100,00000
UP_SEQ_FEATURE	disulfide bond	6	0,9990648	Q61171, P20108, P99029, Q9CQM5, P13020, P18242	100,00000

Annotation Cluster 11	Enrichment Score: 2,0783645290550603				
Category	Term	Count	PValue	Genes	FDR
UP_KEYWORDS	Chaperone	6	0,001657	Q9CQN1, P80316, Q9JKR6, Q8BJU0, Q9ERE7, Q99L47	2,02567
GOTERM_BP_DIRECT	GO:0006457~protein folding	5	0,0053728	Q9CQN1, P80316, P99024, Q9ERE7, Q99L47	7,86151
GOTERM_MF_DIRECT	GO:0051082~unfolded protein binding	3	0,0653704	Q9CQN1, P80316, Q99L47	59,17410

Annotation Cluster 12	Enrichment Score: 2,0210843728490935				
Category	Term	Count	PValue	Genes	FDR
UP_KEYWORDS	Protein biosynthesis	6	5,14E-04	P58252, Q61035, Q8R2Q4, Q8BMJ2, Q9D8N0, Q99MN1	0,63186
UP_KEYWORDS	Ligase	7	0,0036132	Q5SUR0, Q61035, Q9Z2I8, Q8BMJ2, Q64737, Q9CPX6, Q99MN1	4,36854
GOTERM_BP_DIRECT	GO:0006412~translation	8	0,0064974	P58252, Q61035, P63323, Q8R2Q4, P68040, Q8BMJ2, Q9D8N0, Q99MN1	9,43214
GOTERM_MF_DIRECT	GO:0003746~translation elongation factor activity	3	0,0098105	P58252, Q8R2Q4, Q9D8N0	12,24691
UP_KEYWORDS	Aminoacyl-tRNA synthetase	3	0,0124368	Q61035, Q8BMJ2, Q99MN1	14,31011
GOTERM_MF_DIRECT	GO:0016874~ligase activity	7	0,0155985	Q5SUR0, Q61035, Q9Z2I8, Q8BMJ2, Q64737, Q9CPX6, Q99MN1	18,80600
GOTERM_BP_DIRECT	GO:0006418~tRNA aminoacylation for protein translation	3	0,0165647	Q61035, Q8BMJ2, Q99MN1	22,42019
GOTERM_MF_DIRECT	GO:0004812~aminoacyl-tRNA ligase activity	3	0,0198375	Q61035, Q8BMJ2, Q99MN1	23,31891

KEGG_PATHWAY	mmu00970:Aminoacyl-tRNA biosynthesis	3	0,0856828	Q61035, Q8BMJ2, Q99MN1	64,61344

Annotation Cluster 13	Enrichment Score: 1,979819568910825				
Category	Term	Count	PValue	Genes	FDR
UP_KEYWORDS	Purine biosynthesis	3	0,0014834	Q5SUR0, P24547, Q64737	1,81519
GOTERM_BP_DIRECT	GO:0006164~purine nucleotide biosynthetic process	3	0,0052873	Q5SUR0, P24547, Q64737	7,74102
KEGG_PATHWAY	mmu00230:Purine metabolism	4	0,1465748	Q5SUR0, P52480, P24547, Q64737	84,08810

Annotation Cluster 14	Enrichment Score: 1,8755783417911998				
Category	Term	Count	PValue	Genes	FDR
INTERPRO	IPR020568:Ribosomal protein S5 domain 2-type fold	4	9,92E-04	P58252, Q9CQN1, Q8CGK3, Q8R2Q4	1,33528
INTERPRO	IPR014721:Ribosomal protein S5 domain 2-type fold, subgroup	3	0,0082846	P58252, Q8CGK3, Q8R2Q4	10,66094
INTERPRO	IPR027417:P-loop containing nucleoside triphosphate hydrolase	7	0,2875349	P58252, Q9Z1N5, Q9WVK4, Q61881, Q8CGK3, P39054, Q8R2Q4	98,98886

Annotation Cluster 15	Enrichment Score: 1,6940902713401533				
Category	Term	Count	PValue	Genes	FDR

UP_KEYWORDS	Hydrolase	17	0,0024575	P70349, Q9D7I5, P63330, Q9JHR7, Q8R2Y8, P39054, Q9DBJ1, Q9CV28, P99026, P46737, Q9Z1N5, Q61881, Q8CGK3, Q8BHN3, O55023, Q64514, P18242	2,99068
GOTERM_MF_DIRECT	GO:0016787~hydrolase activity	17	0,010168	P70349, Q9D7I5, P63330, Q9JHR7, Q8R2Y8, P39054, Q9DBJ1, Q9CV28, P99026, P46737, Q9Z1N5, Q61881, Q8CGK3, Q8BHN3, O55023, Q64514, P18242	12,66591
GOTERM_MF_DIRECT	GO:0042277~peptide binding	4	0,0150753	Q9JHR7, Q91VH6, Q64514, P18242	18,23234
UP_KEYWORDS	Protease	7	0,0342581	P99026, P46737, Q9JHR7, Q8CGK3, Q64514, Q9CV28, P18242	34,95970
GOTERM_BP_DIRECT	GO:0051603~proteolysis involved in cellular protein catabolic process	3	0,038976	P99026, Q9JHR7, Q8CGK3	45,34991
GOTERM_BP_DIRECT	GO:0006508~proteolysis	8	0,0406052	P99026, Q8BFY6, P46737, Q9JHR7, Q8CGK3, Q64514, Q9CV28, P18242	46,74109
GOTERM_MF_DIRECT	GO:0008233~peptidase activity	7	0,0678006	P99026, P46737, Q9JHR7, Q8CGK3, Q64514, Q9CV28, P18242	60,55855

Annotation Cluster 16	Enrichment Score: 1,110771758861848				
Category	Term	Count	PValue	Genes	FDR
UP_KEYWORDS	GTP-binding	6	0,016497	P58252, Q9Z2I8, P99024, P39054, Q8R2Q4, P26443	18,55761
GOTERM_MF_DIRECT	GO:0005525~GTP binding	7	0,020003	P58252, Q9WVK4, Q9Z2I8, P99024, P39054, Q8R2Q4, P26443	23,49027

GOTERM_MF_DIRECT	GO:0003924~GTPase activity	4	0,1108137	P58252, P99024, P39054, Q8R2Q4	78,90943
UP_SEQ_FEATURE	nucleotide phosphate-binding region:GTP	4	0,2656793	P58252, P99024, P39054, Q8R2Q4	98,54313
INTERPRO	IPR027417:P-loop containing nucleoside triphosphate hydrolase	7	0,2875349	P58252, Q9Z1N5, Q9WVK4, Q61881, Q8CGK3, P39054, Q8R2Q4	98,98886
Annotation Cluster 17	Enrichment Score: 1,095819336520641				
Category	Term	Count	PValue	Genes	FDR
GOTERM_CC_DIRECT	GO:0015629~actin cytoskeleton	5	0,0194195	Q61233, O88342, P13020, P18760, Q9CVB6	22,10532
BIOCARTA	m_rhoPathway:Rho cell motility signaling pathway	3	0,0352518	P13020, P18760, Q9CVB6	29,74154
KEGG_PATHWAY	mmu04666:Fc gamma R-mediated phagocytosis	3	0,128194	P13020, P18760, Q9CVB6	79,62743
KEGG_PATHWAY	mmu04810:Regulation of actin cytoskeleton	3	0,4714505	P13020, P18760, Q9CVB6	99,93854
Annotation Cluster 18	Enrichment Score: 1,0784066628199458				
Category	Term	Count	PValue	Genes	FDR
GOTERM_CC_DIRECT	GO:0031410~cytoplasmic vesicle	8	0,0466219	Q91ZR2, Q9WVK4, P99029, Q9JK48, P39054, Q9QYB1, Q9Z0G0, P10107	45,56661
GOTERM_CC_DIRECT	GO:0005768~endosome	7	0,0591103	P07356, Q91ZR2, Q9WVK4, Q9QZ88, P39054, Q9CVB6, P10107	53,98302
UP_KEYWORDS	Cytoplasmic vesicle	6	0,0675571	Q91ZR2, Q9WVK4, Q9JK48, P39054, Q9QYB1, P10107	57,81753

GOTERM_BP_DIRECT	GO:0006897~endocytosis	3	0,2608848	Q91ZR2, Q9WVK4, P39054	98,98922

Annotation Cluster 19	Enrichment Score: 1,017919464040984				
Category	Term	Count	PValue	Genes	FDR
UP_KEYWORDS	mRNA transport	5	5,95E-04	Q6PDM2, Q9Z1N5, Q6PB66, Q9D1J3, P61327	0,73133
GOTERM_BP_DIRECT	GO:0051028~mRNA transport	4	0,0124863	Q6PDM2, Q9Z1N5, Q6PB66, P61327	17,38384
UP_KEYWORDS	RNA-binding	7	0,0519995	Q6PDM2, Q8VDJ3, Q9Z1N5, Q6PB66, Q9D1J3, P24547, P61327	48,26163
GOTERM_CC_DIRECT	GO:0016607~nuclear speck	4	0,0868681	Q6PDM2, Q9Z1N5, Q9D1J3, P61327	68,57691
UP_KEYWORDS	Spliceosome	3	0,0959276	Q6PDM2, Q9Z1N5, P61327	71,19030
UP_KEYWORDS	Transport	13	0,1384309	Q60930, Q91ZR2, Q8VDJ3, Q6PB66, Q9QYB1, Q6PDM2, Q9WVK4, Q9Z1N5, Q9D1J3, Q9DCX2, Q9QZ88, P61327, Q9CPX6	84,09747
GOTERM_MF_DIRECT	GO:0003723~RNA binding	8	0,1434005	P58252, Q6PDM2, Q8VDJ3, Q9Z1N5, Q6PB66, P17182, P24547, P61327	87,14063
GOTERM_CC_DIRECT	GO:0005681~spliceosomal complex	3	0,152268	Q6PDM2, Q9Z1N5, P61327	87,80705
KEGG_PATHWAY	mmu03040:Spliceosome	3	0,2588788	Q6PDM2, Q9Z1N5, P61327	96,90188
UP_KEYWORDS	mRNA splicing	3	0,2897045	Q6PDM2, Q9Z1N5, P61327	98,53200
GOTERM_BP_DIRECT	GO:0008380~RNA splicing	3	0,3811183	Q6PDM2, Q9Z1N5, P61327	99,93195
UP_KEYWORDS	mRNA processing	3	0,3979216	Q6PDM2, Q9Z1N5, P61327	99,80907

GOTERM_BP_DIRECT	GO:0006397~mRNA processing	3	0,5287525	Q6PDM2, Q9Z1N5, P61327	99,99892
Annotation Cluster 20	Enrichment Score: 0,7046617185765809				
Category	Term	Count	PValue	Genes	FDR
GOTERM_MF_DIRECT	GO:0003697~single-stranded DNA binding	4	0,0111062	Q6PB66, Q61881, Q8CGK3, P10107	13,75646
UP_KEYWORDS	DNA-binding	7	0,7273965	P70353, Q6PB66, Q9D1J3, Q61881, Q8CGK3, P17918, P24547	99,99999
GOTERM_MF_DIRECT	GO:0003677~DNA binding	7	0,9520891	P70353, Q6PB66, Q61881, Q8CGK3, P56812, P17918, P24547	100,00000
Annotation Cluster 21	Enrichment Score: 0,6735807999568495				
Category	Term	Count	PValue	Genes	FDR
INTERPRO	IPR011992:EF-hand-like domain	4	0,1504985	Q8BFY6, Q9WVK4, Q61233, Q9CV28	89,03248
INTERPRO	IPR018247:EF-Hand 1, calcium-binding site	3	0,2119264	Q8BFY6, Q9WVK4, Q61233	96,03371
INTERPRO	IPR002048:EF-hand domain	3	0,29891	Q8BFY6, Q9WVK4, Q61233	99,18700
Annotation Cluster 22	Enrichment Score: 0,6288912527841848				
Category	Term	Count	PValue	Genes	FDR
GOTERM_CC_DIRECT	GO:0005768~endosome	7	0,0591103	P07356, Q91ZR2, Q9WVK4, Q9QZ88, P39054, Q9CVB6, P10107	53,98302
GOTERM_CC_DIRECT	GO:0010008~endosome membrane	3	0,127966	Q91ZR2, Q9WVK4, Q9QZ88	82,52273

UP_KEYWORDS	Endosome	4	0,3158497	Q91ZR2, Q9WVK4, Q9QZ88, P10107	99,07587
GOTERM_BP_DIRECT	GO:0006886~intracellular protein transport	3	0,3634619	Q91ZR2, Q9WVK4, Q9QZ88	99,89565
GOTERM_BP_DIRECT	GO:0015031~protein transport	5	0,4076556	Q91ZR2, Q9WVK4, Q61598, Q9QZ88, Q9CPX6	99,96504
UP_KEYWORDS	Protein transport	4	0,4760602	Q91ZR2, Q9WVK4, Q9QZ88, Q9CPX6	99,96565

Annotation Cluster 23	Enrichment Score: 0,6038240169926955				
Category	Term	Count	PValue	Genes	FDR
UP_KEYWORDS	SH3 domain	3	0,2369495	Q62422, Q91ZR2, Q9JK48	96,44628
UP_SEQ_FEATURE	domain:SH3	3	0,2386107	Q62422, Q91ZR2, Q9JK48	97,60830
SMART	SM00326:SH3	3	0,2436882	Q62422, Q91ZR2, Q9JK48	93,44542
INTERPRO	IPR001452:Src homology-3 domain	3	0,2789483	Q62422, Q91ZR2, Q9JK48	98,81064

Annotation Cluster 24	Enrichment Score: 0,5286584678888754				
Category	Term	Count	PValue	Genes	FDR
INTERPRO	IPR020472:G-protein beta WD-40 repeat	3	0,0736343	O88342, P68040, Q9ERF3	64,52916
UP_SEQ_FEATURE	repeat:WD 7	3	0,1593841	O88342, P68040, Q9ERF3	90,72255
INTERPRO	IPR019775:WD40 repeat, conserved site	3	0,1869811	O88342, P68040, Q9ERF3	93,94965
UP_SEQ_FEATURE	repeat:WD 6	3	0,2406833	O88342, P68040, Q9ERF3	97,69594
UP_KEYWORDS	WD repeat	3	0,3159923	O88342, P68040, Q9ERF3	99,07824

UP_SEQ_FEATURE	repeat:WD 5	3	0,3318402	O88342, P68040, Q9ERF3	99,60016
SMART	SM00320:WD40	3	0,3590852	O88342, P68040, Q9ERF3	98,69669
UP_SEQ_FEATURE	repeat:WD 4	3	0,3704592	O88342, P68040, Q9ERF3	99,82307
INTERPRO	IPR001680:WD40 repeat	3	0,3704766	O88342, P68040, Q9ERF3	99,81102
UP_SEQ_FEATURE	repeat:WD 3	3	0,3963871	O88342, P68040, Q9ERF3	99,90053
UP_SEQ_FEATURE	repeat:WD 1	3	0,4062331	O88342, P68040, Q9ERF3	99,92059
UP_SEQ_FEATURE	repeat:WD 2	3	0,4062331	O88342, P68040, Q9ERF3	99,92059
INTERPRO	IPR017986:WD40-repeat-containing domain	3	0,4457323	O88342, P68040, Q9ERF3	99,96634
INTERPRO	IPR015943:WD40/YVTN repeat-like-containing domain	3	0,4988432	O88342, P68040, Q9ERF3	99,99140
Annotation Cluster 25	Enrichment Score: 0,07995550995559211				
Category	Term	Count	PValue	Genes	FDR
UP_KEYWORDS	Transferase	7	0,7543648	Q8CI94, P52480, P47811, Q8BWU5, Q3TRM8, Q64674, Q64737	100,00000
UP_KEYWORDS	Kinase	3	0,8271632	P52480, P47811, Q3TRM8	100,00000
GOTERM_MF_DIRECT	GO:0016740~transferase activity	7	0,837659	Q8CI94, P52480, P47811, Q8BWU5, Q3TRM8, Q64674, Q64737	100,00000
GOTERM_BP_DIRECT	GO:0016310~phosphorylation	3	0,8529304	P52480, P47811, Q3TRM8	100,00000

GOTERM_MF_DIRECT	GO:0016301~kinase activity	3	0,8934474	P52480, P47811, Q3TRM8	100,00000
Annotation Cluster 26	Enrichment Score: 0,042550276758780065				
Category	Term	Count	PValue	Genes	FDR
GOTERM_CC_DIRECT	GO:0005783~endoplasmic reticulum	7	0,6722155	P48036, Q6ZQI3, Q9JK48, Q8BHN3, Q9JKR6, Q9ERE7, Q9D8N0	99,99993
UP_KEYWORDS	Endoplasmic reticulum	4	0,8265025	Q6ZQI3, Q8BHN3, Q9JKR6, Q9ERE7	100,00000
UP_KEYWORDS	Glycoprotein	6	0,9999315	Q6ZQI3, Q61881, Q8BHN3, Q9JKR6, Q9ERE7, P18242	100,00000
UP_SEQ_FEATURE	signal peptide	6	0,9999587	Q6ZQI3, Q8BHN3, Q9JKR6, Q9ERE7, P13020, P18242	100,00000
UP_KEYWORDS	Signal	7	0,9999853	Q6ZQI3, Q8BHN3, Q9JKR6, Q8CGK3, Q9ERE7, P13020, P18242	100,00000
UP_SEQ_FEATURE	glycosylation site:N-linked (GlcNAc,,,)	5	0,9999994	Q6ZQI3, Q8BHN3, Q9JKR6, Q9ERE7, P18242	100,00000
Annotation Cluster 27	Enrichment Score: 0,03831036524763268				
Category	Term	Count	PValue	Genes	FDR
UP_KEYWORDS	Transcription regulation	7	0,8216627	P70353, P70349, Q6PB66, P47811, Q9D1J3, Q9ERF3, Q9CQ02	100,00000
UP_KEYWORDS	Transcription	7	0,8449553	P70353, P70349, Q6PB66, P47811, Q9D1J3, Q9ERF3, Q9CQ02	100,00000
GOTERM_BP_DIRECT	GO:0006351~transcription, DNA-templated	7	0,953441	P70353, P70349, Q6PB66, P47811, Q9D1J3, Q9ERF3, Q9CQ02	100,00000

GOTERM_BP_DIRECT	GO:0045944~positive regulation of transcription from RNA polymerase II promoter	3	0,9753341	P70353, P47811, Q9ERF3	100,00000
GOTERM_BP_DIRECT	GO:0006355~regulation of transcription, DNA-templated	6	0,9964917	P70353, P70349, Q6PB66, P47811, Q9ERF3, Q9CQ02	100,00000
Annotation Cluster 28	Enrichment Score: 5,3486181853805017E-11				
Category	Term	Count	PValue	Genes	FDR
UP_SEQ_FEATURE	transmembrane region	4	1	Q8CAQ8, Q6ZQI3, Q60930, Q9QYB1	100,00000
UP_KEYWORDS	Transmembrane	5	1	Q8CAQ8, P70349, Q6ZQI3, Q60930, Q9QYB1	100,00000
UP_KEYWORDS	Transmembrane helix	4	1	Q8CAQ8, P70349, Q6ZQI3, Q9QYB1	100,00000
GOTERM_CC_DIRECT	GO:0016021~integral component of membrane	5	1	Q8CAQ8, P70349, Q6ZQI3, Q60930, Q9QYB1	100,00000
