# Supplementary material for: A proteomic-informed view of the changes induced by loss of cellular adherence: The example of mouse macrophages
Source: PLoS One. 2021 May 28;16(5):e0252450. doi: 10.1371/journal.pone.0252450 (PMC8162644; doi:10.1371/journal.pone.0252450)
Supplement: S4 Table — (PDF) [file pone.0252450.s007.pdf]

Supplementary Table 3: semi-quantitative peptide analysis by spectral counting in the mono-phosphorylated form of cofilin
Blank cells : not detected

				spectral	counts			
		cof 1P	susp			cof 1P	adh	
**peptides**	gel 1	gel 2	gel 3	gel 4	gel 1	gel 2	gel 3	gel 4
APENAPLK,					1	1	1	1
aSGVAVSDGVIK, n-term: Acetyl (+42,01)				1	2	1		1
asGVAVSDGVIK, n-term: Acetyl (+42,01), s2: Phospho (+79,97) **S3**	4	5	4	4	8	7	8	9
AsGVAVSDGVIK, s2: Phospho (+79,97)								
AVLFcLSEDK, c5: Carbamidomethyl (+57,02)	1	1			1	1	1	2
AVLFcLSEDKK, c5: Carbamidomethyl (+57,02)	1	2	1	2	3	2	2	3
cYEEVK, c1: Carbamidomethyl (+57,02)	2		1		1	2	1	
EDLVFIFWAPENAPLK,							1	1
EILVGDVGQTVDDPYTTF,								1
EILVGDVGQTVDDPYTTFVK,	1	2	1	1	2	2	1	2
EILVGDVGQtVDDPYTTFVK, t10: Phospho (+79,97) **T63**					1			4
HELQANcYEEVK, c7: Carbamidomethyl (+57,02)	1	1			1	1	1	1
HELQANcYEEVKDR, c7: Carbamidomethyl (+57,02)	1	1	1	1	2	1	2	1
IFWAPENAPLK,								1
IILEEGK,	1	1						1
KEDLVFIF,								1
KEDLVFIFWAPENAPLK,	1	2		1	3	1	2	2
LFcLSEDKK, c3: Carbamidomethyl (+57,02)					1	1	1	1
LGGSAVISLEGK,	1	1		1	1	2	1	1
LGGSAVISLEGKPL,	2	4	2	3	7	5	4	5
LGGsAVISLEGKPL, s4: Phospho (+79,97) **S156**				1	1	1	1	2
LGGSAVIsLEGKPL, s8: Phospho (+79,97) **S160**						1		
LPDKDcR, c6: Carbamidomethyl (+57,02)					1	1		1
MIYASSK,	1	1	1	1	2	1	2	2
mIYASSK, m1: Oxidation (+15,99)					1	2		2
MLPDKDcR, c7: Carbamidomethyl (+57,02)	1	1		2	3	3	2	3
mLPDKDcR, m1: Oxidation (+15,99), c7: Carbamidomethyl (+57,02)								
NIILEEGK,			1	1	1	1	1	3
NIILEEGKEILVGDVGQTVDDPYTTFVK,	1	1			2		1	2

NIILEEGKEILVGDVGQtVDDPYTTFVK, t18: Phospho (+79,97) **T63**								1
sGVAVSDGVIK, s1: Phospho (+79,97)					1			
SSTPEEVK,								1
sSTPEEVK, s1: Phospho (+79,97) **S23**					2		2	1
sSTPEEVKK, s1: Phospho (+79,97) **S23**					2	1	1	
SsTPEEVKK, s2: Phospho (+79,97) **S24**							1	
VDDPYTTFVK,								1
YALYDATYETK,	3	2	2	2	9	6	7	8
yALYDATYETK, y1: Phospho (+79,97) **Y82**					4	5	4	4
