# Supplementary material for: A proteomic-informed view of the changes induced by loss of cellular adherence: The example of mouse macrophages
Source: PLoS One. 2021 May 28;16(5):e0252450. doi: 10.1371/journal.pone.0252450 (PMC8162644; doi:10.1371/journal.pone.0252450)
Supplement: S1 File — (PDF) [file pone.0252450.s008.pdf]

Supplementary material for

A proteomic-informed view of the changes induced by loss of cellular adherence: the example of mouse macrophages

Sacnite Ramirez-Rios [1*], Anaelle Torres [2*], Hélène Diemer [3], Véronique Collin-Faure [2], Sarah Cianférani [3], Laurence Lafanechère [1], Thierry Rabilloud [2§]

1 Institute for Advanced Biosciences, Univ. Grenoble Alpes, CNRS UMR 5309, INSERM U1209; Grenoble, France

2 Chemistry and Biology of Metals, Univ. Grenoble Alpes, CNRS UMR5249, CEA, IRIG-DIESE-CBM-ProMD, Grenoble, France

3 Hubert Curien Multi-disciplinary Institute, Laboratoire de Spectrométrie de Masse BioOrganique (LSMBO), Université de Strasbourg, CNRS, IPHC UMR 7178, 67000 Strasbourg, France

S1 Figure: raw 2D gel images, adherent cells
S2 Figure: raw 2D gel images, non-adherent cells
S3 Figure: complementary annotated 2D gel images
S1 Table: raw results from the quantitative analysis of the 2D gels
S2 Table: proteins showing a significant change in abundance between adherent and non adherent cells
S3 Table: Modulated pathways highlighted by the DAVID annotation tool
S4 Table: semi-quantitative peptide analysis by spectral counting in the mono-phosphorylated form of cofilin
